# Genome-Wide Identification and Expression Analysis of the Basic Leucine Zipper (bZIP) Transcription Factor Gene Family in *Fusarium graminearum*

**DOI:** 10.3390/genes13040607

**Published:** 2022-03-28

**Authors:** Sarfaraz Hussain, Bowen Tai, Athar Hussain, Israt Jahan, Bolei Yang, Fuguo Xing

**Affiliations:** 1 Key Laboratory of Agro-Products Quality and Safety Control in Storage and Transport Process, Ministry of Agriculture and Rural Affairs/Institute of Food Science and Technology, Chinese Academy of Agricultural Sciences, Beijing 100193, China; sarfraz1412@gmail.com (S.H.); taibowen@caas.cn (B.T.); isratanu35@gmail.com (I.J.); yanbo856@163.com (B.Y.); 2 Genomics Lab, School of Food and Agricultural Sciences (SFAS), University of Management and Technology (UMT), Johar Town, Lahore 54782, Pakistan; atharmutahari@gmail.com

**Keywords:** *Fusarium graminearum*, bZIP, phylogenetic, abiotic stress, expression analysis

## Abstract

The basic leucine zipper (bZIP) is a widely found transcription factor family that plays regulatory roles in a variety of cellular processes including cell growth and development and various stress responses. However, the bZIP gene family has not been well studied at a genome-wide scale in *Fusarium graminearum (Fg)*, a potent pathogen of cereal grains. In the present study, we conducted a genome-wide identification, characterization, and expression profiling of 22 *F. graminearum* bZIP (*FgbZIP*) genes at different developmental stages and under various abiotic stresses. All identified *FgbZIPs* were categorized into nine groups based on their sequence similarity and phylogenetic tree analysis. Furthermore, the gene structure analysis, conserved motif analysis, chromosomal localization, protein network studies, and synteny analysis were performed. The symmetry of the exon and intron varied with the phylogenetic groups. The post-translational modifications (PTMs) analysis also predicted several phosphorylation sites in FgbZIPs, indicating their functional diversity in cellular processes. The evolutionary study identified many orthogroups among eight species and also predicted several gene duplication events in *F. graminearum*. The protein modeling indicated the presence of a higher number of α-helices and random coils in their structures. The expression patterns of *FgbZIP* genes showed that 5 *FgbZIP* genes, including *FgbZIP_1.1*, *FgbZIP_1.3*, *FgbZIP_2.6 FgbZIP_3.1* and *FgbZIP_4.3*, had high expression at different growth and conidiogenesis stages. Similarly, eight genes including *FgbZIP_1.1*, *FgbZIP_1.6*, *FgbZIP_2.3*, *FgbZIP_2.4*, *FgbZIP_4.1*, *FgbZIP_4.2*, *FgbZIP_4.3* and *FgbZIP_4.6* demonstrated their putative role in response to various abiotic stresses. In summary, these results provided basic information regarding FgbZIPs which are helpful for further functional analysis.

## 1. Introduction

*F. graminearum* is a mycotoxin-producing pathogen that is equally harmful to humans, animals, and crops [1]. *Fg* is a phytopathogenic fungus that causes Fusarium head blight (FHB), a devasting disease in small-grain cereals including wheat, barley, oats and maize [2,3]. The epidemic of FHB results in a severe economic loss for cereal crop farmers due to a significant reduction in the size and weight of grains resulting in low yield quantity as well as quality. In addition to the reduction in yield, it also contaminates cereals with a wide range of mycotoxins, causing serious health issues to humans and animals [4]. Thus the management and control of FHB in cereal crops is very essential to prevent agricultural losses, which is a big challenge for crop scientists [5]. So, finding and characterizing growth and developmental associated genes in *F. graminearum* might be helpful to understanding the FHB disease.

Transcription factors (TFs) are proteins that bind to DNA regulatory sequence to control the expression of genes [6]. These are critical elements which decide when and in which tissues genes should be express which alter the cellular functions [7]. Many transcription factors have been identified and several of them also functionally characterized. These identified TFs are classified into different families based on their functions and structures, for example, ERF, AP2, DRE, WRKY, HD-ZIP and so forth. Each TFs family performs some specific functions during gene expression [8,9]. among these TFs, the bZIP protein family is one of the diverse and most abundant transcription factors, broadly found in eukaryotes. This gene family performs vital tasks in the development and various stress responses [10,11]. The bZIP proteins can be recognized based on a 60 to 80 amino acids highly conserved region known as bZIP motif, consisting of two prominent regions called basic-region and leucine-zipper-region [12]. The basic region is composed of almost 16 amino acid residues with an unchanging N-x7-R/K pattern [13,14], helping in the nuclear signaling and sequence-specific DNA binding. The leucine-zipper facilitates hetero- or homodimerization of bZIP genes and is less conserved, which consists of various repetitions of Leucine or other hydrophobic amino acids. Along with the bZIP domain, several other patterns including glutamine-rich region, proline-rich region, and acidic rich regions were also observed, demonstrating the role of bZIPs in in transactivation activities [15,16].

The bZIP family is abundant in animals, plants, and microorganisms [10,17,18] with a wide range of activities including different biological functions such as growth and development, amino acid synthesis, nutrient utilization, unfolded protein response (UPR), and several stress responses [19,20]. In addition to cell development, the bZIP (*FlbB*) gene is also involved in optimal asexual development in *Aspergillus nidulans* and *Aspergillus fumigatus* [21,22], while in *Neurospora crassa*, the bZIP gene *CPC-1* involved in amino acid biosynthesis or cross-pathways [23]. The bZIPs are also involved in secondary metabolism and oxidative stress response in *Aspergillus nidulans*, *Aspergillus fumigatus*, *Pestalotiopsis fici*, and *F. graminearum* [24,25,26]. With the help of bZIP-dependent regulation pathways, Fungi also utilize major nutrients like amino acids, iron, sulfur, and nitrogen compounds for their cell growth. For instance, the *HapX is required* for iron homeostasis [27], while *MEAB* regulates nitrogen metabolite in *A. nidulans* [28]. Similarly, the *FpAda1*, *CgAP1*, *Moatf1* and *Hxl1* are involved in growth, conidiation [29], oxidative stress tolerance [30] and virulence [31] in *Fusarium pseudograminearum*, *Magnaporthe oryzae* and in *Cryptococcus neoformans*, respectively. Furthermore, the *HAC-1* is responsible for the unfolded protein response and is required for cellulose formation [32] and the sulfur metabolism, secondary metabolite production and autophagy regulations were also observed by *metR*, *Cys-3* and *SmJLB1* bZIPs in *A. fumigatus*, *A. nidulans* [33], *N. crassa* [34], *A. fumigatus* [35] and in *Sordaria macrospora*, respectively [36]. Even the important role of bZIPs in fungi, plants and animal, very little is known in *Fusarium graminearum*, one of a potent pathogen in cereal crops. The literature survey explored the functional role of *FgbZIP_1.4* (*ZEB2*) (controls the expression of zearalenone ZEN biosynthesis gene cluster pathway) [37], *FgbZIP_2.1* (*Fgap1*) (mediates oxidative stress response and trichothecene biosynthesis) [38] and *FgbZIP_1.2 (ZIF1)* (associated with virulence of *Fg*) in *Fusarium graminearum* [39]. However, a genome-wide identification and characterization of FgbZIPs are essential for deep understanding of FgbZIPs.

Genome-wide study is an advanced approach that help to identify gene families and is applied on several fungal species including such as *Coniothyrium minitans* [40], *Ustilaginoidea virens* [41] and *Magnaporthe oryzae* [42] to identify bZIP gene families. Thus, in this study, we have reported and performed genome-wide systematic identification, characterization and expression profiling of 22 bZIP genes in *F. graminearium* genome. The analysis included evolutionary and diversity analysis, gene structure analysis, conserved domain or motif analysis, prediction of three-dimensional protein structure homology modeling. We reported the expression of *F. graminearium* bZIP (FgbZIP) genes in five stages of growth and conidial development. Furthermore, we analyzed the expression of these genes in response to several abiotic stresses. These systematic analyses pave the way for more investigation into the biological roles of bZIP TFs in *F. graminearum*.

## 2. Materials and Methods

### 2.1. Identification and Physicochemical Analysis of bZIP TFs in F. graminearum

We collected all bZIP proteins available at the Fungal Transcription Factor Database (FTFD; http://ftfd.snu.ac.kr, accessed on 24 August 2021). A total of 46 bZIP encoding proteins were found. These proteins were further screened through CDD platform (https://www.ncbi.nlm.nih.gov/Structure/ cdd/wrpsb.cgi, accessed on 24 August 2021) and SMART (http://smart.embl-heidelberg.de/smart/set_mode.cgi?NOR MAL=1, accessed on 24 August 2021) and excluded proteins with multiple transcripts for single locus (multiple polypeptides coding by single genomics locus were considered a single gene and selected the longest polypeptide chain) and truncated or partial domain, remaining 22 loci with full-length peptide chain. Individual genes were searched on NCBI and new annotations were assigned, for example, *FgbZIP_1.4* and *FgbZIP_4.1* to FGSG_02398 and FGSG_06651, respectively, where the digits before the point reflect the chromosome number and after the point represent the bZIP member number in ascending order. The ExPASy server (http://www.expasy.ch/tools, accessed on 26 august, 2021) was to study the physiochemistry of bZIP proteins including amino acid (a.a) length, molecular weight (Da), and isoelectric point (Ip), as shown in (Table 1). While protein phosphorylation sites are predicted through Netphos 3.1 server [43].

### 2.2. Evolution, Diversity, and Species Tree Analysis of bZIP in Fungal Species

Amino acid sequences of the 22 recognized bZIP members were aligned using the MAFFT approach in Unipro UGene software [44] with slight modifications to detect the conserved sites. Amino acid sequences of 135 bZIP genes from seven model fungi were downloaded from FTFD/NCBI for evolutionary studies (24 from *A. nidulans*, eight from *Ashbya gossypii*, 16 from *N. crassa*, 17 from *Sclerotinia sclerotiorum*, 10 from *Ustilago maydis*, 27 from *S. cerevisiae*, and 11 from *Schizo saccharomyces pombe*). To provide the understanding of evolution and diversity in bZIP proteins among fungal species, we have used an advance comparative genomics, OrthoFinder tool. The DIAMOND tool was used for fast sequence similarity searches. The graph clustering was done with the MCL clustering algorithm. The gene tree inference and distance matrix were of the orthogroups were constructed with DendroBLAST. A distance-based phylogeny tree was constructed using FastME 2.0. For multiple sequence alignment, MAFTT 7.0 was used. The maximum likelihood phylogenetic tree of large alignment was constructed using FastTreeMP with 1000 bootstrap values. Species based phylogenetic tree was also constructed with the same method. The tree was further refined using PowerPoint and CorelDRAW.

### 2.3. Gene Structure Analysis and Chromosomal Distribution of FgbZIP Genes

The Gene Structure Display Server (GSDS) 2.0 tool was used for the examination of the gene structure [45]. All FgbZIP genes were distributed to *F. graminearium* chromosomes based on information available at the NCBI database and are illustrated using TBtools software [46] attached in Supplementary Figure S1.

### 2.4. Examination of FgbZIP Protein Motifs and Subcellular Localization

Conserved domains were identified by the Pfam website, and the conserved de-novo motifs were detected by MEME V5.0.5 discovery server (http://meme-suite.org/tools/meme, accessed on 3 September 2021) with a maximum of 10 motifs and were graphically displayed with TBtools software (Version No. V1.086). The cellular localization was predicted by Wolfpsort and Euk-mPLoc [47].

### 2.5. Protein-Protein Interaction Network and Protein Homology Modeling

STRING software was used to build a network of functionally interacting proteins with employed parameters set at a 0.15 threshold. For homology modeling of FgbZIP proteins, Pymol and SWISS-MOLD (https://www.swissmodel.expasy.org/, accessed on 12 September 2021) [48], were used which are freely available.

### 2.6. Biological Sample, Culture Conditions, Abiotic Treatments, and Total RNA Extractions

Plugs of fungus grown on a cellophane membrane on Petri dishes for 4 days were used to grow *Fg* in 250 mL of potato dextrose broth (PDB) flasks for 7 days at 28 °C. They were collected in a shaking incubator after filtering them through folded sterile cotton gauze and were exposed to various abiotic stress factors for five hours in already prepared stress-related media. Eight abiotic stress agents were applied, including osmotic stress (1 mol/L sorbitol), cell wall stress (0.05% SDS), ionic stress (1 mol/L NaCl), redox stress (30 mmol/L H_2_O_2_), two kinds of pH stress (High pH 9 and Low pH 3), and two levels of temperature shock (high temperature, 37 °C and low temperature, 4 °C). Mycelia in PDB without any treatment taken as the control. After the stress treatments, mycelia were collected by filtering through sterile gauze and frozen in liquid nitrogen and were later used for RNA extractions. Three biological replicates were taken for each treatment.

The expression status of bZIP genes at distinct stages of growth and conidial expansion were observed according to the method described by [49] with little modification. On 90 mm agar plates above a sterilized cellophane membrane disk (Cat. 165-1779, Biorad) 5 mm plugs from a young growing culture of *F. graminearum* were inoculated and incubated at 28 ± 1 °C. Later young growing mycelia of Fg were harvested during its five stages (48, 60, 72, 84 and 96 h) of growth and flash-frozen in liquid nitrogen and grind the mycelia to obtain a fine powder. Total RNA was extracted from the mycelial samples according to the instructions from manufacturers using the LanEasy total RNA Kit (Beijing lanyi Technology Co., Ltd., Beijing, China). NanoDrop 1000 Spectrophotometer (Thermo Scientific, Waltham, MA, USA) and Agarose gel electrophoresis was used to estimate concentration and check the quality of the total RNA.

### 2.7. cDNA Synthesis and Quantitative Polymerase Chain Reaction (RT-qPCR)

The cDNA was synthesized from 7 µL of RNA by following the manufacturer’s instructions of the TransScriptR reagents Kit with gDNA removal (transgen Biotechnology Beijing Co., Ltd.). RT-qPCR was performed using SYBR Green™ Premix Ex Taq™ II and the ubiquitin conjugating enzyme (FG10805) was used as a reference gene in each PCR reaction [50]. Technical samples were in triplicates in each reaction, and each experiment was carried out separately three times. qPCR was performed according to the conditions mentioned previously by [51]. Supplementary File S3 lists all the primers used in this experiment. While primers were obtained from the primer 3 platforms. The comparative expression was calculated by using ∆Ct method [∆Ct = (Ct target gene − Ct Actin gene) treatment − (Ct target gene − Ct Actin gene) control] [52]. The relative expression was displayed in a heatmap using TBtools (v0.66836) by using certain values of gene expressions (Supplementary File S4) obtained to determine the significance of gene expression variations.

## 3. Results

### 3.1. Gene Features and Physiochemistry of bZIPs

After screening, we found a total of 22 putative bZIP genes in *Fg* (Table 1). These genes were further used for additional bioinformatics and physiochemistry analysis including gene naming, chromosomal mapping, gene positions, molecular weight, isoelectric point, sequence length, and intron–exon distributions. For instance, the size of genomic sequence ranged from 786 bp (*FgbZIP_1.3*) to 2519 bp (*FgbZIP_1.6*) with peptide length 165 (*FgbZIP_4.3*) to 612 (*FGbZIP_3.2*) amino acid (a.a). Similarly, molecular weight fluctuated from 18.01 kDa (*FgbZIP_4.3*) to 66.62 kDa (*FgbZIP_1.1*) with isoelectric point from 4.49 (*FgbZIP_1.2*) to 9.960 PI (*FgbZIP_4.1*). Furthermore, most of the bZIP were localized in the nucleus while some (*FgbZIP_1.6*, *FgbZIP_4.5*, and *FgbZIP_3.3)* were also localized in the endoplasmic reticulum and cytoplasm. Additionally, the highest number of phosphorylation sites was predicted in *FgbZIP_3.2* (60 sites) and minimum in *FgbZIP_4.4* (13 sites). It is also observed that 70% of the *FgbZIPs* contain 20 or more phosphorylation sites. *Ser* sites were higher in a regular manner followed by *Thr* and *Tyr*, in most of the FgbZIPs, except in a few where Tyr phosphorylation sites are more than Thr sites (Table 1).

### 3.2. Multiple Sequence Alignment and Evolutionary Analysis

**Figure 1:**
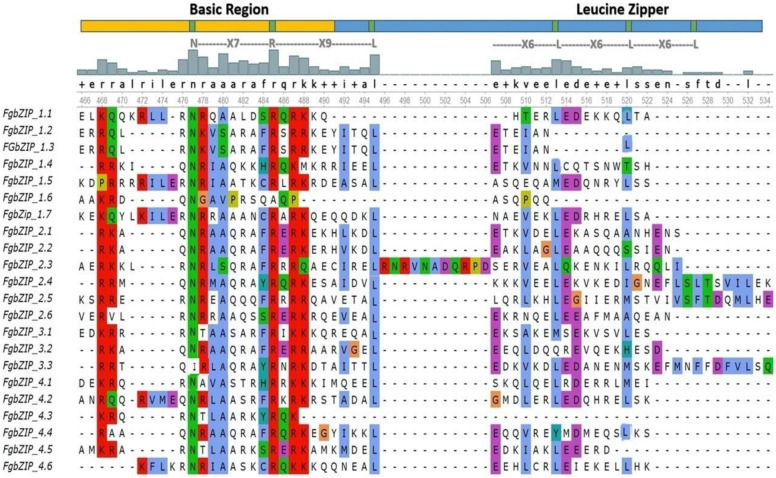
Multiple Sequence alignment of FgbZIP amino acids performed by MAFFT algorithm using UGENE. The representative model of both basic (yellow strip) and leucine zipper (blue strip) region are shown above by highlighting and labelling. Green, red and blue colored amino acids indicate conserved amino acid residues of bZIP gene family. While contrast colors in the conserved region depicts the position replaced by other less conserved amino acids.

The evaluation of *FgbZIP* protein sequences showed that many of the regions have conserved amino acid domains. The classic bZIP domain is made up of a highly conserved basic region with an invariant N-X7-R/K motif and a changeable leucine-zipper region or a heptad repeat of varied bulky hydrophobic amino acids [53]. The properties of the identified bZIP domains in *F. graminearum* were elucidated by aligning amino acid sequences with the specific bZIP domains. Almost all the potential bZIP members have the same invariant residue pattern (N-X7-R/K) and the same leucine-zipper region. Some exceptions were observed in a few *FgbZIP* genes. Most *FgbZIPs* accommodate the basic region, while the leucine zipper section lacked repeated heptads of the leucine (L) or other hydrophobic amino acids. On the other side, *FgbZIP_1.6* and *FgbZIP_3.3* lacked complete conserved motifs invariant of the basic region (N-X7-R/K/l). The N-X7-R motif was more common than the N-X7-K motif, indicating that arginine (R) was more conserved in the FgbZIP domains than Lysine (K). Unexpectedly, the core asparagine (N) residue was replaced by isoleucine (I) in one of 22 *FgbZIP* gene domains, while arginine (R) was replaced by alanine (A) in one gene on the basic region. The first leucine (L) was more conserved in the leucine zipper part than the later leucine (L) conserved region, which had been frequently substituted with other bulky hydrophobic amino acids such alanine (A), isoleucine (I), serine (S) and methionine (M) (Figure 1).

**Figure 2:**
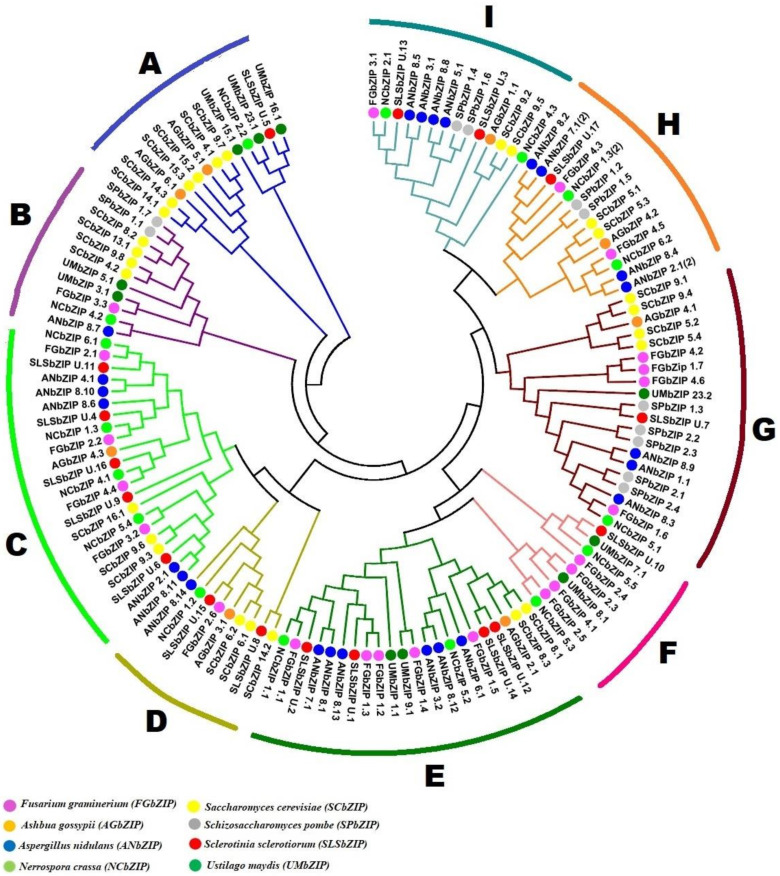
Phylogenetic tree constructed using 135 full-length bZIP protein sequences. The phylogenetic tree was constructed in MEGAX using maximum likelihood method after aligning with the ClustalW program and the bootstrap iterations value was 1000 to generate tree. The proteins are clustered into nine clades (**A**–**I**) indicated by colored branches. The FgbZIPs are denoted by pink circles while. Ashbya gossypii, Aspergillus nidulans, Neurospora crassa, S. cerevisiae, Schizosaccharomyces pombe, S. sclerotiorum and Ustilago maydis are indicated with orange, blue, green, yellow, silver, red and dark green colored circles respectively.

A phylogenetic investigation was conducted based on a total of 135 bZIP proteins including 22 *FgbZIP* and 113 bZIP protein members belonging to other model fungi to examine the evolutionary associations among the *FgbZIP* genes and representative fungal bZIP genes (Figure 2, File S1). All 135 bZIP TFs were classified into nine subgroups (allocated from A to I). The bZIP genes do not demonstrate a great variation among the genomes of different fungal species. The bZIP genes from eight (8) fungi involving the 22 *FgbZIPs* were almost scattered consistently to all sister clades, demonstrating that these bZIP genes were evolved before the divergence of the included fungal species. Four FgbZIP proteins in group F (FgbZIP_2.3, FgbZIP_2.4, FgbZIP_2.5, and FgbZIP_4.1) were found to be more closely related than other bZIPs, implying that a genome duplication event occurred recently. While the number of bZIP proteins in *S. cerevisiae* is more than that of other species, suggesting an evolutionarily significant expansion event. The specific distribution of the FgbZIP proteins was as C, F, and G groups contain four members each. While group B, D, and I contain one member each, likewise E (5), H (2) and group A contains no *FgbZIP* member.

The evolutionary study of bZIP among eight species also provided insight into genome evolution, gene duplication, orthologs duplication, and species phylogenetic tree. The study included a total of 135 bZIPs from eight species. All these genes were divided into 18 orthogroups covering 125/135 genes with 92% of total genes.

The individual orthogroup consists of different gene numbers, for example, orthogroup zero (OG0) has 16 genes followed by OG1 (16 genes), OG2 (15 genes), OG3 (nine genes), OG4 (eight genes), OG5 (seven genes), OG6 (seven genes), OG7 (six genes), OG8 (six genes), OG9 (six genes), OG10 (five genes), OG11 (five genes), OG12 (five genes), OG13 (three genes), OG14 (three genes), OG15 (three genes), OG16 (three genes) and OG17 (two genes) orthogroup.

In addition, we observed only a single species-specific orthogroup with only two genes. Interestingly, there were only three shared orthogroups among all species. So, some orthogroups were clade-specific and some were sister-taxa-specific. The species phylogenetic tree was divided into two major clades. The first clade consisted of *Spb*, *Unm*, *Agb*, and *Scb* while the 2nd clade comprised *Feb*, *Ncb*, *Anb*, and *SLs*. The species-wise evolution also provided detailed evolutionary relationships among eight species. For instance, the *Agb* species had 8/8 bZIP genes within orthogroups (100 % of genes). Similarly, *Anb* has 23 genes in orthogroups (95.8% of genes) and one species-specific gene, in *Ncb* species 13 genes were orthogroups (81.2 % of genes) and three genes were species-specific. In the case of *Fgb*, a total of 20 genes shared orthgroups with other fungal species; however, only two *Fgb* species-specific genes were also observed.

**Figure 3:**
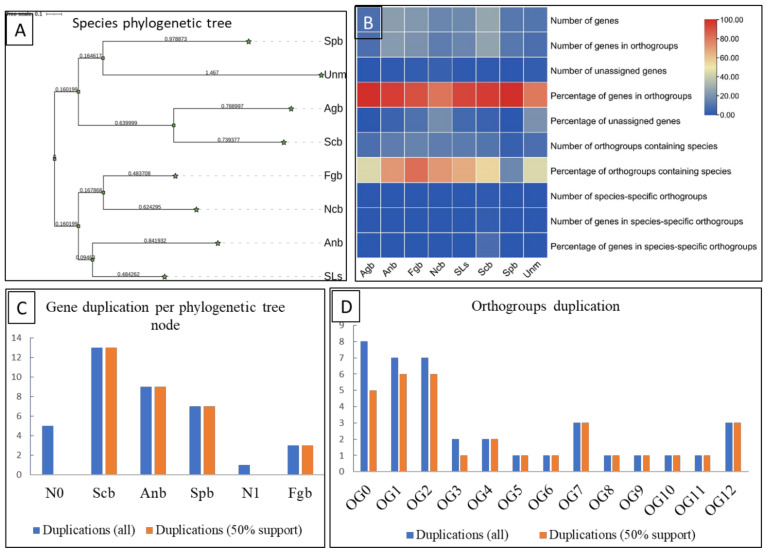
Evolutionary study of bZIP in Fungal species. (**A**); bZIP Based phylogenetic tree of species *Schizo saccharomyces pombe* (Spb), *Ustilago maydis* (Unm), *Ashbya gossypii* (Agb), *S. cerevisiae* (Scb), *Fusarium graminearum* (Fgb), *N. crassa* (Ncb), *A. nidulans* (Anb), *Sclerotinia sclerotiorum* (SLs), (**B**); Orthologs summary, (**C**); Gene duplication events among fungal species, (**D**); orthogroups duplication events in all 135 genes.

The gene duplication events per phylogenetic tree node also depicted several duplications at internal as well as an external nodes. The duplication event was calculated at 100% and 50% duplication support. The highest gene duplication was found at *Scb* (13 duplications), followed by *Anb* (nine duplications), *Spb* (seven duplications), and the *Fgb* (three duplications). Similarly, several orthogroups duplication events were also observed (Figure 3, Supplementary File S2).

### 3.3. Gene Structure Analysis and Chromosomal Distribution of FgbZIP Genes

**Figure 4:**
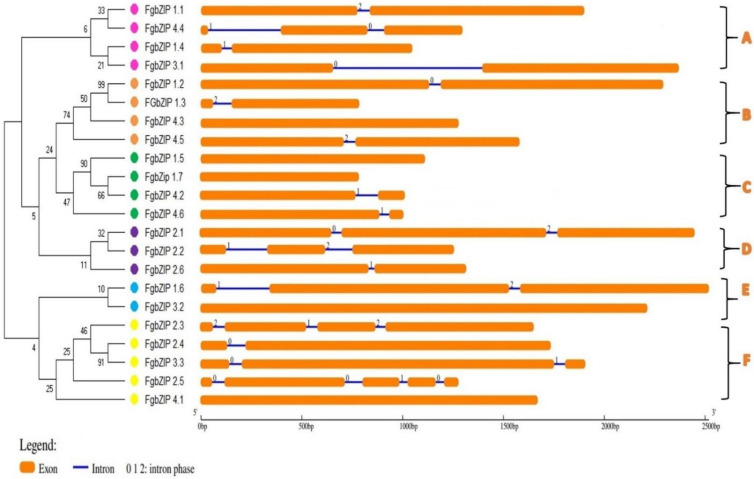
*Gene structure of F. graminearum bZIP* genes it displays exons, intron and intron phase on a bp scale. The phylogenetic tree on the left was constructed based on the full-length sequences of the FgbZIP proteins. While the alphabets A-F shows groups.

Full length genomic sequences of *FgbZIPs* with their CDS sequences were utilized to elaborate the structural characteristics using GSDS Tool (Figure 4). The exon-intron was unevenly distributed throughout the genomic sequence and the number of exons varied from gene to gene. Some *FgbZIP* TFs contains only a single exon (*FgbZIP_4.3*, *FgbZIP_1.5*, *FgbZIP_3.2*, *FgbZIP_1.7* and *FgbZIP_4.1*) while others have at least one intron. The F subgroup contains zero to four introns, while group A possessed one or two introns, the B group members mostly have zero or one introns followed by group C, the D group with one and two introns, and finally the E group with zero to two introns. A similar *FgbZIP* gene has the same intron-exon structure and intron phase patterns. Intron sizes ranged from 44 to 588 nucleotides, and more than 60% of the introns were between 45 to 100 bp.

The chromosomal distribution of 22 genes *FgbZIP* genes was predicted graphically according to the available information. According to the comparison, chromosome 1 has the most genes (seven genes), followed by chromosomes 2 and 4 (six genes each, respectively) and chromosome 3 has three bZIP members. Most of the *FgbZIP* genes were found in clusters and located at terminals of chromatids. It is also observed that genes from the same groups were also located on the same chromosome, for example, *FgbZIP_2.1*, *FgbZIP_2.2* and *FgbZIP_2.6* belong to D-group and *FgbZIP_2.3*, *FgbZIP_2.4* and *FgbZIP_2.5* from F-group found in clusters (Figure S1).

### 3.4. Conserved Domains and Motifs of bZIPs

**Figure 5:**
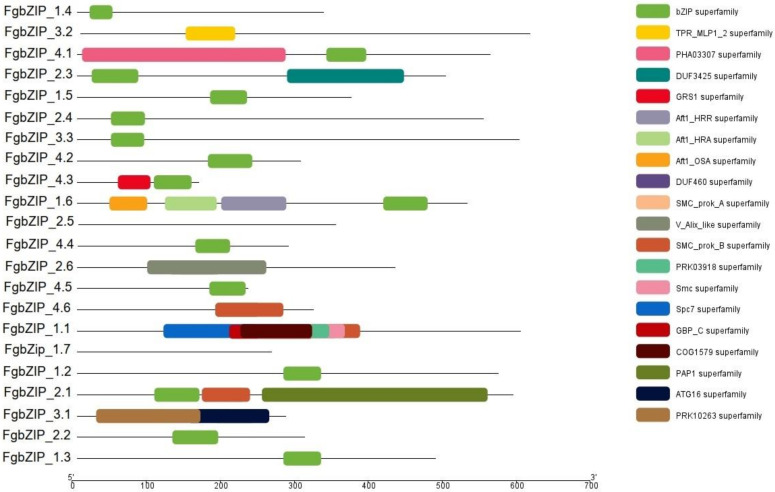
Conserved motif analysis of bZIP genes derived using Pfam figure showing conserved motifs highlighted in different colors, where bZIP is prominent in each gene.

The bZIP domain, which preferentially binds the ABREs cis-element is the core element of the regulatory region. However, additional bZIP protein domains may contribute to the bZIP functional diversity [50]. In addition to the bZIP superfamily domain, we also found 20 other functional motifs in *FgbZIPs*. These domains were presented with the help of TBTools. (Figure 5). Conserved Motifs were identified using the MEME Motif discovery analysis tool. Gene name, combined *p*-value, and motif locations were displayed (Figure S2); each color represents a specific motif and block length represents the length of the motif. It contributes to a better understanding of the bZIP gene family’s diversity and roles of the leucine zipper region. The amino acid range of these conserved motifs falls between 11 to 50. The first Motif was the basic region and the hinge of the bZIP domain which was conserved in all proteins. So other motifs including TPR_MLP_1_2, PHA03307, DUF3425, GRS1, Aft_HRR, and HRA superfamily motifs were also associated with bZIP motifs. In summary, the addition of these functional motifs provides additional features to FgbZIP proteins.

### 3.5. Protein–Protein Interaction Network and Subcellular Localization of bZIPs

**Figure 6:**
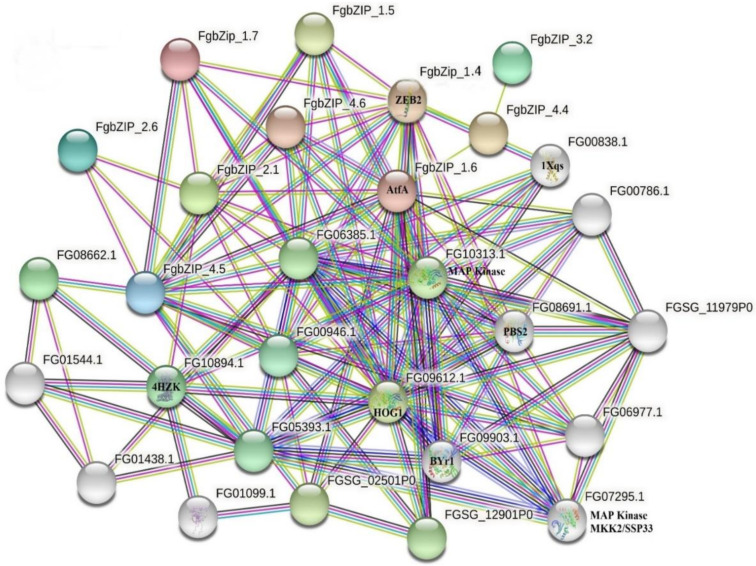
String network analysis of *FgbZIP* genes.

The protein–protein interaction network analysis demonstrated dimerization of bZIP within the family as well as outside the family. It is noticed that FgbZIP_1.4 is characterized as a ZEB2 protein that can interact with other proteins, such as ZEA1, ZEA2, and ZEB1 transcription factors, which are involved in the biosynthesis of zearalenone (ZEN) in *F. graminearum* [40,54]. Similarly, the FgbZIP_1.6 known as AtfA transcription regulator that regulates various stress responses, for example, conidial stresses interact with MAP Kinase which belongs to the protein kinase superfamily [55]. The bZIP protein also interacted with HOG1 whose pathway regulates hyphal growth, stress responses, and plant infection in Fusarium Species [56]. Likewise, FgbZIP TFs showed interactions with PBS2, BYr1, and serine/threonine-protein phosphatase 2B catalytic subunit. Meanwhile, FgbZIP TFs interacted with other proteins, which can be observed in the interaction network (Figure 6). Moreover, the prediction of subcellular localization showed that most of the bZIPs were localized in the nucleus. However, some were also localized in the cytoplasm and endoplasmic reticulum, for example, FgbZIP_3.3 was expressed in both cytoplasm and nucleus, and the FgbZIP_4.5 and FgbZIP_1.6 localized in the nucleus and endoplasmic reticulum.

### 3.6. Homology Modeling of bZIPs

The Swiss homology modeling approach was used to determine the three-dimensional structure of *FgbZIPs*. Because of the low accuracy of the SWISS model, we considered 11 models of FgbZIP proteins that had more than 30% homology. For instance, the FgbZIP_1.2 and FgbZIP_1.3 shared the same structure, 34.57% identical to 6iak (CREB3) protein in chicken which is redox-regulated and could be triggered in response to oxidative stress [57]. Similarly, the FgbZIP_1.4 and FgbZIP_1.5 are 39% identical to 2h7h uncharacterized protein found in the Aviansarcoma virus; the FgbZIP_2.1 and FgbZIP_2.4 were 50% and 33% identical to PAP1 protein involved in multidrug resistance, redox homeostasis, and oxidative stress responses respectively [58,59,60]. The FgbZIP_2.3 and FgbZIP_2.6 were 41% and 36% similar to the structure of 6iak.1.B, the FgbZIP_3.1 and FgbZIP_4.2 were 33% and 37% to 2h7h-AP-1 homodimer structure. According to the findings, most of the proteins lack β-sheets but contain α-helices. Aside from α -helices, the FgbZIPs consist of a few random coiled shapes. In the meantime, distinct members have varied numbers of α-helices and random coils, implying that different members of the same family may have diverse functions (Figure S3).

### 3.7. Expression Analysis of bZIP Genes in Various Developmental Stages and Conidiogenesis

**Figure 7:**
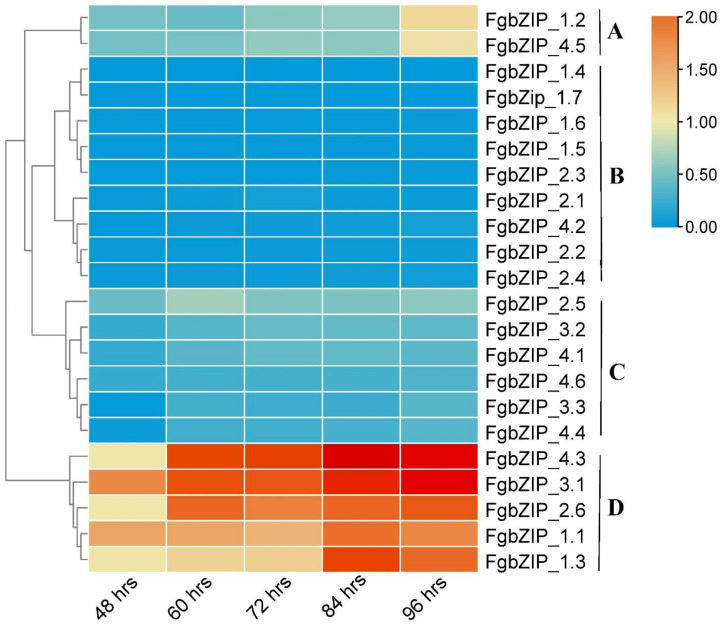
Relative expression pattern of *FgbZIP* genes under different stages of development. Relative expression values of RT-qPCR results were transformed by log2. Light and dark colored boxes reveal lower and higher expression levels, respectively according to the scale. The alphabets A–D on the right depicts grouping based on the expression of genes.

All members of the *FgbZIP* gene family were examined during different time intervals of the growth of the fungus. As demonstrated (Figure 7), the *FgbZIP* gene family has a comprehensive expression pattern during five mycelial developmental stages that are at 48, 60, 72, 84 and 96 h. The *FgbZIP_1.1*, *FgbZIP_1.3*, *FgbZIP_2.6 FgbZIP_3.1* and *FgbZIP_4.3*, were abundantly and constantly expressed in all examined stages. While *FgbZIP_1.2*, *FgbZIP_2.5*, *FgbZIP_3.2*, *FgbZIP_4.1 FgbZIP_4.5* and *FgbZIP_4.6* moderately showed their expression. In contrast *FgbZIP_1.4*, *FgbZIP_1.5*, *FgbZIP_1.6*, *FgbZIP_1.7*, *FgbZIP_2.1*, *FgbZIP_2.2*, *FgbZIP_2.3*, *FgbZIP_2.4*, *FgbZIP_3.3*, *FgbZIP_4.2* and *FgbZIP_4.4* were expressed at relatively low levels. The co-expression heat map and phylogram was divided into four groups (Figure 7). The D group had relatively high expression to other groups, followed by Group A (only two genes), B (nine genes) and C (six genes). Group A, C and D formed 60% of the total genes and showed relatively low expression while D group had high expression in all five stages (at 48, 60, 72, 84 and 96 h) of growth and development. Overall, the expression levels of *FgZIP* genes were enhanced with the growth time (Figure S4). Among these 22 *FgbZIPs*, three genes (*FgbZIP_1.4*, *FgbZIP_1.2* and *FgbZIP_2.1*) have already been studied with almost the same expression pattern [40,41].

### 3.8. Expression Patterns of bZIP Genes in Response to Abiotic Stress

**Figure 8:**
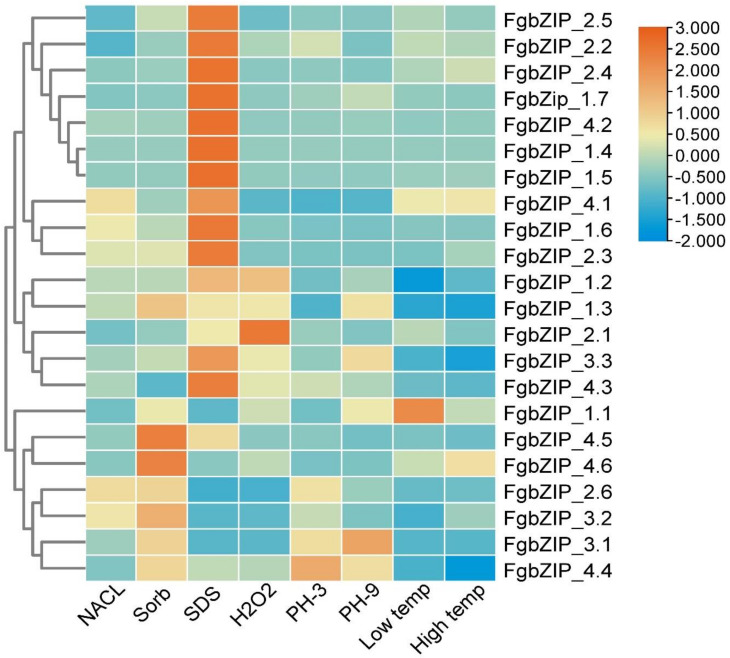
Expression profiles of *FgbZIP* genes response to abiotic stress. Relative expression values of RT_qPCR results were transformed by log2. Fold changes in gene expression are shown in color according to the scale. NaCl: sodium chloride, Sorb: Sorbitol, SDS: sodium dodecyl sulfate, H_2_O_2_: hydrogen peroxide, Low Temp: low temperature (4 °C), High Temp: high temperature (37 °C).

The bZIP TFs are involved in essential bioprocesses in a diverse range of filamentous fungal species. The bZIP proteins control many important biological functions categorized as developmental, amino acid synthesis, nutrient utilization, unfolded protein response (UPR) and several stress responses [61]. To examine the expression pattern of the *FgbZIP* genes to various abiotic stresses, a quantitative RT-qPCR was performed after 5 hrs of exposure to stresses. Results demonstrated remarkable changes in the transcript level of *FgbZIP* gene under different stresses. In response to ionic or NaCl stress (1 mol/L), eight *FgbZIPs* (*FgbZIP_1.1*, *FgbZIP_1.6*, *FgbZIP_2.3*, *FgbZIP_2.4*, *FgbZIP_4.1*, *FgbZIP_4.2*, *FgbZIP_4.3* and *FgbZIP_4.6*) were upregulated, while fourteen *FgbZIP* genes (*FgbZIP_1.2*, *FgbZIP_1.3*, *FgbZIP_1.4*, *FgbZIP_1.5*, *FgbZIP_1.7*, *FgbZIP_2.1*, *FgbZIP_2.2*, *FgbZIP_2.5*, *FgbZIP_2.6*, *FgbZIP_3.1*, *FgbZIP_3.2*, *FgbZIP_ 3.3*, *FgbZIP_4.4* and *FgbZIP_4.5*) were downregulated. Similarly, under osmotic stress (sorbitol) treatment fourteen genes (*FgbZIP_1.1*, *FgbZIP_1.3*, *FgbZIP_1.6*, *FgbZIP_2.1*, *FgbZIP_2.2*, *FgbZIP_2.3*, *FgbZIP_2.4*, *FgbZIP_3.1*, *FgbZIP_3.3*, *FgbZIP_4.1*, *FgbZIP_4.2*, *FgbZIP_4.4*, *FgbZIP_4.5* and *FgbZIP_4.6*) showed upregulation pattern, whereas eight *FgbZIPs* (*FgbZIP_1.2*, *FgbZIP_1.4*, *FgbZIP_1.5*, *FgbZIP_1.7*, *FgbZIP_2.5*, *FgbZIP_2.6*, *FgbZIP_3.2* and *FgbZIP_4.3*) were downregulated. As a result of cell wall stress (SDS) seventeen *FgbZIP* genes (*FgbZIP_1.1*, *FgbZIP_1.2*, *FgbZIP_1.4*, *FgbZIP_1.5*, *FgbZIP_1.6*, *FgbZIP_1.7*, *FgbZIP_2.1*, *FgbZIP_2.2*, *FgbZIP_2.3*, *FgbZIP_2.4*, *FgbZIP_2.5*, *FgbZIP_3.3*, *FgbZIP_4.1*, *FgbZIP_4.2*, *FgbZIP_4.3*, *FgbZIP_4.5* and *FgbZIP_4.6*) were observed upregulated, While remaining nine *FgbZIPs* (*FgbZIP_1.3*, *FgbZIP_2.6*, *FgbZIP_3.1*, *FgbZIP_3.2* and *FgbZIP_4.4*) showed downregulation pattern. Under redox treatment (H_2_O_2_) nine *FgbZIPs* (*FgbZIP_1.1*, *FgbZIP_1.2*, *FgbZIP_2.1*, *FgbZIP_2.2*, *FgbZIP_2.3*, *FgbZIP_2.4*, *FgbZIP_3.3*, *FgbZIP_4.3* and *FgbZIP_4.6*) were upregulated, whereas rest of other *FgbZIPs* are observed downregulated. Due to acidic stress (PH-3), nine *FgbZIPs* (*FgbZIP_1.1*, *FgbZIP_1.7*, *FgbZIP_2.1*, *FgbZIP_2.2*, *FgbZIP_2.4*, *FgbZIP_ 3.1*, *FgbZIP_4.3*, *FgbZIP_4.4* and *FgbZIP_4.6*) were overexpressed, while rest of *FgbZIPs* were observed suppressed. At the other hand *FgbZIPs* (*FgbZIP_1.1*, *FgbZIP_1.7*, *FgbZIP_2.1*, *FgbZIP_2.2*, *FgbZIP_2.3*, *FgbZIP_2.4*, *FgbZIP_3.1*, *FgbZIP_3.3 FgbZIP_4.2*, *FgbZIP_4.3*, *FgbZIP_4.4* and *FgbZIP_4.6*) are upregulated, while remaining *FgbZIPs* were downregulated at high PH-9. Under low temperature (4 °C), seven *FgbZIPs* (*FgbZip_1.1*, *FgbZIP_2.1*, *FgbZIP_2.2*, *FgbZIP_2.3*, *FgbZIP_ 2.4*, *FgbZIP_4.1* and *FgbZIP_4.6*) were over expressed, whereas rest of other genes under study are suppressed. Under high temperature (37 °C), the genes which are up-regulated are (*FgbZIP_1.1*, *FgbZIP_2.1*, *FgbZIP_2.2*, *FgbZIP_2.3*, *FgbZIP_2.4*, *FgbZIP_4.1 and FgbZIP_4.6*), while *FgbZIPs* (*FgbZip_1.2*, *FgbZIP_1.3*, *FgbZIP_1.4*, *FgbZIP_1.5*, *FgbZIP_1.6*, *FgbZIP_1.7*, *FgbZIP_2.5*, *FgbZIP_2.6*, *FgbZIP_3.1*, *FgbZIP_3.2*, *FgbZIP_3.3*, *FgbZIP_4.2*, *FgbZIP_4.3*, *FgbZIP_4.4* and *FgbZIP_4.5*) were down-regulated. An overall upregulation and downregulation pattern of the genes are presented in the form of heatmap (Figure 8 and Figure S5).

### 3.9. Circos and Synteny Analysis

**Figure 9:**
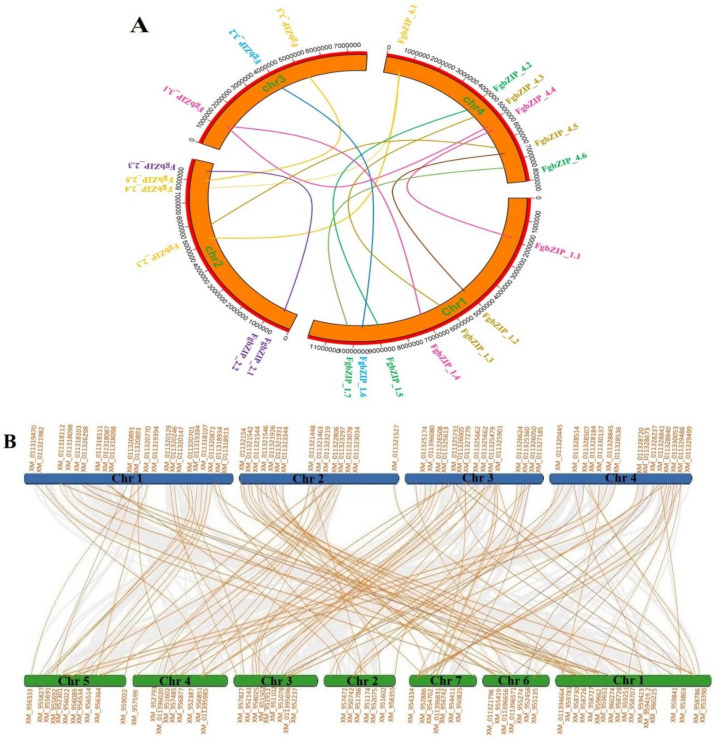
(**A**) Synteny analysis and chromosome localizations of F. graminearum (FgbZIP) genes (Paralogs). Block regions with orange color represent Fg chromosomes and genes indicated on a scale of bp paralogs colors and line pattern is according to phylogenetic trees clades displayed during gene structure analysis. (**B**) Synteny analysis (Orthologs) between *F. graminearum* whose chromosomes are indicated with blue color block and *N. crassa* whose chromosomes are indicated with green blocks, Supplementary File S5.

The gene locations and paralog pairs are studied and generated using the advanced circos program in TBtools. The 22 FgbZIP genes were randomly distributed on the 4 chromosomes of F. graminearum, with both separate and bunched patterns. Two or more genes when present within a 200 kb region on chromosome is characterized as a tandem duplication [62], *Fg* Chromosomes displayed 6 tandem duplication events on chromosomes 1, 2 and 4. Segmental duplications are duplication events that result in genes that are found on separate chromosomes [63], which are symbolized by colored lines, for example, *FgbZIP_1.5*/*FgbZIP_4.2* and *FgbZIP_2.3*/*FgbZIP_4.1*. We found 9 segmental duplication events (Figure 9A). Genes that have undergone tandem and segmental duplication events have a closer genetic link, and these findings could be used to predict functional outcomes.

The relationship between the *F. graminearum* and *Neurospora crassa* orthologous gene pairs were identified using MC Scan X and Dual Systeny plot programs (Figure 9B). The synteny analysis demonstrated gene duplication events based on whole-genome analysis. Orthologs among both species and their respective chromosome locations file is attached in Supplementary Data (File S5).

## 4. Discussion

The ascomycete fungal pathogen *F. graminearum* causes FHB, which leads to serious qualitative and quantitative losses to cereal crops [61]. The hazardous secretory toxin of this fungus, particularly deoxynivalenol (DON), poses serious health concerns to both humans and animals [64], as well as indirect costs such as grain rejection or downgrading at marketing due to contamination by mycotoxins [65]. Transcription factors are important elements to control diverse key cellular activities. They are key components of the signal transduction pathway and serve as a vital link between signal flow and target gene expression [66]. bZIP TFs especially are the most diverse TF family [67]. So far, the *FgbZIP* gene family has not been reported on a genome-wide scale. Thus, the identification and investigation of *FgbZIP* TFs could help us to understand their role in various biological mechanisms and could enable future research. Studying gene families has become increasingly viable due to the quick advancement of high throughput sequencing technology and its data availability [68,69]. The bZIPs are widespread TF’s having critical roles in growth, biological processes, and responses to various stresses in plant and fungal kingdoms [29,70,71].

The bZIP TF’s have been discovered and characterized in numerous plant species. However, fewer studies exist regarding the bZIP gene family in fungus, although a significant number of unique bZIP genes with specialized functions have been discovered [71,72,73]. Previous studies disclosed a higher number of bZIP TFs in plants; there were 247 bZIP genes in *Brassica napus*, 119 in wild cabbage, 138 in soybean and 125 bZIP genes in maize [74,75,76,77]. While comparatively fewer bZIPs were found in organisms belonging to kingdom Animalia—56 in *Homo sapiens* [78], 27 in *Drosophila melanogaster* [79], and 31 in *Caenorhabditis elegans* [80]. Whereas, there is a consistently lower number of bZIP genes in fungi—34 bZIP genes in *C. minitans* [37], 17 in *S. cerevisiae*, 22 bZIPs in *M. oryzae* [39], and 28 bZIPs in *U. virens* [38]. Our study identified 22 bZIPs in *F. graminearum* correspondence with other genome-wide studies in fungal species. The lower number of bZIPs in fungi may be due to the size of the genome as compared to plants. In addition, the bioinformatics analysis, including gene mapping, intron-exon distribution, gene features, and proteins features, provided detailed information regarding *FgbZIPs*, which will be helpful for further functional study.

Since the lifestyle of fungi heavily depends on their adaptations to their environment, replicated genes may lead to new functions and improve adaptations in a changing environment. In pathogenic fungi, improved nutrient uptake and more efficient catabolism, resistance, and adaptation to host infection can also be obtained through the expansion of gene families [64]. The bZIP superfamily is reported to have evolved from a single eukaryotic gene ancestor and has experienced multiple independent expansions [60]. Our phylogenetic analysis of FgbZIP with other species bZIPs showed that they were evenly distributed into nine (A-I) groups showing their close sequence similarity. In addition, the evolutionary analysis of eight species also demonstrated several gene duplication events in the phylogenetic tree nodes indicating bZIPz extension with the passage of time. As it is also reported that the bZIP superfamily is developed from a single ancestral eukaryotic gene and has gone through numerous gene duplication and family expansions [81].

In eukaryotes, including fungi, protein phosphorylation is a prevalent type of post-translational modification. It is a reversible change that regulates a variety of cellular activities, including metabolism, transcription, cell cycle, filamentation, mating, cell wall synthesis, cellular maintenance in stressful and virulence conditions. Threonine (Thr), Serine (Ser), and tyrosine (Tyr) residues elucidate more than a third of all protein phosphorylation events [82,83]. Following the statement, around 70% of the *FgbZIPs* studied in this work contain 20 or more phosphorylation sites indicating rich diversity of post-translational modifications. Except for a few instances where *Tyr* phosphorylation sites are higher than *Thr* sites, ser phosphorylation sites are consistently greater than *Thr* and *Tyr* phosphorylation sites. Such a study was conducted by (Yang et al., 2019) where the number of predicted phosphorylation sites were four (minimum) to 28 (maximum) in *Ipomoea trifida* bZIP proteins and demonstrated that 60% of the identified bZIPs had 10 or more phosphorylation sites including 80% of Ser phosphorylation sites and 20% *Thr* phosphorylation sites [84]. Thus, our predicted phosphorylation sites were similar to the previous studies, indicating the highly conserved patterns of bZIPs in fungal species. The cellular localization analysis predicted the nucleus as major localization organelles, which may be due the presence of nuclear signaling peptide in the bZIPs proteins. The sequence The standard bZIP domain holds a highly conserved basic motif region and a varying leucine-zipper region or several bulky hydrophobic heptad repeats of other bulky amino acids [85]. Almost all the bZIP members hold the conventional conserved pattern of invariant residues (N-X7-R/K) and the leucine-zipper region. Some exemptions were observed in a few FgbZIP genes, for example, in 1 out of 22 genes (4.54%) in the basic zipper region the core asparagine (N) residue was replaced by isoleucine (I), such a relevant pattern is also observed in poplar [86]. In the invariant N-X7-R motif’s Asparagine (N) and arginine (R) should be the most conservative position in the bZIP domain; only two genes, *FgbZIP_1.6* and *FgbZIP_3.3* have mutations at these points.

Because of the large number of family members, the *FgbZIP* genes must have functional termination. As a result, certain genes may drop some amino acids because of evolution. Phylogenetic analysis from 135 full-length sequences distributed in 9 groups (A-I) demonstrating the existence of the bZIP gene family before these fungi diverged and genes do not demonstrate a great variation among the genomes of different fungal species. Four FgbZIP proteins in group F were more closely related than other bZIPs, implying that a genome duplication event occurred in recent times. While the number of bZIP proteins in S. cerevisiae are more than that of other species, suggesting an evolutionarily significant expansion event. The evolutionary analysis of bZIP in eight species which consist of 135 genes revealed information about genome evolution, gene duplication, ortholog duplication, and the phylogenetic tree of the species. All these genes were classified into 18 orthogroups. Each orthogroup has a distinct number of genes in it. Surprisingly, just three orthogroups were shared by all species. As a result, some orthogroups were specific to clades, whereas others were restricted to sister taxa. The species-wise evolution also provided detailed evolutionary relationships among eight species where the species phylogenetic tree was divided into two major clades. In such a genome comparison study of an edible mushroom (*Stroharia rugosoannulata*) with cluster of 16 fungal species, out of the 16,680 groups studied, 7766 (46.6 percent) had at least one *S. rugosoannulata* gene. In *S. rugosoannulata*, there were 197 species-specific orthogroups containing 881 species-specific genes. The phylogenetic tree was built using 647 single-copy orthologous genes from 17 different fungus. By using ortholog-based clustering analysis, it is predicted that *S. rugosoannulata* belongs to *Strophariaceae* family, based on physical features and NCBI taxonomy [87].

Gene structure characteristics have been visualized where we observed some loses and gains of exon and introns in the gene structure. It has been observed that introns are lost faster than they are gained after segmental replication [65], indicating that a putative gene duplication event happened in a recent period in *F. graminearum.* Similar results were found in the bZIP domains of *C. minitans* and *U. virens* [23,25]. In eukaryotes, the evolution of introns may have a role in the functional evolution of paralogs, and more introns usually imply more complex regulation [58,59]. Thus, the lost and gained introns in FgbZIPs may have a putative role in metabolic regulations.

The bZIP proteins are found worthy for essential cellular decision-making pathways and individual bZIP proteins may perform different but related biological activities [88]. Three dimensional (3D) structural homology modeling of FgbZIP amino acid sequences was analyzed, where proteins mostly lack β-sheets but contain α-helices. At the same time, distinct members have varied numbers of α-helices and random coils, indicating that different members of the same family may have diverse functions. Protein–protein interactions showed that FgbZIPs interacting with various other genes as discussed in results like FgbZIP_1.4 (*ZEB2*) encode a transcription factor that controls the expression of other members of the zearalenone ZEN biosynthesis pathway gene cluster in *F. graminearum* that interacts with FgbZIP_1.6 (*atfA*) that is a stress response regulator in *Penicillium marneffei* and *Aspergillus nidulans* [89,90]. According to our expression pattern data, individual bZIP proteins displayed various expression patterns in different situations, indicating that they may have different functions. In *F. pseudo graminearum*, bZIP TF *FpAda1* is important for fungal development and conidiation [29], while *YAP1* is required for oxidative stress tolerance in A. fumigatus [19]. Overall, in our study, seven *FgbZIP* genes were expressed relatively highly, six *FgbZIPs* were moderately expressed, while nine *FgbZIP* genes showed relatively low expression during developmental stages. In the later phases of development, the majority of *FgbZIP* genes were overexpressed (Figure 7). Almost such a pattern of overexpression of most of the bZIP genes during later days of growth can also be observed in *Coniothyrium minitans* [37]. This gives a hint that some *FgbZIPs* were maybe responsible for the expansion of the fungus. Low temperature stress suppressed the expression of most FgbZIPs, but the induced expression of FgbZIP_1.1. During abiotic stresses, the expression of some of the genes were noticeably uniformly high such as *FgbZIP_1.1*, *FgbZIP_3.1* and *FgbZIP_4.3*) (Figure 8). Redox stress (H_2_O_2_) repressed most of the *FgbZIP’s* while induced expression of certain *FgbZIPs* (FgbZIP_1.3, *FgbZIP_1.1*, *FgbZIP_1.2*, *FgbZIP_2.1*, *FgbZIP_2.2*, *FgbZIP_2.3*, *FgbZIP_2.4 FgbZIP_3.1* and *FgbZIP _4.3*). Likewise, in another study, when *Ustilaginoidea virens* was exposed to H_2_O_2_ stress, eight bZPs had significantly higher expression levels at 1 h after treatment compared to controls. The expression levels of seven *UvbZIP* genes observed suppressed or very low expression [38], while during cell wall stress (SDS) most genes were observed to be upregulated, while, relatively, downregulations were observed for five *FgbZIPs*. At low pH (PH-3) stress most genes were down-regulated, but *FgbZIP_3.1* and *FgbZIP _4.3* were up-regulated (Supplementary File S4). It is suggested that these *FgbZIPs* are thought to be involved in the delicate regulation of biological activities or have genetic redundancy of genes. Therefore, more functional studies of individual FgbZIP genes are needed to gain a better understanding of this potent pathogen.

In conclusion, we thoroughly identified and studied 22 *FgbZIP* genes in *F. graminearum* on a genome-wide scale and investigated their expression behavior during various developmental stages and in response to different abiotic stresses. During different mycelial expansion stages, the expression of five genes was high constantly, eight genes were moderately expressed, while nine members showed relatively low expressions. In response to various stress conditions, different patterned regulations were observed, for example, during cell wall stress, maximum genes (77%) were upregulated, whereas in response to ionic and redox stress almost 60% of the *FgbZIPs* were downregulated. The genome-wide systematic characterization and expressional analysis of *FgbZIP* genes reveals the molecular activities, such as development, metabolism, physiological functions, and stress responses in *Fg* and served as a reference for other plant pathogenic fungi. This study would be useful in selecting candidate genes to investigate the function of genes by knocking them out.

## Figures and Tables

**Table 1:** Characteristics of bZIP gene family in *F. graminearum*.

Gene Name	Gene ID	Length (nt)	MW(Da)	PI	Subcellular Localization	Chr No.	No. of Phosphorylation Sites			
							Ser	Thr	Tyr	Total
FgbZIP_1.1	23548003	1901	66620.22	4.970	Nucleus	1	24	5	3	32
FgbZIP_1.2	23548989	2295	62349.02	4.490	Nucleus	1	26	9	2	37
FgbZIP_1.3	23549271	786	53346.27	4.610	Nucleus	1	22	7	2	31
FgbZIP_1.4	23549776	1048	37664.64	5.750	Nucleus	1	20	2	4	26
FgbZIP_1.5	23550109	1113	40664.44	7.150	Nucleus	1	22	6	2	30
FgbZIP_1.6	23557062	2519	55444.4	9.010	ER, Nucleus	1	28	12	3	43
FgbZip_1.7	23560565	788	28795.86	5.730	Nucleus	1	14	5	3	22
FgbZIP_2.1	23555786	2456	63564.24	4.950	Nucleus	2	44	9	4	57
FgbZIP_2.2	23560120	1260	33990.35	6.670	Nucleus	2	14	2	2	18
FgbZIP_2.3	23559160	1655	55819.27	5.780	Nucleus	2	20	3	6	29
FgbZIP_2.4	23550377	1737	61730.63	5.010	Nucleus	2	30	8	8	46
FgbZIP_2.5	23550286	1279	38592.17	5.710	Nucleus	2	15	7	2	24
FgbZIP_2.6	23558445	1318	46629.52	4.520	Nucleus	2	33	10	1	44
FgbZIP_3.1	23552363	2372	31208.33	5.330	Nucleus	3	17	3	2	22
FgbZIP_3.2	23553081	2218	65908.15	8.380	Nucleus	3	40	17	3	60
FgbZIP_3.3	23553554	1910	66021.86	5.230	Cyt, Nucleus	3	30	20	3	53
FgbZIP_4.1	23553774	1674	60396.63	9.960	Nucleus	4	43	12	5	60
FgbZIP_4.2	23554841	1017	33766.39	5.750	Nucleus	4	16	10	3	29
FgbZIP_4.3	23554911	1280	18012.13	9.040	Nucleus	4	22	8	0	30
FgbZIP_4.4	23556762	1299	30497.77	6.670	Nucleus	4	10	2	1	13
FgbZIP_4.5	23556246	1582	24980.68	4.730	ER, Nucleus	4	18	1	2	21
FgbZIP_4.6	23555970	1008	35630.37	6.020	Nucleus	4	25	2	1	28

## Supplementary Materials

The following are available online at https://www.mdpi.com/article/10.3390/genes13040607/s1, File S1: Phylogenetic Sequences, File S2: Orthologs analysis, File S3: Primers, File S4: Expression Data, File S5: Synteny data, Figure S1: Chromosomal Mapping, Figure S2: Motif analysis, Figure S3: Homology modeling, Figure S4: Expression in developmental stages, Figure S5: Abiotic stress expressions.
