# Supplementary material for: Genome-Wide Identification and Expression Analysis of the Basic Leucine Zipper (bZIP) Transcription Factor Gene Family in Fusarium graminearum
Source: Genes (Basel). 2022 Mar 28;13(4):607. doi: 10.3390/genes13040607 (PMC9028111; doi:10.3390/genes13040607)
Supplement: Supplementary file 1 [file genes-13-00607-s001.zip › Supplementary Files/File S1 Phylogenetic Sequences.pdf]

>FgbZIP_1.1

MSSTFQMQQPFYRPASLTLDTQHAQKYFEDEDNSVLDDNVLDHNTLDSGLELSPPMADSRRDSFAVGHPLF
SPKTEDWQSVDMQSVPSNNPFLDQHSNNPFMRLDQTQHNAYATANWAFGPSSGACTPLHPFDAMSAEY
DTNVPLFQRPMAGPTPFTNPGNMFPALPGGNQSIPTSPQKEWMTPQQPINKNKMRPGSPGIRSHNEMRR
GDGIRKKNARFDIPAERNLSNIDHLISQSTDEQEIKELKQQKRLLRNRQAALDSRQRKKQHTERLEDEKKQYTA
VINDMEEEMAELRGKLEQMLLEKQTYTNYIENLNLEKEEMIRAHTIETGELRKKVGVLTDHVQRLESAVPTAT
DNGFSSGFDDMDGMGMPGAWDASNFLNEYPAEPEVKQEMAIATVKKSENPFAESEKPAQGGLLFMLFLV
GAFVLSSRSTPAIPRVSEDVRVASANILDSVLKDAGVNSQSATLQAPPQPSGAANWGNPIAMNDMMMDG
VAPSMLSELGDTLTQPTQEQTNEQIFSLSAAQYNGVNSQDFLQNAPEKSSPSQGRRNLAEALAAMRSNKQS
DAADVYTRSLLWDQIPNDVVRNFAKMVSECNSAE

>FgbZIP_1.2

MSAPGQNNVDFDALLDLTEYDGFQSPASLSPAGTSKATFTSPVTAAVAAPVTTAQSLSGPSHNYDMYRQQT
GFVPGAIASTMAVNQTNNTGYQDFRSLDYSTFSPEADLFDFNTSPSQATMGASEMDMDFESHTETQQFFTV
DPSSIEQEVDGLPSPPVLPTQTNNVGRLWPGAHSQAALAKAQAQQRQQQQIIQQQQQAQRQGSQPKSRG
KAPPPSDPLVEQKITQLLNSMRAKPSMPDSQATSPMTNLPRSKKDEEEMDEDERLLASEEGKKLSSKERRQLR
NKVSARAFRSRRKEYITQLETEIANKVSENGDLRTQNRALLDENKRLTDLTRMLLSSPSFSNFLDNLSSNPAAA
QQTPQLKVEPQPEQRQVPKDINPYNAQQSSQQQIGMAMIPEQNMDFSMLTLDGFNFQPQVFVVDTPEVP
EIIDAAVLSGKSSNFVEPIFDSEEEKLEVPAIERPVAAPEVSEPVNAAPIDAEFESDPEFALFHTEAATTATESPEE
FDSEGLSHVDIFGGVESEKVLARLELVDAGEQECTAALAMARVQRLSASCDAVTSRLELLTMDL

>FgbZIP_1.4

MQSTESFHALPTRSDVEDPNERRKIQNRIAQKKHRQKMKRRIEELETKVNNQCQTSNWTSHAPTDSCQEQQ
FLDNTDFGLMLEDDLLYRELSASLDGAGLTAVAQMHDSPRPNQQQRLSVSGMPSSPTSTSNVAQRGLSIGD
HSSASNHLSSLSLVPGSTEGSLPTRQHDNLCNQDLRDMVPEEKMSRILKVIQDAGYKDMDSFMTEYYVRDFD
ASSHVSAVQRQSRSRRLRGFLEQLRVGAESWSDYEAHDYQQEISKSAEAIYAKELDVFSTTTLGENASFQGLAS
LYRVLQSTVGSDIENHLRHEQSMMQRQVSEIVHRQVYTFNPAQC

>FgbZIP_1.5

MDIPFGNPMNIMPTTPSPGYTMTSSSCITRTPSPASRGNIGYQPYPGQAQGPSFFEEAIGGSPFPQEIHFSLIQ
PPHYNSTSGIPNSFPPSSATMPSSEAEQKSTCRPRSRNPHPLSVNTSGQVNGPDVTAKSPKPKRRGRKPKGPP
KDAVKCREDIELDEDDLPKDPRRRRILERNRIAATKCRLRKRDEASALASQEQAMEDQNRYLSSCFDSLTAEIY
HLKTQLLQHTDCNCVLIQKYISNEAKKTVDGLSDCPSNFQPDSDAMSPYRRGSCHSGTSPTESLSVPTPGFEG
ASPGWPQRFHSGHGSSPDVTEEMLAVNPYLKASMQMGSQSFANMAPVPHHGLEVFACMGPQHQEFEAV
NWNPSWAF

>FgbZIP_2.5

MSQSKSPNFLGVMSSEASKSRRERNREAQQQFRRRRQAVETARLQRLKHLEGIIERMSTVIVSFTDQMLHED
VLKQYPVLAANAQEVITHVLALAHEAEDADEGTTTEAASPNGRYEFSAAQDSGFSELSFHSGPMQAKMMNA
NQQSAVFMTHPSSQYPQAMGDPTIAYTNLQDPTLSELPSLPPSTLLPSLGPVPWNTSKPLSPTSFTYRLTHSCF
NDERESMTRKLRWITGIGSQDIKLASILPWGGRYCGEDFTGDDLSSTCKTTDRTALQFLSAAAQDTLELDLSGA
MGNDDPRPLQPDSWSFVNFFPPGIIAQKGTSAKIRCVFSDGDWVSKERAQKGNYGLQG

>FgbZIP_2.4

MIPLYVPESSSGESSRVEDGGDAPPAPRRGRRPVETPVVGSVSNRRRMQNRMAQRAYRQRKESAIDVLKKK
VEELEKVKEDIGNEFLSLTSVILEKEGVGDCPDIVEHIKKSTINILTSTREVDETSETEDHDMNPVPSTSEAPAGSS
TSYTLTTDQVNSLGFPMVDYVPELEFDNMFDQNIDAQPTTQYYDYNHSTPSTHDRGSYFQPSFYQTPGYNIS
PLPGATYDTSLSCPRSYSSHEATFGRRYHRATQEAAFLLASMKHSPPTWYQKVFGFCLHFETRDEITQRIGDCL
RKPREATLNNWKFPFTNVGGAGLFYPNQGYDGSELPIGNRSLSNETFRPSELSGFSMGPFGPTIEQVRDLRLS
PQLRIIDPNYDGDFFDADEIEISLRGYGVIIPASKDYVTAYIDMAMFERAEARESSGSDFEDPVTPPDISTDTGN
DFEDASVSYDTEAAPLHCPVGLMGLATGEMPPYPKSQPQEQWKTPTRRETRVKVDVEKLIMCKLKTHMSLK
LANAFDRFNQFYCLLRTDSCRSSERYKDGIENVSH

>FgbZIP_3.1

MSNFNGRRGPNVSQYLRDLNAINRQENAHDEPFNMEEDLALFTNTQFFDFETGQNTDYQAHPAKVDLEAS
QSTSPSDGMTPAPSVVGDIAAGNFDFMQGDFNFPDFAGPYPSTPMTAFADGAQNFAPLQPNAPTNYQPV
PQQRQAPQFAQPAAPQPSLEKRNSESAGSSGRGSLNFEEASRHAAEEDKRRRNTAASARFRIKKKQREQALE
KSAKEMSEKVSVLESKVSQLETENKWLKNLLVDKNEGNDEIIALWKEFAASKTANKPELKAKSSVKDETR

>FgbZIP_3.2

MSASSPQQSIESPGAATPSIAPSPAGTRSRAISAAPGPASPATTGSHSHRTPLSIKPSSKPPPPTQPHQRTALPSI
NKMAMSSVISPSPAPPEPPKVSMTSKEWVIPPRPKPGRKPATDTPPTKRKAQNRAAQRAFRERRAARVGELE
EQLDQQREVQEKHESDLKDKIHELELDVQSYRSRCMLLENMLERERQDRIRVETEAETLKRRLDDGIFNSNFQ
SRTMSAQHSFDGMHSPTSQGPRHSLPDGRPDRQSGHSFSISQIISPPETLDMNSSHDPETTLTCGNCSPNGH
CACAEEVMRTAESGCGKCSLSSTCQCLDEAAEALDRAQELKRPVSPSADVSHEKRHRSSPHDTVETDYTAMF
SRKPPQETFSVPTQLPSMDPMPFRDGCGFCKDGTYCVCADTALATPAMTPNDTLPPISQQVQTPPPEETDLP
ILAMEMTADGAVKLPRRTQSKPTERSSGCGPKGPGSCAQCQADPKSGLFCRLMAANFNRKDGSSGGCCGG
KGAGGGCCKSQPKQPQQAQQPEKINLPSLPSLGLSCAEAYQTLSSHRNFSKAADDIGSWLPKLKATPRPGTRP
TPPGSMMPIEVEAASIMSVLKDFDVRFGRGI

>FgbZIP_3.3

MLEAPQTEPESLTERKRARVSEDNEEDTGKKRSRGRPRLDTRDETAQDRRRTQIRLAQRAYRNRKDTAITTLE
DKVKDLEDANENMSKEFMNFFDFVLSQGMLQGAPEVARRLNDTTRKFLSLTRKSAEDSSRDESGGAPAPTP
MQGEDATQPPERRISAHSSNTSSSPNDNLSAPNPSVPRMIPQDKQQTTPQRVNGQMRQQATPPLNLPFEII
TMPTTDNASFPVYDTQTPINLEQNPFLQSPFPGVPSPPSYSSQERSFGRRLQRASLEAGLRLASMTNPPPHRY
AQVFGFCLLFEPKESIVNRMSTTLSRISQESLFVWRYPFTNLGGAGTFFPDNEGAGGYSSTVPNGNRLPLGNQ
GLVQSMKPPEMTGFSMGPFGPDVEITRDRVDERMRMMFKGFEGDFFDADEAEMYLRQKGIVIPANVDFID
AEIDIGSLGESPDLSNFGVNNNSFFGAPQASDTNQGLYIPPQQSARDVPGMWQSPVSTSVASGTSSMTAASL
MAPTTGPDMASFMPQLGPTNGEQYGQGLASFMDTSYLPREWTTNPSWMKTKVTVDVNRLVAEMTSMA
VCLGRTPAVRPQDIDKAVKLAIVLTQS

>FgbZIP_4.1

MSHTEPASQHAPGRSSRSPMGEALVSPTSTRHAHLGDRDLRHDGSNTPYSNGEERMQQVPQKTLGVHNIL
NPLEPRPSASGGNGPMHPAARPSDSAMHPQTPGSIHSPFHTTRSFPHGQNASISLPGTPVGSMTPVGAPTS
GRNSPITGYPFPSVGNTRQRASPTQHSRAISVSHVPSRESDGRQPSSLHGKRPYEEMAAEDTRSHYPNIHHLS
GMPAGPPSTMSDSGRLHSQGPMSSPGLQAPHSGLPPNHMTSRQQPPTVHHQTSYSPNMQGGRPFPSPG
PQNENNTLWSETLRRQGTGGSLFGVEGQQAFMTLPGNETPIPVHMDFSHASKKADEKRQRNAVASTRHRR
KKKIMQEENSKQLQELRDERRLMEIRIEELTQQRDFYRDDRNRLRDIVGQTPSISGLAAGPPSPTFSVSNSYAE

TGSMVSGHMGYGGEGMSNERPSQRRRTDDHPEYSLPPYSPASVTSGHPSASPSQLPPMPMPGYGGPSRPSSAAS

SANSERLPPLRAMDGRSLGPPPGSGVQEQDMRTGQWVPVQPRAQETGWATRDTHRRP

>FgbZIP_4.2

MEYSEWLEHGWMPSISGYLESNDYLSDYCPIDDNLPGLSIYQTQEDWNPFPSPPYPTEEDSPESAWLADNGQGWHEVSAFADTCIDRQHVESTPSGSTGSRPSTQTSARLSKIHLPSAGVSTGDIIGQPHDEHKHEELADLSTGKGRTKRKCLSEGTKDTVRSRGASSRSTVKANRQQRVMEQNRLAASRFRKRKRSTADALGMDLERLEDQHRELSKHRNGLQDELFKLKSQVLEHGSCDCALMRQYIDNEAHRIVLESSLSTWTTIELPPILHLVLDTVTWKSTSCATNGAALRTDKSI

>FgbZIP_4.3

MIPELNVLDTQSSLDVVGDGFLSQEQPLLTVNSTTPIEYPATASSSSSTPHPPPTIRTKSPTSTSAIPGTSTFSLHPSPSSSSSSSSSSSRKRKSSPEEEDSAVTLKRQRNTLAARKYRQKRIDRITELEEALAAKAKECDDWKLKFMRKEAENDTLREMLAKK

>FgbZIP_2.1

MASTGTGGTLPPNFLLTPQQQNLLFAALNSNKQQLAGSTANNAVSAPNNSFRNSASQQKPAQASAFQESPFLDNYDYDFGDSGFDFSFASEDQPSMIGDLPGTTADSTNNAASVALSDSSETETPEKRSYPDDEDDEDSPGQEHKRRESSGKVPKKPGRKPLTSEPSSKRKAQNRAAQRAFRERKEKHLKDLETKVDELEKASQAANHENSMLRAQVERMTAELNQYKQKVTVMSTTKSLPREKVPFGSAAVSNLGDVNFQFEFPKFGMLPGPPTNKPKGSSPTSPDQQKASYPSPNNNMNNNAQSAQQFKDDLAKFSGVFSPSMSSSATNPSRTSVDSGNYSINGASSSPSASSHSNTGPSSSCGTSPEPFNQSPMGFKPVDTMTTIGEEQSGQNNASQFGNVDLNSTNFDWLSQQNGGQFDPQLFGDYREPQANVLSNPSFDDFFNDALDSDFFTPYNMAPNSPNAQANNQHKKASNLIDQIDAQKESCDDEPPKKQNMNCNQLWEKLQACPKAQTGEFDLDGLCSELTKKAKCSGTGPVVAETDFDTILQKYMGKDVSSSCVAEKLGVELSSTKPNSEAHLGLSA

>FgbZIP_4.6

MNRSTPNFGHEAQEFQDNYTLNGGQEDAIIDPLLKDCSPNATMNAGHSIDNQLFMNSDMWNPPLNNPVDFYPPAMSGMGHTNLTNSPIQPSDTHSSPETPFNGFAFAASRTSSKSSVPSAKSPDADVKPRRSSRTTKTRPQQLSTETATKPSHQRRASKAITVKTGPGEEEGQDEDEDEELDESAKREKFLKRNRIAASKCRQKKKQQNEALEEHLCRLEIEKELLHKQCNGLVDELSAIKNQLMEHASCNDANINQWLDNEAKKFVQRIASQSKAQIPPHQNTGDCCDMHRRSSSVARSIKSDINFDHMPDSMINSNP

>FgbZIP_4.5

MMNAADVELEEFTAFEGGAHTAFSSPAVASVFDFSSSASSSIANLATISPQDLLVQEPFMSAPNSSALTALTSPSIYNESPDFDQYDVSPNFGNAEFDAPGGEWFSLFPSEPSAVPQLSVETSPEMKSDELDSDSQSPPLNRRKSGTSPSTRHSSVAGVNARKRDKPLPPIIIDDPSDIVAMKRARNTLAARKSRERKAMKMDELEDKIAKLEEERDHWKRIALAQSGVQ

>FgbZIP_4.4

MQNSSASGANNPEQHLRAQLELLKNHDATSSSSPTSPAPRDTRHQPLQPAPARPSNGFDHSSNPQDQPRALAAKGEVEAHIHPDLRARANNAPTATMMPIVPPSGHSPGASAGPSNAPIASMPPPHMSPDDHGDGRSKAKR

ELSQSKRAAQNRAAQRAFRQRKEGYIKKLEQQVREYMDMEQSFKSLQSDNHALRDYIVHLQSQLFDATGEY
PPPPHNVDIAQPTQQPPAPAPTPAPAPAPAPAPVPAPIASAPTEPALEAVAQAVAGLAAQEQIERQQYPTAH
E

>FgbZIP_1.6

MGTTTEASAGGESKSPKQSNSSPRPDTTTDARAADPPIKLEAANAEQAKPLAPPPRPGQQPGNTPDYFTVQA
GGSLSLEPNPFEQSFGGAPETPGGTKLPSVAALTSPSSLLPGSNSTPFNWGGGSLRTGPLSPAMLSGPANDYF
GETHHLRGGFPTPNESSLRTGLTPGGSGSMFPAPSPNSQALFAQLASGGATPSTLDFHRTAISAAAKRDQNG
AVPRSQAQPASQPQQPPQQPSVTSQPQDMPNGASNTKSEAKPASGPFDPHDNDAANGLFMLAQGRNGA
QNGNQFAVTSGASGHAHPAPTAPQNMNTSPQMSSINGGSVGSGRGMSEGSMMSDESEQARPNTRGRG
KKNPPATNGRRKADEPPSKTPVHKKSKTNSMDMDMDMMSDDESKMKYEDGSGKSKMTDEEKRKNFLER
NRVAALKCRQRKKQWLANLQTKVEMFSTENDALTAQITQLREEVVNLKTLLLAHKDCPVTQQQGIHGAFMS
QVVEPYNPQMNPYGMAAPMSNQQVMAGQGVQRRFS

>FgbZIP_2.6

MEYQQTLPIDKFQNSPTESLMSIPGDGFTSLFDVTTPSATSTMNPMEMMTPKSYTDDQIPSSLPQIKKEEDM
STPSPSPAPEKKTTKKRKSWGQVLPEPKTNLPPRYGDTLRHVVDESTDTLNRKRAKTEDEKEQRRVERVLRNR
RAAQSSRERKRQEVEALEKRNQELEEAFMAAQEANAKLMSELEQIRRSGAVSYSPSVLDSFRVNPSLSQELFG
SKHTHEPIDGLVSSNTTVDPTALSPVLSPVAESFEEIAEQEPSNEAKPELTESTSPDLTQLSQVGGDAQVVPSAA
NLDAANLGLAPALPGDAAFSLGNSDLLPTSIGADRYILENKYLSSSDSSIIGDDNMVGDAPAFNLNDDFDISLW
LNDDSAISAESMATSDFAAAIQGLEPKIYEPENQVSSENPIQQPHPGASTQGCDVGGIAVGV

>FgbZIP_2.3

MDSPSTSQADDKSAPISEAERKKLRNRLSQRAFRRRQAECIRELRNRVNADQRPDSERVEALQKENKILRQQLI
DVQAKMSKVLASVQLLSDSVTKTLDDTKDGSHSPDEADLERHALDRKRDQHASISSMSSLDLESFDPSILDFEV
PFASADAGQSAPEPANVLTSELINVTGTNPLYSQIPNIWSHQYQMGLEPYITAINATEETNLVLGKDYAFTNSP
FSDHIQLLQKMLKSKLNTLGFVPESHHPMQSVYQPVLMVLSMFNSMTRPDVMAWYAKTRFYHIIELTAWQL
YPSAATYNKLHPRYRPTKAQLENPHPGIIDWIPFPSIRDRLIQLHSANPHIDQIFCDAVTGYVVEALMSDLILGA
PPITVYVRVTDLINTMSRSSGDESVSALLPAPDISTLFSSPAYARAAFNKLNMDKGAGYYKIDPAFFEKYPELWD
QAGDLTASGMPLKPKYQKILTYPKPLDPSTVETYRSFIDFSLDASSTISMGPAV

>FgbZIP_2.2

MDFTGQYNFTNPQPYHQFMPIPPLTPSHSHSAGSDDFNASPPVRKLRRPFHHQNDQFQSFDYTTAQGFNA
NPHQPASGFPGPPTPPGQNVFASQMQGHRQQGGSMRNGSPDEQSIARGGSEEDENLTPAQSRRKAQNR
AAQRAFRERKERHVKDLEAKLAGLEAAQQQSSIENERLKRDLQKISTENEILRATSHTGHGSISPEPATGPLRFK
PTDFYSNVLQNHTNKSPSHRIVTSDDGERLLAAGATWDFIISHDLFKKGLVDIADVSERLKNCARCDGQGPVF
SERSITNAIEQSVASGTDDLL

>FgbZip_1.7

MSEPLISPSSCENDAIADSMLNDEGLHSTPLSGSLPNTHGLEEYFQPESRTQQACLYEDANLEILQDDHESSSA
ATNLIPNSTLHEAEVALATGVEVSASIKPGTNTATKAAGERRNRGKPSDNKNERGKKTAPKKEKEKQYLKILER
NRRAAANCRARKQEQQDKLNAEVEKLEDRHRELSASCNELRETAYQLKLQLLRHGDCDCALIQRYITSEAVNS
VENLILKHSPSSSPNCITIGSTAISASSPTRGLGGNNEWDR

>FgbZIP_1.3

MSAPGQNNVDFDALLDLTEYDGFQSPASLSPAGTSKATFTSPVTAAVAAPVTTAQSLSGPSHNYDMYRQQT
GFVPGAIASTMAVNQTNNTGYQDFRSLDYSTFSPEADLFDFNTSPSQATMGASEMDMDFESHTETQQFFTV
DPSSIEQEVDGLPSPPVLPTQTNNVGRLWPGAHSQAALAKAQAQQRQQQQIIQQQQQAQRQGSQPKSRG
KAPPPSDPLVEQKITQLLNSMRAKPSMPDSQATSPMTNLPRSKKDEEEMDEDERLLASEEGKKLSSKERRQLR
NKVSARAFRSRRKEYITQLETEIANKVSENGDLRTQNRALLDENKRLTDLTRMLLSSPSFSNFLDNLSSNPAAA
QQTPQLKVEPQPEQRQVPKDINPYNAQQSSQQQIGMAMIPEQNMDFSMLTLDGFNFQPQVFVVDTPEVP
EIIDAAVLSGKSSNFVEPIFDSEEEKLEVPAIERPVAAPEVSEPVNAAPIDAEFEXV

>AgbZIP_1.1

MSSSVYERPAKQGAEDTEELAPLSPEAYRLKYKGIYSRLISDIWDDAPETAESLGMADSSSQPWSAGDVRNTD
SPGAKARAATADVTDKAEGQGGGDDSFEKRPLSSVTLNLSDERQLSMQLQDLIANNSDLGTRLLSLLLVSSGN
AVEIISRINSKDRDLSSLDTLRLLKPSSQSPRLSGKEFPSSDADGDRMANRTTCTSIGTNSTTAAKNKDEEELLKL
QLEKRRRNTEASARFRIRKKLREQEKLGKLKQLNGEISSMYKRIDELMEENRYWKRRLDELNERKSKERLDTIRR
RNQAARSDGDR

>AgbZIP_2.1

MRPEEQQHYFYNTGPDIRDPLGVPAALMHTEPGIRWPPQAEEHGMQAQVLSFNDGMCNYPMGAPPETA
GYPQDVTPSTGSSSTPPGSSLGSQNHRQFVSPYSQDGMLDSLDLIEKKKAQNRAAQKAFRERKEARLRELEQK
LKESEQNRDALFREVEQLKRENQVINNENRMLLQRTTNNPSAGAAEGPEKFTFPAKDTAISDPTEGGQKQGI
QYFAGGKKLLTVPATWEYLHKISLQRDIDVYYIMQLLKGAEVCHGFGPAYPRELIDSLVGECLNEDRKS

>AgbZIP_3.1

MTSHTVNDIPADFKSTLPPRKRAKTQEEKEQRRIERILRNRKAAHQSREKKRLHLLYLERKCALLERIVAHVDLG
ALVAARGDAALGRAVTEYEAVAREGSGTPSFAITLTDERSDKNEGPRVCRRATTPKREEPLRVGRDAGLAVHD
GGRGGVRAPGLRAGGEAGAARGGVVLVRRVRAGVRGGAARVRRAAGRERVVEPVTNAVAELPVGRQQYL
QGRPERAVRVD

>AgbZIP_4.1

MRPAGGVHAVHAAAQGHYGVPPATYMSDPVIQESLSPFFQPVGIDVTRLPLTNPPIFQSSLASYNGPPQRRR
ISISNGQIGQLGHMGQLDDEDVMESIYDLQPPPLPQRRNGAKPFSKPVVSHEQFLARQHPPSYQAAYVDVAP
GKPAEGHGAAYAGSVSSAPLHPHPEQPLTRPAHVLDAMASGPDFPRAASSSSLPTLYDAPPGTAAWKRARLL
ERNRIAASKCRQRKKIAQLQLQKDVDILTKENKEIRRELEYYQKLVSKFKRFIELHMETCNGSNGGVQIIEEMLKI
DHSIVGQENDSGDKRDTSEEAPI

>AgbZIP_4.2

MPANDLNSAISMFEMNAGTTGAGAVVAPCSAHVAAYHMGVTPAEQHEALQSSSSTSSSDSSILLGELVFEKF
ACADNLDHEALAKLERSMPMFSESKSSTDLDSAVENFFGSSSDSTPLFEFEGLGKTADPKTWSSLFDDDIPVTL
EDVGAVEPISAAAGTESCFLPTPIIEDAVLKPPKRSLTKRDADSVPASKSASPVSISSNISSPALNGCTSASATSRR
RSSVMKSEKYDHLGVITYNRKQRATPLTPVVPESDDPVALKRARNTEAARRSRARKLERMNQLEERVEQLLQ
KNSELEAEVARLRSLVPSSEQPKYTSRAGN

>AgbZIP_4.3

MSTAGQVKRSFEEQDGPYVGETKRRGNKPGRKPLDTEAKNRRTAQNRAAQRAFRERKERKMRDLEDQVRR
LEEERSSAECEVQSLRGHVIALVRELRRWRARQQGAGGADAEPTAGYDVSWEHGAAGARAERRRLGGRRSR
RRGEEGALRAGPAGARRRAAVASAVAVLVHLVLLRRAANAVFVFRRAHHGRGRPNTAAAPCLAEAVRGRH
QRSLAGGPRLLQHVGQRVARRELRSHAAGRHGHAGSQLPAGHCDTAGKRRLDVGRQPQPDRGHARAFPP
VRGRPARQ

>AgbZIP_5.1

MESNRGSADAKASLEELEKKGLLGAAGVVVPDMAGVGGGVQQGMAAMPVSVRAQYQQAMQEGAGAG
GGAGSGGSQPPLIQLPSMTMPSLMYQLSHHPTHHGSMPQIPVLKQHQQGQQEMGSGSSAGSTANGSAGL
PLLSTTTSTQGIQAIQLNEGEHINSQGQLIGRSGKPLRNTKRAAQNRSAQKAFRQRREKYIKDLEIKAKQYDKLE
QEVLSLRRENEELKMRLADIEKLT

>AgbZIP_6.1

MERNTVHQHVSSFDLEPNPFEQSFASTKKEVGGPGRPLLQQGPPPGHALLGLANGGASDVSQAAAVAGPQ
KSPMRYHLHTHKPPMIQSPPILTPGGSHRLPAMLLSPQVLQPHNAQTESLLQAPGQAHLPALSPLLPGGPHN
GQTTPSFLMGLTKTGLTPNESSIRTGLTPGILNGGQHTSLPLPNGGQFTPGMSSMLSSMPLTNDTRTPGGGG
AASHPHSLSTVLEVPTSTSNSVAEIPMGGSSNPVTGNISLELPPERVKNTLASNRKRSLSNDDAIHGIQNTSDGS
TNSNNKKKTKKSQSAPAEDPELDERDRKRKEFLERNRLAASKFRKRKKEYIKKIETDLQFYETEYNDLTSFIDSLA
GLTGSAKGMGQASSDISLLKLLKQSLMRQDIARAMALVNQIEQHMISTKYIQRNGRNPRLEEDQSQQKQVN
VVNGNAATAGVGSNVSEVRTPLLHDRYATI

>AnbZIP_8.1

MARYSQPVFDFYHQHPSTLDTKTQPSAYPEDDEMSVLDDKILDTTSSDFTSPADHRRHSYEQGPDAFQHRDS
VWSDISQSLSSTQSRQNSQVGHPFFESAPNPFMRMDGLPYVHSQQWSISKDSGSCTPSAMYENYPTDMEN
TSVAPFAGGAVGPVNTVSMPSMTYRQHMAFAPAGAVAMSPQSSQGWMPASTDMPDPSSRPKNSPTYRN
SSPLSVRRDGIRKKNARFEIPAERTLSNIDHLIAQSTNEEEIKELKQQKRLLRNRQAALDSRQRKKLHTEKLEEEK
KHFTQAINELEEELQNMRLREAELLREKEEWMATQQKISEYINSLHMDKDELIRAHTLETADLRKKNNILKETV
EKMERRVRTNVSNEFSDFENLTMESSPWEDFTMVNSLSLDTDSVAPAAQPSQAMVVATNEKGSEKHANEY
PFSWNAFYMCLLFGAFIASNSASLPARSLPRLSEEYRAESANVLKAVLASSPPELAQSSSNQPPVSSSVGLLPTTI
TGAEMAQMTGSAPTSNLDELHETLAMPTKEQEQEQAFALNAEQYNSLTTFDETGAGYKSQQPSNLQQALA
AMRGNAAQARTPLKATSDVYSRSLLWDRVPEKVIRDFRRMVQEYGAPAARE

>AnbZIP_8.2

MAAATISAASVARQITAYLDISPHSFSDSDFLFSSNSSLSSSSTPALFPDLSAAFPPVDLDPNAPQLFYDPLLVPS
VFPGDPASSSGSDQFNDLTTAAGYPSITTTTADDIDWTALGCLPQSEHPANSLGSQSQGLTPSVDSPDPPFNS
TQPRILTTDLSTTHITTTKPIQPQPMPSVTSLSTSRESSPKEKEHLSRITKRQLNTLAARRYRQRKLDKVAQLEEEL
AAVKRERDELKMRVSKLEGETEVLRSMVKDKN

>AnbZIP_8.3

MSAAVASAVSTTLPSNPTAHSSPMDAKKNSVKMDNEASSETKEQKTDGEPQTSLAPPSRPNPSAATDTPDYF
NSVHNPFALEPNPFEQSFGGGSGETPGKSILPPVASITSPALPGTSSAGGAYNWSNSLRSGPLSPAMLAGPAG
GSDYFDSIGRGFPTPNESSLRTGLTPGGGGSMFPAPSPNSQALLNQLQNGGATPSTIEFHRTALNVKKNGIAP
TSNPTGEGDQVPQNITTTMDIKPAQPATVDFGPHDAADAANGLFMLAKGGQSTANQFAAVSNQTAIPPQT
LQTSEILQDQNAARRQSVNVNGVANTREPSGDGSEQSEQAKPARGRGKRNTSTKASSTGNRRKTDDSTQGS

NKRTKLNNGAASTESPSEGESEEEEQQPAQKKKAGDTKKMTDEEKRKNFLERNRVAALKCRQRKKQWLANL
QAKVELFTSENDALTTTVTQLREEIVNLKTLLLAHKDCPVSQAQGLIWNPNPPYT

>AnbZIP_8.4

MSTPNIPHQEFFDFTEGFGEDFTDPTMLSPHLVPTGIMASKDSLGDVPAGTVSPSDLFMDASAPPSTSFTDLS
TPSFDSPGYFSQDTSPVFGADLDLAPGHEEWAPLFPSNDGMSMPFDPTGLEIAAPVPAVKAEPTVSSPTVKP
VSSPARSPTATSRSTTKHSTVAGVSARRSKPLPPIKYDESDPVAAKRARNTEAARKSRARKLERQGDMERRIAE
LSKELEETRQMVEFWKSQAQARARGA

>AnbZIP_8.5

MAEYNGRRAPNFSQYLDDLNAIPSPYDVAMQQQQQQDGFNIDNDLSLFTNTEFFDFDLNLPPFEPVEENKH
NVNQNSDMDFLDILGGEGFGNVNDYAPQMNSINNQSVPVQNAQFHAVPQSQVPGLPNVSAQVNQSVNS
RATASPSQSSVAAPSPTNATSLAAPAPGPKRKHTQKTVPVSVEEAARIAADEDKRRRNTAASARFRVKKKMR
EQALEKTVKDTTEKNAALEARVTALELENQWLKNLITEKNGKSAEEGKKAENDISDMFKKFLAAQKTEGERSN
GNSKIGVGTV

>AnbZIP_8.6

MNQYPYYGGHHPSQQQNSFPLYGLPTPTQNSHGDDFQGPFDPLNYQPPFDPSFNPAAPQFVGGPPPQSPP
ESYTKHSVSSGEHIAGSHYPGSIEGHDEFLAVRSSSEEKDKDGIGITPAQSKRKAQNRAAQRAFRERKERHVRD
LEEKVSNLQQESSNLLADNERLKREIARYSTENEILRATTHSRTHGPSSPKYNSNSGTGSDHRQNGSNEPAQTG
PMVYSPTDFYSNLVPEGQSARLHRVTYCKETGQRLLDAGATWDLIQSHEMFKRGLVDIAAVTRKLKTSAQCD
GQGPAFRESVVRQAIEESVVQDPDGLL

>AnbZIP_8.7

MVSMVEASILNHNDMAMDQVAPKSEPLNEGSISSAVSTPDPEGEVLTQDVAQTQKRKGGRKPIYATSEERK
QRNRQAQAAFRERRTEYIRQLESTIKRNEESLQTLQQNHRTAADECLMLRYKNSLLERILLEKGIDVQAELRLK
AGTPNGPGKPSPITTKAPSLQQAAISRSSAQRHPSGLAPKEPFSVPQSRDGGFGIPSPQFQATPPSHVSSPSHA
KSPNYGFQGALSPAGVDPQAQRSQMLTHSRNISQTSPPMSVGQPEPTEPKSAVSASMGSRAPRLPSAYYPS
PFQKHYDQLEQEYDAQADMIDDEHESSVGTSSFVPGYNPSSSVSNASHPMNPHGMNPYNHSSGEAVNGAY
GNTSAMMGNYEPMLDADPFGLSASMHFQTPFSYEQNNARQ

>AnbZIP_8.8

MSDEQIARQTAASSFDKLENFNFLLSRHDPEVAKHRQYSFDTDAAASLAHVPNLGMPYDPTEGMGGLSVSS
YESIEDEHSPIDVRGYPYHAGDKTINYSVSDHMLSHSAYPLYPPISYGPDEVGHAPGAMTPSDVSSSISPPNGQ
IGNNKYSTQISGDHIASALNQEEHSRRAAEEDRRRRNTAASARFRMKKKQREQTLERTVRETTEKNASLEARV
AQLEMENRWLKNLLTEKHEAASSRMPPPPATDVSLNHPSTTVTGSGQKHIQPKKKGVGTDN

>AnbZIP_8.9

MSRTDSFLQQGVSYGNAGIISASELQRFQTSMYPSDIPNLAGNPFSLSEDNMWAPHPTTHPTINPSCIAAGG
ASDVHSYLTNHPFDELNHKQGPSNRRRSSAETELDQGSYRVRHGQVTPPSDHSPSTVYSVPSHDSKSAYLPST
IEGSSESPPKRRRSGSSNMSGSRGSTRASTSVEPTSPGDDKQEKTRARNRLAASKCRQKKKEQNHMLETRYE
QEKMKNEELTRTVNSLRDAIVVAKDQLLAHSECGHESIKAYIQNMAKNISIQHEQFDFGTAPAQYGCHSERKP
SGFGFDVHPPTA

>AnbZIP_8.10

MADYNSLYQHGLYLSPDQQDLLLAALSSNNPPSKQKQNVQKPELGTNPTNTPGQASTGSFNTSPAFDGSHQ
FDNLNYDESPFLDFNPELEWDFPGSENLIGELPGSATSDDHEVGEKRKDSNSNGEVNGKKRRESDDKSDDKT
SKKPGRKPLTSEPTSKRKAQNRAAQRAFRERKEKHLKDLEAKVEELQKASDSANQENGLLKAQVERLQVELRE
YRKRLSWVTQGNALSAINSYPGNANRMSGLNNNDFMFDFPKFGDLPGGRIFNGSVAKTNQNKKDDTPIPGI
LRHSALQAANGRASSSASPKTVTSNNPATKSPVTADGRLTSHTSSVYNYHQPGQGHDTSTSDSPSSSSDSHQF
LSSSGTSPEPSVQSPDNQAKESHEGHTCTIDGEKSFCAQLGMACGNINNPIPAVRQRSESATNTPNAPSSTDN
VPGIDFMAQQNGGQFDPLLFGDWREPQDAVLSQDFNTFFDDAFPLPDLGSPSHNLTEVGLGAQQKKSILEE
MDNKEEEEVVPGEDKAQMLSCTKIWDRLQSMEKFRNGEIDVDNLCSELRTKARCSEGGVVVNQRDVDDIIG
RV

>AnbZIP_8.11

MAAQPALAIAPSAAPLAPALVAKPTVSPSPGPGTPGSVTSKEWIIPPRPKPGRKPATDTPPTKRKAQNRAAQR
AFRERRAARVSELEDQIKCIEDDHEIHVATFKEQIANLSREVEQCRTEMGWWRDRCHALEKEVSVERAARETL
VKELRSSLPEKNTSGTDAVPLPPRSSRSSRMELEKSSPVDRRSELGEEVPLGCNRCSTSHCQCIEDAFGMPPIE
MNRAPEPKIKPEPEEMEIDFTTRFAAPHHEEDTAASPVASPPVDPCGFCQDGTPCICAEMAAQEEERRRNST
FESNRLAPIQNISQFTPPPSDSDVRSDNVTLPPISQATAANPCANGPGTCAACLSDPRRTLFCKTLAASRSASGT
PSGCCGGKGRDGGCCQSQSRTSAPRRSNTDRSATPLTLSCADAFTTLSRHPNFSRASDELASWLPKLHTLPNP
RDVSQTTPASRAAMEVEAASVMGVLRYFDRRFADK

>AnbZIP_8.12

MTSQTTFYQPDPSLDSAIAPESLFLGQEFSPEFLPLSLLEKDGHHHELPLEHMQFSPHQPAMVSVRAMQGES
TNANEDMMRPRYRTRALRPAPRDTRKDSTDDFNQPGFLNSYGPMPSFSSFSSTSTTFNPAEQDPEFRFSPHS
DISSNKSSSPLRWQTGDAEKRAKHLERNRAAASKSRQKKKRETDQLRTRFQEVSRRKSTLEIEIKELHSQLLSLK
DQILMHSRCDDEAIHLYLGRMVKQATKHDSISSASSGTSASSREQDDEGDRSFGQRHGSECISPRQTVTSLHT
QGPPHHHNLNHSRTSLNPAGMDLSNAHQGPMRMGDGSGLPCGVEKPIMNQMFSQHDPNNFDLQISIS

>AnbZIP_1.1

MSRTDSFLQQGVSYGNAGIISASELQRFQTSMYPSDIPNLAGNPFSLSEDNMWAPHPTTHPTINPSCIAAGG
ASDVHSYLTNHPFDELNHKQGPSNRRRSSAETELDQGSYRVRHGQVTPPSDHSPSTVYSVPSHDSKSAYLPST
IEGSSESPPKRRRSGSSNMSGSRGSTRASTSVEPTSPGDDKQEKTRARNRLAASKCRQKKKEQNHMLETRYE
QEKMKNEELTRTVNSLRDAIVVAKDQLLAHSECGHESIKAYIQNMAKNISIQHEQFDFGTAPAQYGCHSERKP
SGFGFDVHPPTA

>AnbZIP_7.1

MARYSQPVFDFYHQHPSTLDTKTQPSAYPEDDEMSVLDDKILDTTSSDFTSPADHRRHSYEQGPDAFQHRDS
VWSDISQSLSSTQSRQNSQVGHPFFESAPNPFMRMDGLPYVHSQQWSISKDSGSCTPSAMYENYPTDMEN
TSVAPFAGGAVGPVNTVSMPSMTYRQHMAFAPAGAVAMSPQSSQGWMPASTDMPDPSSRPKNSPTYRN
SSPLSVRRDGIRKKNARFEIPAERTLSNIDHLIAQSTNEEEIKELKQQKRLLRNRQAALDSRQRKKLHTEKLEEEK
KHFTQAINELEEELQNMRLREAELLREKEEWMATQQKISEYINSLHMDKDELIRAHTLETADLRKKNNILKETV
EKMERRVRTNVSNEFSDFENLTMESSPWEDFTMVNSLSLDTDSVAPAAQPSQAMVVATNEKGSEKHANEY
PFSWNAFYMCLLFGAFIASNSASLPARSLPRLSEEYRAESANVLKAVLASSPPELAQSSSNQPPVSSSVGLLPTTI

TGAEMAQMTGSAPTSNLDELHETLAMPTKEQEQEQAFALNAEQYNSLTTFDETGAGYKSQQPSNLQQALA
AMRGNAAQARTPLKATSDVYSRSLLWDRVPEKVIRDFRRMVQEYGAPAARE

>AnbZIP_8.13

MATLAENPVVAQTSYDQDLDSFLNLDQLGYTPSEPARSKIGLPTSHPALPSSEYVSSDARSSSFASSSQSPVAFP
APSHQYEEHKQQTGIPPGALAQAIPFNQVMPFGATNPGFAVNGDMFQPQIKRDEAPLDFNSTPTRNISEMD
LESDSIMATVPGFYVSQNSTNNQFVDPNALGGQEVVPMGSSTQVGRMYPGMHQQAAMAKAAQQQRQN
ELLRQQQQQQQQQQQQQQQQQQQQQQQQQQIHHPHAPNASVDERISRLLQQMKSASVTPSDSS
PSPSAAPLTRTKKDEQDMDEDERLLASEEGKKLSSKERRQLRNKVSARAFRSRRKEYIGQLENEVAQKTNEAH
ELRQQNRALCDENARLTDLVRQLLSSPSFSHYLDEQPNVNGLPSAQVPLPHLPQQSQNSTIPQSALQQSNPV
KEQTPNHGQQGYQMQQQNSQMGMMMVPSQGIDVSAMNMNNGGWNSGIDFNPSVFAVLEVPEPPVL
DFDTLSGKPSGLEGISFTDVSSKRDASFLSFSPHEEQTAVDAGEVNVDVDESDPVLALFADQPRRSVSTIAQDP
SLDGIEVGKASNYELVVESDFEAAASRLAYLCKTPAASIVRSLKPSEKDNSKHYTKKQKRSFVYNTSPILEPPQIPK
IGITQMKFYYQILTTPTADTPGSALLLHFPGKRYLFGQIAEGFQRACTERGTKLTDVSDVFLSGRMGWDTTGG
LIGMILTKADARASSKEALEALEREKEANRQKRGWTKKDTKQTPANAELEDPQHDLTVHGSKNLAHTLATAR
RFVFRQGLPVHTKEYDAESVAKRLRTDQSDPFEVPTFSDENIKVWTMPISPSSTTPRSQSPKKRSLDEFREDVR
GLVEVDQQSKDQLVRQSVVSHMFDSTWTLDALEETRLADVKMPAQIFVRNPETKDLDKYTGPLPGGDEEVP
EMTVLVRKPWPGASISKIPTTTPCVESLCYIIKNHDLRGKFDAKKAIALNVKPGPDFGALTRGETVKATDGTMV
TPEMVLEPTKPGKGLAVMDLPTSDYVESLLNRPEWKSPSVASNLAAFFWILGPGVGEHPRLREFVASMPNCK
HIVSSTDYCPNYLTMQSVAGSAIRMARIRPDNYLVPIHDNNSVPQTQSSLEDRMAVDSQNHSFERAEPGLIVS
MEPEFKINSDEVPRRLNAGSILTKMPQSAVRRARLVTKRLQHPLRQEKIQQFVSDIPGADAEIITLGTGSSAPSK
YRNVSSTLVHVPGRGYYLFDCGEGTLGQLKRMFSPEQLREVLQNLRLIWISHLHADHHLGTVSVIKAWYQEN
YPSGVAQLSEPEKDIGKILEEKRLFVVSDLMMIEWLEEYAGVEDFGFAKLTPLSAYLYRTGDEGMKWSFKYRH
CRADGSYRGREVEHIKPEQSELKFNCKGSPGEELSAKLRKATGLSDILTAYVSHCRGAMAVSLIFPDGFKVSFSG
DCRPSPTFVTIGQDSTVLIHEATFSDDMVGSALAKKHSTAQEAIEVGRKMRARTILLTHFSQRYQKIAHFNQPK
ELVQDKSVTARDFRAHKSIRRKQETAAAEAASTDIPFKNDIDAQENIALDEPAALNPDATESLELSEPSELPEPD
APPRPIVPIIAAFDHMRVRVRDMYTLEQYAPAVERLFDIIERASKLEQNQAREKRRREVEENEKRKEFRRENKF
KTKQEQMSQEQREMAKAEKEARVSDRSSSQSRPQSPDAKVSIWDAPESESGWSSDESGKDA

>AnbZIP_5.1

MSDEQIARQTAASSFDKLENFNFLLSRHDPEVAKHRQYSFDTDAAASLAHVPNLGMPYDPTEGMGGLSVSS
YESIEDEHSPIDVRGYPYHAGDKTINYSVSDHMLSHSAYPLYPPISYGPDEVGHAPGAMTPSDVSSSISPPNGQ
IGNNKYSTQISGDHIASALNQEEHSRRAAEEDRRRRNTAASARFRMKKKQREQTLERTVRETTEKNASLEARV
AQLEMENRWLKNLLTEKHEAASSRMPPPPATDVSLNHPSTTVTGSGQKHIQPKKKGVGTDN

>AnbZIP_3.1

MAEYNGRRAPNFSQYLDDLNAIPSPYDVAMQQQQQQDGFNIDNDLSLFTNTEFFDFDLNLPPFEPVEENKH
NVNQNSDMDFLDILGGEGFGNVNDYAPQMNSINNQSVPVQNAQFHAVPQSQVPGLPNVSAQVNQSVNS
RATASPSQSSVAAPSPTNATSLAAPAPGPKRKHTQKTVPVSVEEAARIAADEDKRRRNTAASARFRVKKKMR
EQALEKTVKDTTEKNAALEARVTALELENQWLKNLITEKNGKSAEEGKKAENDISDMFKKFLAAQKTEGERSN
GNSKIGVGTV

>AnbZIP_3.2

MTSQTTFYQPDPSLDSAIAPESLFLGQEFSPEFLPLSLLEKDGHHHELPLEHMQFSPHQPAMVSVRAMQGES
TNANEDMMRPRYRTRALRPAPRDTRKDSTDDFNQPGFLNSYGPMPSFSSFSSTSTTFNPAEQDPEFRFSPHS
DISSNKSSSPLRWQTGDAEKRAKHLERNRAAASKSRQKKKRETDQLRTRFQEVSRRKSTLEIEIKELHSQLLSLK
DQILMHSRCDDEAIHLYLGRMVKQATKHDSISSASSGTSASSREQDDEGDRSFGQRHGSECISPRQTVTSLHT
QGPPHHHNLNHSRTSLNPAGMDLSNAHQGPMRMGDGSGLPCGVEKPIMNQMFSQHDPNNFDLQISIS

>AnbZIP_6.1

MQPALAPAPHPSMQTSAQIYNLPSSAYLALVTSASLVKKAAVAVCKVATREITDDLTRASYGVVPTASSWRP
WSNKDHADQVLHDSLLAAQHLSQHPQQPRPQQPNAQPHHLQPTATTSPRDQNNIDPAISGGAMLPPSQP
PAQPEPTVEDETPKTYGKRPLSTSKRAAQNRAAQRAFRQRKESYIRKLEEQVKEYEVMSQEYKALQAENYQLR
EYVINLQSRLLDSQGEVPELPGNIDLNQPRTEISVPQPAPRPGQAGASAPPQGSPQSQVSIANDDMNSLNRIA
EAGLGMRKHPNEEAFLSNNFQARRGRGDETADPSETKTEPPTHGLPMSRWFISGYDSLAERSDNPMASIPEE
RSAEHRARSYQLEMFEASLKGNIIVVMGTGSGKTQMYAACPGLRSLTDVSQRSPAYHTRAGELGWEDKVEL
WTEQAVWDAVLEGLQVIVSTPAVLHDAMTHGFEYGPGAVPRILGLTASAGSSREGLQTIEMNLNSVCTTPQ
AHRQELLEYTHMPELRRVLYTPLMKENASLWEGSTLQKLLERDNTYCSGQMKTFVCKAVHIFQELGIWAAEY
FIRASVEELLSHAYVHSKIDLDYDEREYLVNILSKSPVPDIDVHSTDPKDFPVSPKFEALISFLMSTEDINFSGLIFV
EQRAAVTVMSYLLSTHPSTRDRFRTGSFIGMSNSTNRKTMLGDLLSAKMQPDTLDDFRYGRKNLIVATDVLK
EGIDVSACSVVICYNIPKGFESFIQRRGRARRQNSTYSMMLSTEDDGSTLDKWQKFEKIMEEACLEDRRRTEEL
RALGSLDEDVCTRFCVRSTGAILTAEYAMQHLVHFCDTLPRQNYVEDKPEFSFERNDGGLLRAKVILPSSVNPK
VRRAEGKAWWKTERAAKKEAAFYAYKALYEHGLVNDNLLPLTKSREFTRKDISLLPAVQKVSEQYDPWVDW
AHLWSSTNLYQNRILVRQNEEDTSMKFITPTATPPIAPMKLCWDSETTYTLEFEAAGAVSLTAENIERMRAAT
SLYLQATTSTPLAGNKDYIALFGPDLPWDELETWLKKNQGHEPAIQVFSSQRPLDRMGVVRDRSRYGELLIFK
RWLNRSGDLELECDPYPSKRRNLLQRQTLAKKRPAEDEILGSPTKKRILSASHCTIDRLPASETVFGRFIPVILDRL
EAALVATRLCETVLRDIQFQDLRHVITAITMPLAQAPTDYQRYEFFGDSVLKFTVAASLFYNNPNWHEGYLTET
LHALVQNARLTRAALDQGLDAYIISNRFTPRKWSAPLISEKLYASASTRSMSAKVLADVVEALIGAAYIDGGLHK
AQSCIVRFLPEIELPETKLPRPESMPMSKDHKKPHLIQQENLENHIGYTFKDKTLLMEALTHPSCPYDTSIQSYQ
RLEFLGDAVLDMLIVDLIRAHHVECQQGEMTKIKHAIVNGHLLAFLCMQFKWAMPSPLTPSIDTGTETETEIIS
PPPKTLSLYSYLRYSPSRPLPLHVEPESGSSNALTRHNLLCPSILHALNNTTAYPWSLFSAIHADKFFSDVVESIIG
AIFVDSGGDLGACAGFIERLGLVRIAKRILDERVDVTHPTQRAQIELQKLAARLGCNDGFRFECRTVRDLSSGKR
KTLEVDINDHYGDEDPAVLGAEGPELTYTCTISLATLRTNQDFGRDLDDIVVTGCLSKEDAEIQAANLVIELVGR
LESGRLYKKNMDLDIDTGVQVDLDLDMNLDPGITTG

>AnbZIP_2.1

MAAQPALAIAPSAAPLAPALVAKPTVSPSPGPGTPGSVTSKEWIIPPRPKPGRKPATDTPPTKRKAQNRAAQR
AFRERRAARVSELEDQIKCIEDDHEIHVATFKEQIANLSREVEQCRTEMGWWRDRCHALEKEVSVERAARETL
VKELRSSLPEKNTSGTDAVPLPPRSSRSSRMELEKSSPVDRRSELGEEVPLGCNRCSTSHCQCIEDAFGMPPIE
MNRAPEPKIKPEPEEMEIDFTTRFAAPHHEEDTAASPVASPPVDPCGFCQDGTPCICAEMAAQEEERRRNST
FESNRLAPIQNISQFTPPPSDSDVRSDNVTLPPISQATAANPCANGPGTCAACLSDPRRTLFCKTLAASRSASGT
PSGCCGGKGRDGGCCQSQSRTSAPRRSNTDRSATPLTLSCADAFTTLSRHPNFSRASDELASWLPKLHTLPNP
RDVSQTTPASRAAMEVEAASVMGVLRYFDRRFADK

>AnbZIP_4.1

MADYNSLYQHGLYLSPDQQDLLLAALSSNNPPSKQKQNVQKPELGTNPTNTPGQASTGSFNTSPAFDGSHQ
FDNLNYDESPFLDFNPELEWDFPGSENLIGELPGSATSDDHEVGEKRKDSNSNGEVNGKKRRESDDKSDDKT

SKKPGRKPLTSEPTSKRKAQNRAAQRAFRERKEKHLKDLEAKVEELQKASDSANQENGLLKAQVERLQVELRE
YRKRLSWVTQGNALSAINSYPGNANRMSGLNNNDFMFDFPKFGDLPGGRIFNGSVAKTNQNKKDDTPIPGI
LRHSALQAANGRASSSASPKTVTSNNPATKSPVTADGRLTSHTSSVYNYHQPGQGHDTSTSDSPSSSSDSHQF
LSSSGTSPEPSVQSPDNQAKESHEGHTCTIDGEKSFCAQLGMACGNINNPIPAVRQRSESATNTPNAPSSTDN
VPGIDFMAQQNGGQFDPLLFGDWREPQDAVLSQDFNTFFDDAFPLPDLGSPSHNLTEVGLGAQQKKSILEE
MDNKEEEEVVPGEDKAQMLSCTKIWDRLQSMEKFRNGEIDVDNLCSELRTKARCSEGGVVVNQRDVDDIIG
RV

>AnbZIP_7.1

MAAATISAASVARQITAYLDISPHSFSDSDFLFSSNSSLSSSSTPALFPDLSAAFPPVDLDPNAPQLFYDPLLVPS
VFPGDPASSSGSDQFNDLTTAAGYPSITTTTADDIDWTALGCLPQSEHPANSLGSQSQGLTPSVDSPDPPFNS
TQPRILTTDLSTTHITTTKPIQPQPMPSVTSLSTSRESSPKEKEHLSRITKRQLNTLAARRYRQRKLDKVAQLEEEL
AAVKRERDELKMRVSKLEGETEVLRSMVKDKN

>AnbZIP_2.1

MSTPNIPHQGEFGCPGSFWLGLRTNLDDPEFFDFTEGFGEDFTDPTMLSPHLVPTGIMASKDSLGDVPAGTV
SPSDLFMDASAPPSTSFTDLSTPSFDSPGYFSQDTSPVFGADLDLAPGHEEWAPLFPSNDGMSMPFDPTGLEI
AAPVPAVKAEPTVSSPTVKPVSSPARSPTATSRSTTKHSTVAGVSARRSKPLPPIKYDESDPVAAKRARNTEAA
RKSRARKLERQGDMERRIAELSKELEETRQMVEFWKSQAQARARGA

>AnbZIP_8.14

MKSADRFSPVKMEDAFANSLPTTPSLEVPVLTVSPADTSLQTKNVVAQTKPEEKKPAKKRKSWGQELPVPKT
NLPPRKRAKTEDEKEQRRIERVLRNRAAAQTSRERKRLEMEKLESEKIDMEQQNQFLLQRLAQMEAENNRLS
QQVAQLSAEVRGSRHSTPTSSSPASVSPTLTPTLFKQEGDEVPLDRIPFPTPSVTDYSPTLKPSSLADDVVRPAV
SVGGLEGDESALTLFDLGASIKHEPTHDLTAPLSDDDFRRLFNGDSSLESDSSLLEDGFAFDVLDSGDLSAFPFD
SMVDFDTEPVTLEDLEQTNGLSDSASCKAASLQPSHGASTSRCDGQGIAAGSA

>NcbZIP_1.1

MGSATFQQPSYYRTPPLNVDTSCHGQKFFDDDEILDDDILNQSTLNSALEMSPPLTDSRRDSFAVSATALFSP
KSDDSWPPVEMQSVPSNNPFFGQHNNNPFMQMDQNQQSTYQNHWGMMSSGAATPLQSFDGLPQAEY
ESSTVPLFRPATVQAQTPFSNPNNQAVMFQQLGHHGIPAVPASPPKEHWINQELKNQAMAKRNRPTTPLIR
SHNELRRGDGIRKKNARFDIPAERNLSNIDQLIAQSTDEQEIKELKQQKRLLRNRQAALDSRQRKKQHTERLED
EKKHFTEVITNMEEQMNALQRDMDQLLREKQSYEAYISELKNEKEEIIREHTIETGELRKKVNVLTNHVQALES
AAMSTPPAGPNVSGFPGAYSEMEGMAMDGSWDNMPMFGEFTMEQQPSEVKQEMQIAPTKKSEISLSAE
NEKSPSQGGLLFMLFLVGAFVLSNRSPPSIPRVSEDIRVASATLLDNVLKDAGLPQAASGLEAMAPQPSGSSW
AQSTGSSLPVAASGMNGVAPSMLGEMADALTQPTEQQTNEQLFGLTAAQYNGISSQDFLQNAPAERSTSK
GRRNLAEALASMRAAANKDSAAEVYTRSLLWDKIPRDVVRDFAKMVAESQQVAAANDADA

>NcbZIP_5.1

MGSSTGAAGRDGHPESKHEPPLKPSESTTNAEPAKPLAPPPRPAAQQQSTNSPSDYFSQNPISGSLSLEPNPF
EQSFGGAPETPGGTKLPPVAALASPSSILPPGSTPFPWGGSNSLRSGPLSPAMLSGPTTSDYFGDHIRGGFPTP
NESSLRTGLTPGGSGSMFPAPSPNSTLFAQLAGPAATPGTIDFHRTAISAAAAKAQAQAQAQQQAAHQHQ
QQSQPPSITSQPASDLPNGIPPLKPETKPPTGPFDPHDNDAANGLFMLAQGRNASQPPSQSFNVVSAPPPPP
SSHPHTVPAPPVQPVNTSPQMNGNVSIAGSSARGVSEVSIGSDDSELARPNTRGKGKRNSTSAATTGSRRKA

EETPAKTPANKKTKTNGSVSSLNGMDYSGSEDESKPGKDDGTGSKSKMTEEEKRKNFLERNRVAALKCRQRK
KQWLANLQQKVEMFSSENDALTATITQLREEVVNLKTLLLAHKDCPVTQQQGLHGAFMQQAIEPFSHQMN
PYGMGAGIPNQPVGMPPNVAPRRFS

>NcbZIP_5.2

MDTPSHFFSDSLAYEDSNPYQEDDHDVPYEVPHEIPHDIPHDISQDVPPDVGMSEAGLQSYHVFDHRLSIDQ
SPMSGDGPTLGERPGLWDGFAEPSAMETNNSLFVDPGLYGGRDQHVNGLEGVRDSIDANTSCLSDVMSQI
STRRSSSNKTHSHRSSKSGSTSTDITPPDQDPPKKRKQRSKKDPNMEEDDHKRNKFLERNRLAASKCREKKKLY
TQELEGTKINLEARNVSLQREYSILLSEVSDLKHQLMVHAKCNDRNIDLWLENEARRFVQTSDAFGQTFASLG
QSGQAASVPGVPGSPKSRHASIAPNYPAMPGVQLGVLRPGAEGQGGGGGGGGGGLTYGQSPGILAPPTNT
TPPTHQQPGSGSGLYTPPGGANGGYSNINPPGMKKEPDINYDHMPDDMFSDQSTFGSG

>NcbZIP_1.2

MDSWAAHSPSPANLKFENPAESLLATPGEIFPEVFGSDRATSATPSLDVMSPVSLADDVDLTALATLTVPQIPP
RSTPASTPAPETEKKPVKKRKSWGQVLPEPKTNLPPRKRAKTEDEKEQRRVERVLRNRRAAQSSRERKRLEVE
GLERRNKELETLLMQAQQINQTLLQALRENGVAPTIATRPASFDGLNPTPVTFSQELFSSQDGHNLSKHDSSL
EQLFPTIKTEETVNPASLSPVLNPLPEMEEDGEKEQSTAAQPVADATSTATVDTSPDATQHPAVVLCDDLQCR
SAEVPRSKCLAVSQQSQPPSLDPLSLLLLSASAISWMISFSQRPLMLIATSMKRNFSLPPAPAILMSIPRTGLLAF
WPKGAMLPSKEVLLTLI

>NcbZIP_1.3

MDYTNPTYFGAAAQPPYHFIGIPPLTPSHSNSASSDDFNASPQEIFDQFPNGLPHDQFQNFEAFAQFNNQST
TFAGPPTPPTQQLLPTTQPTNGAIHLQHQQQSAADLLRSLNNAKGDPADEARARRQGSNSDEDENLTPAQS
RRKAQNRAAQRAFRERKERHVKELENRLQQLEEEAQVTRSENEKLKQDLQKISTENEILRATSLAAVGAAAAG
SPLGSAGTPMTTGPMSYKPTDFYSNLLENHNEKTPSHRVVKSESGERLLAAGAAWDLMQNHELFQRGLVNI
QAVSELLKGQAKCDGQGPVFEERAILEAIEQSVASGSDELL

>NcbZIP_2.1

MAGSRINFTNYLRNLNVQEPQVEEYVAPNDEELALFTNTNFFDYETGQNTDYQAPPVKPDAVVAPTPVETAA
TSPEVPTDAFMTEFLSGLDQGLEFAAPAADFNFGDFTTTYTSPTIPAYPDTLGQLQPIQPNPQAAYPPVSQHH
ASHHVQHPHQPGYVLSNPPQLSGNKRKASDAMSVPPTPGARVMSFEEASRLAAEEDKRKRNTAASARFRIK
KKQREQALEKSAKEMSEKVTQLEGRIQALETENKWLKGLVTEKHGSKEDILKLLREFSAHAAKVSKDAAAAAA
DKAEAAADKADAERAREESSFCVSTSSPSSDESVDTDNKKRRKD

>NcbZIP_6.1

MTSTQNPLHNLVLTPQQQSLLFAALNSNKPINTQADLSGLSAMYNGTSAQGLDPMGFQHTPNFGYDYGLD
GQHDPNFDFNFDTGDQSHMTDDRSNAPSDSKSGSADVESPDKRSHPDDDEEENSEPKRREGEGKVAKKPG
RKPLTTEPSSKRKAQNRAAQRAFRERKEKHLKDLETKVEELKKISETANHENEILRKKMEKMSNELNEYKKRLSL
MTARPPMVSPRAMPFGNSFVSNLNDVNFQFEFPKFGGPLPGPQSANQTKKGTPVSPPLSSTGRLEQPTNGV
STGTTPNYGQIGLDTQTKEDLAHYSADLFTPQSARGEGANFSTRGTDSQYNAGAGTSTSSPSSCASNMGGAS
SSCGTSPEPLTHSPVGLKSVDTMATIGEEQPGVIGSGQPDFNTFGVDNTLNWLPQDNFQFDPQLFGDYREP
QNNVLNNGFDDWLTNDAFDADFLAPYNLPPTAPGLLPPQPEAESQPPKKDLIAQIDEVNASDDGVREQLDC
SKIWDKLRSCSSVQNQEIDMDALCSDLQKKAKCSGYGAVVDEKEFKSVLKKHLSPEAMARCEKDCEEEKARK
AAATL

>NcbZIP_6.2

MFSELDLLDFATFDGGATTEAAFASPANQTYDLSSSVPSSVSNMGTVSPQELLLHEPYLSAPSSTALTALTSPSL
FDGSPDFDTFDISPNFGHSDLENPDTWFSLFPDATPLPQAQAQVQTQPQTQTQTEQQTQPLPELVQSVQPT
VQPTVEQTVHSVEASPATPSEDLEVLSPGSGHQRRKSSVSPPSGRHSSVAGVGSRRRDKPLPPIIVEDPSDVVA
MKRARNTLAARKSRERKAQRLEELEAKIEELIAERDRWKNLALAHGASTE

>NcbZIP_5.3

MEDFNHLVDMPTSESSFRYMYAESDRYLDVHSSGSQSSVSSPYDVASFMDPNIYAGTYQGLESEDESSQQPT
QEQKPKRKRENRYKNAPPSVLSRRRAQNRASQRAYRERKDQRIKDLEQMLNEAKQHNNALGQAYAALQAE
YEALKASQFKDIGYSTANNLTYQPSPLPTTSTALSSTGFDLDLYAYHELSANGGYHMFGKTA

>NcbZIP_4.1

MPVGVPPQPEQQGAMPAPSTPVGVSQMGIVSPGDSAGDGRKSKRELSQSKRAAQNRAAQRAFRQRKEGY
IKKLEQQVRDYSEMEMSFKALQNENFALRDYILHLQNRLIDVQGDYPQPPPNISLATPHTQQLQQQQPPPPP
SVPHGIAPDPVQAVQQVPPATSAPSASLEEAAQAVAGLSRSDHHMGGTSRDPYNTAVSAAAAAAAAAAAA
AASRTDDDARTAEVIQRQLQADNNSGMSDGLPLQQAAI

>NcbZIP_1.3

MHLSLDHHHLSSDDPYAAASLSHVTDTLSTTMGLTMPSGRNTTTLGMDMFRTASNNSGSSNNNMGYSQL
NATTSSSSRDHSTTPPTSQSSGSTSPTASTSHHGHGGQGHLYPGLTLPSPVDASSKPKRGRPPGPKKRALSPSV
AAEAELTDSEDIMIKRQRNNIAAKKYRQKKIDRIQELEEEVDQIKKEREELRLMLAKRDAEVGMLREMLAMAK
QGR

>NcbZIP_2.2

MALERSKPVRRADGFHLNRQQPPTPITGHSDMNVTNSVNKTVNPSELFLDANQAGTAWDQSPYHSDLLFA
GLGFDNSFAIGQHANDAFSALENTKRLTANFPTQPSLVTQSQVLEAQPTGNGPTYLDSIQHSGPNFDWAGD
NSINSINFSIPTPFTIPQQPVSGWNTTPATTPLAPRQHRIPGPGSGPGRNFGLANEQPNHLSEKDKYLAITHRQ
TDQYHRIEKRTKLQRLVNNMKTLPVVIEDRPCEFPKLSKRGKHRDEDDLDEDERVLKGEEAKGMTSAQRRQL
RNKVSARNFRARKKDYINDLEEKCKEQHRTILQLEANMRAQITDNERCKALIVELLGLPEMANVLGHYEKKISA
MAQTIAPAQSQINMDSSIADSIGMPSSQSQSQSMFSTSTWAQQLGDESAEVDDFSDEIGIDPAGLDFPR

>NcbZIP_5.4

MTAVASAAHMAGQMTSVSMSIEQQQVQHRQRQNLQSDMDLSALQQTSTSSSSSSSSSSSTTAATFAKRD
SPSAVSTPATSAPSPTSSCGVSTPASSTSITANTTTTNLTGVPPIAPRPSVAPKPAPIRAASVSSQGAMSPVDCP
TPGGGGPLSMTTKEWVIPPRPKPGRKPATDTPPTKRKAQNRAAQRAFRERRAARVGELEDQLEEQKEEHER
VVSELQGRISHFEVQVQTLQSRCRWLEDMLEKEKQARNTLKNSWDNNNNMSPQSFLSQQSNGYTLQQQQ
QQPTQQTQLAPKVDSVPIAEPRPTAQPFSISQIISPPEEAPQSPLDVTCGNCQSSGSCACAEELMQSSNVLMG
CGGCTPEGRCACLEESIRVLAAADLKRPLPPSSPSLGPDEKRQRSDAGVEDMQLETDFTALFSSKKPETTMTPL
PAPAQQQSQPITSVEMHESCGFCKDGTYCVCAESMALSAASMTVASVVQQVQMHTPPPSENDVVPMPLE
VTATGAVKLPSFGSLNRASNTNQDMQSKRPVKTGGCGPGGPGTCAQCLADPKSGLFCRSLAANFERNRQQT
DNSSSAAPPSGCCGGGGPGGGCCKTEKPDDNDDVVPINLNRYKTANQLPAPTPSNPISSSSSSSNNVAASNF
ALSLSCAEAYKTIASHRHFEEAADDIGSWLPKLRAVPANPPPNRGSSGYYGDLSVFLAFVGPAHAGLTSITQHS
CPATQLVSTTSGARKRRGGSGLHFLMRPNLRPNPIITPATATPPILCINVSRLGHILRPFPQLPQFPSIFIPSCLLV

WYLQPLPNVLIIRSLPADRLVAELVGLVEKAGICWPIICIVVLSLASLTVVLGDGDAFMLHVVIDEPACRLLSVS
MRPMLAYRDFHREKLFMGYAPSLGDRDSDADTDTDPFGSPEDGREPEPDPGPDRCQLSFIIL

>NcbZIP_5.5

MDIQHSQPADSTVPTELTMQSQYTANTAKCAELLKGCEKRNELLPQLIDKLVTAKDIKAYQRVFEYVHGEHG
HYQKDLKEAAKLLDQNRLAENTEIERRAANQAATHKAQYETKLQVEKELYQKELDKIKQNMYDTVIKRKAKLE
ESYKERNEDFAKRKETSEASLKDKEDKLAKRTEELEASFKNKQAELEASYAKKEQNLDELYKERNARVQSEEKTR
DCILDQRERTAIGLEADAETRMQELQTLKQELDSQKKELKTKEQCFHRERDEFQKKVEEQTKTDGELKRQARA
TTDQLDAREKALKQAQEAFDQLQAEKEKALKQERSDFDKLKTEQAKALRQAQSDFDKLKTGQEKVLEATKTT
FLAESLETIDHAKDNFNIGLGNGLAALANRVPDLRSIEAWITNGAQTFASHQTRIISARLDQAMRQSNGSVRL
WEDNIRLLEKTSHLEQRVIDLESRNTALELGKTTLEESNSDQARSLELVEREKQALASSLEAKVQALKESEGQKT
LLSDQLTKAQARLAIIEGENKTLEQRKTELERQCSSLQAHVKELDGQKKSLSDDKEALQKQIDLLERHNKTLSDD
KEALHKRAGRLDSQIETLLRDQIKLRDQDRTLLKQKQKLLEQLAFSQGQNLTLGESKIHLKGQIKALSVEKTTLQ
GEITRLQGQVSHLEGTNQLTDSLQQGLSAVNELTKRFEEEKARLQTDNTAAKTKTEGLEAQVDVLKNENTGLK
QRVASLETENKSLCEANSTLVGEKQRLQGSLDVYQQSNEKLQTKFDTLSGQNSALKTLNNQWEKKNDDVLSE
KISTTRELNDVKTELNDVKRDLTDAQAAAQLQTGEAAELRKQVKELTDSVTRSTDRTKAAETASFKSKAELSKV
TIELEQAREKVSKLETQYNCARDFTATLDDLVRKSLEAAQVALPATPVDSLLSASRKRPREASPDRLEGRGAPD
NAAVVVQQILDDLKDIGAKWNYNEITLAELMGHLSILWNDHQARAHWHDFIRSGHQGFFCFYNLVRSGYGP
SSRCKKYVKCAVDNQDMPPCLHLDVSGTTSKRKTGFNIPTDDVDYYEWND

>NcbZIP_4.2

MNDSGNPDHSLAPAVTASRARTSPPRARKSVGSLDKKRERDRRAQQNLRTKRLERIQELEQRIVMLDDKVRK
YHYICDRLSQENKLLRTRQNDIRHLVASWGPEDGLFLEPSFSTEASLVGDTGFLGAVPEPWPTAVISETESAAQ
AMMEFRRGSPEGMAFSPPELPTDSAIPRWSMTPFHDDDDAIMTDCFRLWLQRPDLVESSPESPTPLELLYGS
KRNFLADVIHQALRKCPYLDPERLAVGWLCYYFIKWMICPTEAAYLRLQPWQLPVEEQLQKKHPYFIDLLWFP
GLRANMINKQHQYDLADAFALLTCCAKVRWPWGKNFLEPGEDGQFRMVPEFYDTFTRIEGWGLTDELIMR
YPAIVDGLDIESIRFRIA

>NcbZIP_4.3

MYSYASSSSSSSYASSSSFLPSSSSFPSTYSTNEQSTASPSSQQHEPTKLSSTMITIPRSSSSSSSFSTTTNSSSNSS
NSNFNSNFNNDINNNSKTSTSLSLPHTTTGAPLHQLDPPSPSMSNTDHIHTDSYTSTGTTPSTSTSTPTSTTGIS
TDTSTGTSTPTSDTSTDTSTSTASQRQPQKYHTRRRLQVRRAQSSHRQRKADYIKRLEREIASIRGMIDQMKE
DMEVLRGENEDLGVMAVRR

>ScbZIP_4.1

MQNPPLIRPDMYNQGSSSMATYNASEKNLNEHPSPQIAQPSTSQKLPYRINPTTTNGDTDISVNSNPIQPPLP
NLMHLSGPSDYRSMHQSPIHPSYIIPPHSNERKQSASYNRPQNAHVSIQPSVVFPPKSYSISYAPYQINPPLPN
GLPNQSISLNKEYIAEEQLSTLPSRNTSVTTAPPSFQNSADTAKNSADNNDNNDNVTKPVPDKDTQLISSSGKT
LRNTRRAAQNRTAQKAFRQRKEKYIKNLEQKSKIFDDLLAENNNFKSLNDSLRNDNNILIAQHEAIRNAITMLR
SEYDVLCNENNMLKNENSIIKNEHNMSRNENENLKLENKRFHAEYIRMIEDIENTKRKEQEQRDEIEQLKKKIR
SLEEIVGRHSDSAT

>ScbZIP_4.2

MGNILRKGQQIYLAGDMKKQMLLNKDGTPKRKVGRPGRKRIDSEAKSRRTAQNRAAQRAFRDRKEAKMKS
LQERVELLEQKDAQNKTTTDFLLCSLKSLLSEITKYRAKNSDDERILAFLDDLQEQQKRENEKGTSTAVSKAAKE
LPSPNSDENMTVNTSIEVQPHTQENEKVMWNIGSWNAPSLTNSWDSPPGNRTGAVTIGDESINGSEMPDF
SLDLVSNDRQTGLEALDYDIHNYFPQHSERLTAEKIDTSACQCEIDQKYLPYETEDDTLFPSVLPLAVGSQCNNI
CNRKCIGTKPCSNKEIKCDLITSHLLNQKSLASVLPVAASHTKTIRTQSEAIEHISSAISNGKASCYHILEEISSLPKY
SSLDIDDLCSELIIKAKCTDDCKIVVKARDLQSALVRQLL

>ScbZIP_5.1

MSEYQPSLFALNPMGFSPLDGSKSTNENVSASTSTAKPMVGQLIFDKFIKTEEDPIIKQDTPSNLDFDFALPQT
ATAPDAKTVLPIPELDDAVVESFFSSSTDSTPMFEYENLEDNSKEWTSLFDNDIPVTTDDVSLADKAIESTEEVS
LVPSNLEVSTTSFLPTPVLEDAKLTQTRKVKKPNSVVKKSHHVGKDDESRLDHLGVVAYNRKQRSIPLSPIVPES
SDPAALKRARNTEAARRSRARKLQRMKQLEDKVEELLSKNYHLENEVARLKKLVGER

>ScbZIP_5.2

MDYKHNFATSPDSFLDGRQNPLLYTDFLSSNKELIYKQPSGPGLVDSAYNFHHQNSLHDRSVQENLGPMFQP
FGVDISHLPITNPPIFQSSLPAFDQPVYKRRISISNGQISQLGEDLETVENLYNCQPPILSSKAQQNPNPQQVAN
PSAAIYPSFSSNELQNVPQPHEQATVIPEAAPQTGSKNIYAAMTPYDSNIKLNIPAVAATCDIPSATPSIPSGDS
TMNQAYINMQLRLQAQMQTKAWKNAQLNVHPCTPASNSSVSSSSSCQNINDHNIENQSVHSSISHGVNH
HTVNNSCQNAELNISSSLPYESKCPDVNLTHANSKPQYKDATSALKNNINSEKDVHTAPFSSMHTTATFQIKQ
EARPQKIENNTAGLKDGAKAWKRARLLERNRIAASKCRQRKKMSQLQLQREFDQISKENTMMKKKIENYEKL
VQKMKKISRLHMQECTINGGNNSYQSLQNKDSDVNGFLKMIEEMIRSSSLYDE

>ScbZIP_6.1

MEMTDFELTSNSQSNLAIPTNFKSTLPPRKRAKTKEEKEQRRIERILRNRRAAHQSREKKRLHLQYLERKCSLLE
NLLNSVNLEKLADHEDALTCSHDAFVASLDEYRDFQSTRGASLDTRASSHSSSDTFTPSPLNCTMEPATLSPKS
MRDSASDQETSWELQMFKTENVPESTTLPAVDNNNLFDAVASPLADPLCDDIAGNSLPFDNSIDLDNWRNP
EAQSGLNSFELNDFFITS

>ScbZIP_8.1

MTPSNMDDNTSGFMKFINPQCQEEDCCIRNSLFQEDSKCIKQQPDLLSEQTAPFFPILEDQCPALNLDRSNND
LLLQNNISFPKGSDLQAIQLTPISGDYSTYVMADNNNNDNDSYSNTNYFSKNNGISPSSRSPSVAHNENVPDD
SKAKKKAQNRAAQKAFRERKEARMKELQDKLLESERNRQSLLKEIEELRKANTEINAENRLLLRSGNENFSKDIE
DDTNYKYSFPTKDEFFTSMVLESKLNHKGKYSLKDNEIMKRNTQYTDEAGRHVLTVPATWEYLYKLSEERDFD
VTYVMSKLQGQECCHTHGPAYPRSLIDFLVEEATLNE

>ScbZIP_9.1

MFTGQEYHSVDSNSNKQKDNNKRGIDDTSKILNNKIPHSVSDTSAAATTTSTMNNSALSRSLDPTDINYSTN
MAGVVDQIHDYTTSNRNSLTPQYSIAAGNVNSHDRVVKPSANSNYQQAAYLRQQQQQDQRQQSPSMKTE
EESQLYGDILMNSGVVQDMHQNLATHTNLSQLSSTRKSAPNDSTTAPTNASNIANTASVNKQMYFMNMN
MNNNPHALNDPSILETLSPFFQPFGVDVAHLPMTNPPIFQSSLPGCDEPIRRRRISISNGQISQLGEDIETLENL
HNTQPPPMPNFHNYNGLSQTRNVSNKPVFNQAVPVSSIPQYNAKKVINPTKDSALGDQSVIYSKSQQRNFV
NAPSKNTPAESISDLEGMTTFAPTTGGENRGKSALRESHSNPSFTPKSQGSHLNLAANTQGNPIPGTTAWKR
ARLLERNRIAASKCRQRKKVAQLQLQKEFNEIKDENRILLKKLNYYEKLISKFKKFSKIHLREHEKLNKDSDNNVN

GTNSSNKNESMTVDSLKIIEELLMIDSDVTEVDKDTGKIIAIKHEPYSQRFGSDTDDDDIDLKPVEGGKDPDNQ
SLPNSEKIK

>ScbZIP_9.2

MSAKQGWEKKSTNIDIASRKGMNVNNLSEHLQNLISSDSELGSRLLSLLLVSSGNAEELISMINNGQDVSQFK
KLREPRKGKVAATTAVVVKEEEAPVSTSNELDKIKQERRRKNTEASQRFRIRKKQKNFENMNKLQNLNTQINK
LRDRIEQLNKENEFWKAKLNDINEIKSLKLLNDIKRRNMGR

>ScbZIP_9.3

MALPLIKPKESEESHLALLSKIHVSKNWKLPPRLPHRAAQRRKRVHRLHEDYETEENDEELQKKKRQNRDAQR
AYRERKNNKLQVLEETIESLSKVVKNYETKLNRLQNELQAKESENHALKQKLETLTLKQASVPAQDPILQNLIEN
FKPMKAIPIKYNTAIKRHQHSTELPSSVKCGFCNDNTTCVCKELETDHRKSDDGVATEQKDMSMPHAECNNK
DNPNGLCSNCTNIDKSCIDIRSIIH

>ScbZIP_13.1

MSVSTAKRSLDVVSPGSLAEFEGSKSRHDEIENEHRRTGTRDGEDSEQPKKKGSKTSKKQDLDPETKQKRTAQ
NRAAQRAFRERKERKMKELEKKVQSLESIQQQNEVEATFLRDQLITLVNELKKYRPETRNDSKVLEYLARRDPN
LHFSKNNVNHSNSEPIDTPNDDIQENVKQKMNFTFQYPLDNDNDNDNSKNVGKQLPSPNDPSHSAPMPIN
QTQKKLSDATDSSSATLDSLSNSNDVLNNTPNSSTSMDWLDNVIYTNRFVSGDDGSNSKTKNLDSNMFSND
FNFENQFDEQVSEFCSKMNQVCGTRQCPIPKKPISALDKEVFASSSILSSNSPALTNTWESHSNITDNTPANVI
ATDATKYENSFSGFGRLGFDMSANHYVVNDNSTGSTDSTGSTGNKNKKNNNNSDDVLPFISESPFDMNQVT
NFFSPGSTGIGNNAASNTNPSLLQSSKEDIPFINANLAFPDDNSTNIQLQPFSESQSQNKFDYDMFFRDSSKEG
NNLFGEFLEDDDDDKKAANMSDDESSLIKNQLINEEPELPKQYLQSVPGNESEISQKNGSSLQNADKINNGN
DNDNDNDVVPSKEGSLLRCSEIWDRITTHPKYSDIDVDGLCSELMAKAKCSERGVVINAEDVQLALNKHMN

>ScbZIP_14.1

MSSEERSRQPSTVSTFDLEPNPFEQSFASSKKALSLPGTISHPSLPKELSRNNSTSTITQHSQRSTHSLNSIPEEN
GNSTVTDNSNHNDVKKDSPSFLPGQQRPTIISPPILTPGGSKRLPPLLLSPSILYQANSTTNPSQNSHSVSVSNS
NPSAIGVSSTSGSLYPNSSSPSGTSLIRQPRNSNVTTSNSGNGFPTNDSQMPGFLLNLSKSGLTPNESNIRTGLT
PGILTQSYNYPVLPSINKNTITGSKNVNKSVTVNGSIENHPHVNIMHPTVNGTPLTPGLSSLLNLPSTGVLANPV
FKSTPTTNTTDGTVNNSISNSNFSPNTSTKAAVKMDNPAEFNAIEHSAHNHKENENLTTQIENNDQFNNKTR
KRKRRMSSTSSTSKASRKNSISRKNSAVTTAPAQKDDVENNKISNNVTLDENEEQERKRKEFLERNRVAASKFR
KRKKEYIKKIENDLQFYESEYDDLTQVIGKLCGIIPSSSSNSQFNVNVSTPSSSSPPSTSLIALLESSISRSDYSSAMS
VLSNMKQLICETNFYRRGGKNPRDDMDGQEDSFNKDTNVVKSENAGYPSVNSRPIILDKKYSLNSGANISKS
NTTTNNVGNSAQNIINSCYSVTNPLVINANSDTHDTNKHDVLSTLPHNN

>ScbZIP_15.1

MRQRRSVVAVSVKPKGFKLGHKQGSMSTTSPPPSSPDGNVSTSGPSAIKLSKNWELPQRLKPGRKPKSKRGD
ASANNDGSSKIKKVQTSNQKDQMTTKDHENEGAKGHEGKSDDEGNGSGDENGVDSVEKRRRQNRDAQR
AYRERRTTRIQVLEEKVEMLHNLVDDWQRKYKLLESEFSDTKENLQKSIALNNELQKALPLIVNTPFQQQPEN
PPDNPISILEMVENFKPIGAVSLKKGKLKAHC

>ScbZIP_15.2

MLMQIKMDNHPFNFQPILASHSMTRDSTKPKKMTDTAFVPSPPVGFIKEENKADLHTISVVASNVTLPQIQL
PKIATLEEPGYESRTGSLTDLSGRRNSVNIGALCEDVPNTAGPHIARPVTINNLIPPSLPRLNTYQLRPQLSDTHL
NCHFNSNPYTTASHAPFESSYTTASTFTSQPAASYFPSNSTPATRKNSATTNLPSEERRRVSVSLSEQVFNEGER
YNNDGQLIGKTGKPLRNTKRAAQNRSAQKAFRQRREKYIKNLEEKSKLFDGLMKENSELKKMIESLKSKLKE

>ScbZIP_14.2

MKQEQSHEGDSYSTEFINLFGKDTATHPSSNNGANNNGMGSTNSLDQFVATASSSSSLVTSSENRRPLIGDV
TNRGNTNLYDHAVTPEILLEQLAYVDNFIPSLDNEFSNVDWNVNTTHNNANNNGADTFSSINANPFDLDEQL
AIELSAFADDSFIFPDEDKPSNNNNNSNNGNDDHSNHDVLHEDPSTNNRQRNPHFLTQRRNTFLTSQYDQS
KSRFSSKNKRNGNNGETNNFGDNMQNNHPFEPNFMGSPSQFPADATNMTSIDHGGFTNVDITSTENNTT
GDNGVDALSNLLHRTTHTPNRSSPLSNVTSAQNSSSQQRKHSESKVDSNSDNNSSNKAPNITVPDYSIIPTSVL
VTLLPRVNVPNGAYNSLISAGFDNDQIDAIAAIMAYHHQKKIRENNSNNNKNINTNDSQEAPILKNINELLSVL
IPPSPAETAAPTTLSTSPSFNEHGVVAEASFLSSILELGIKHPKSNNIHNQRQPSRNDHKISRESDGNNGNDNV
HHNNAVIKSSTTRGDEIAKIRSEPTLNASSSDHKENSLKRSHSGDLKNKKVPVDRKYSDNEDDEYDDADLHGFE
KKQLIKKELGDDDEDLLIQSKKSHQKKKLKEKELESSIHELTEIAASLQKRIHTLETENKLLKNLVLSSGETEGIKKA
ESLKKQIFEKVQKE

>ScbZIP_16.1

MAKPRGRKGGRKPSLTPPKNKRAAQLRASQNAFRKRKLERLEELEKKEAQLTVTNDQIHILKKENELLHFMLR
SLLTERNMPSDERNISKACCEEKPPTCNTLDGSVVLSSTYNSLEIQQCYVFFKQLLSVCVGKNCTVPSPLNSFDR
SFYPIGCTNLSNDIPGYSFLNDAMSEIHTFGDFNGELDSTFLEFSGTEIKEPNNFITENTNAIETAAASMVIRQGF
HPRQYYTVDAFGGDVLLSAMDIWSFMKVHPKVNTFDLEILGTELKKSATCSNFDILISLKHFIKVFSSKL

>ScbZIP_8.2

MSVSTAKRSLDVVSPGSLAEFEGSKSRHDEIENEHRRTGTRDGEDSEQPKKKGSKTSKKQDLDPETKQKRTAQ
NRAAQRAFRERKERKMKELEKKVQSLESIQQQNEVEATFLRDQLITLVNELKKYRPETRNDSKVLEYLARRDPN
LHFSKNNVNHSNSEPIDTPNDDIQENVKQKMNFTFQYPLDNDNDNDNSKNVGKQLPSPNDPSHSAPMPIN
QTQKKLSDATDSSSATLDSLSNSNDVLNNTPNSSTSMDWLDNVIYTNRFVSGDDGSNSKTKNLDSNMFSND
FNFENQFDEQVSEFCSKMNQVCGTRQCPIPKKPISALDKEVFASSSILSSNSPALTNTWESHSNITDNTPANVI
ATDATKYENSFSGFGRLGFDMSANHYVVNDNSTGSTDSTGSTGNKNKKNNNNSDDVLPFISESPFDMNQVT
NFFSPGSTGIGNNAASNTNPSLLQSSKEDIPFINANLAFPDDNSTNIQLQPFSESQSQNKFDYDMFFRDSSKEG
NNLFGEFLEDDDDDKKAANMSDDESSLIKNQLINEEPELPKQYLQSVPGNESEISQKNGSSLQNADKINNGN
DNDNDNDVVPSKEGSLLRCSEIWDRITTHPKYSDIDVDGLCSELMAKAKCSERGVVINAEDVQLALNKHMN

>ScbZIP_8.3

MTPSNMDDNTSGFMKFINPQCQEEDCCIRNSLFQEDSKCIKQQPDLLSEQTAPFPILEDQCPALNLDRSNND
LLLQNNISFPKGSDLQAIQLTPISGDYSTYVMADNNNNDNDSYSNTNYFSKNNGISPSSRSPSVAHNENVPDD
SKAKKKAQNRAAQKAFRERKEARMKELQDKLLESERNRQSLLKEIEELRKANTEINAENRLLLRSGNENFSKDIE
DDTNYKYSFPTKDEFFTSMVLESKLNHKGKYSLKDNEIMKRNTQYTDEAGRHVLTVPATWEYLYKLSEERDFD
VTYVMSKLQGQECCHTHGPAYPRSLIDFLVEEATLNE

>ScbZIP_5.3

MSEYQPSLFALNPMGFSPLDGSKSTNENVSASTSTAKPMVGQLIFDKFIKTEEDPIIKQDTPSNLDFDFALPQT
ATAPDAKTVLPIPELDDAVVESFFSSSTDSTPMFEYENLEDNSKEWTSLFDNDIPVTTDDVSLADKAIESTEEVS

LVPSNLEVSTTSFLPTPVLEDAKLTQTRKVKKPNSVVKKSHHVGKDDESRLDHLGVVAYNRKQRSIPLSPIVPES
SDPAALKRARNTEAARRSRARKLQRMKQLEDKVEELLSKNYHLENEVARLKKLVGER

>ScbZIP_5.4

MDYKHNFATSPDSFLDGRQNPLLYTDFLSSNKELIYKQPSGPGLVDSAYNFHHQNSLHDRSVQENLGPMFQP
FGVDISHLPITNPPIFQSSLPAFDQPVYKRRISISNGQISQLGEDLETVENLYNCQPPILSSKAQQNPNPQQVAN
PSAAIYPSFSSNELQNVPQPHEQATVIPEAAPQTGSKNIYAAMTPYDSNIKLNIPAVAATCDIPSATPSIPSGDS
TMNQAYINMQLRLQAQMQTKAWKNAQLNVHPCTPASNSSVSSSSSCQNINDHNIENQSVHSSISHGVNH
HTVNNSCQNAELNISSSLPYESKCPDVNLTHANSKPQYKDATSALKNNINSEKDVHTAPFSSMHTTATFQIKQ
EARPQKIENNTAGLKDGAKAWKRARLLERNRIAASKCRQRKKMSQLQLQREFDQISKENTMMKKKIENYEKL
VQKMKKISRLHMQECTINGGNNSYQSLQNKDSDVNGFLKMIEEMIRSSSLYDE

>ScbZIP_15.3

MRQRRSVVAVSVKPKGFKLGHKQGSMSTTSPPPSSPDGNVSTSGPSAIKLSKNWELPQRLKPGRKPKSKRGD
ASANNDGSSKIKKVQTSNQKDQMTTKDHENEGAKGHEGKSDDEGNGSGDENGVDSVEKRRRQNRDAQR
AYRERRTTRIQVLEEKVEMLHNLVDDWQRKYKLLESEFSDTKENLQKSIALNNELQKALPLIVNTPFQQQPEN
PPDNPISILEMVENFKPIGAVSLKKGKLKAHC

>ScbZIP_14.3

MSSEERSRQPSTVSTFDLEPNPFEQSFASSKKALSLPGTISHPSLPKELSRNNSTSTITQHSQRSTHSLNSIPEEN
GNSTVTDNSNHNDVKKDSPSFLPGQQRPTIISPPILTPGGSKRLPPLLLSPSILYQANSTTNPSQNSHSVSVSNS
NPSAIGVSSTSGSLYPNSSSPSGTSLIRQPRNSNVTTSNSGNGFPTNDSQMPGFLLNLSKSGLTPNESNIRTGLT
PGILTQSYNYPVLPSINKNTITGSKNVNKSVTVNGSIENHPHVNIMHPTVNGTPLTPGLSSLLNLPSTGVLANPV
FKSTPTTNTTDGTVNNSISNSNFSPNTSTKAAVKMDNPAEFNAIEHSAHNHKENENLTTQIENNDQFNNKTR
KRKRRMSSTSSTSKASRKNSISRKNSAVTTAPAQKDDVENNKISNNVTLDENEEQERKRKEFLERNRVAASKFR
KRKKEYIKKIENDLQFYESEYDDLTQVIGKLCGIIPSSSSNSQFNVNVSTPSSSSPPSTSLIALLESSISRSDYSSAMS
VLSNMKQLICETNFYRRGGKNPRDDMDGQEDSFNKDTNVVKSENAGYPSVNSRPIILDKKYSLNSGANISKS
NTTTNNVGNSAQNIINSCYSVTNPLVINANSDTHDTNKHDVLSTLPHNN

>ScbZIP_9.4

MFTGQEYHSVDSNSNKQKDNNKRGIDDTSKILNNKIPHSVSDTSAAATTTSTMNNSALSRSLDPTDINYSTN
MAGVVDQIHDYTTSNRNSLTPQYSIAAGNVNSHDRVVKPSANSNYQQAAYLRQQQQQDQRQQSPSMKTE
EESQLYGDILMNSGVVQDMHQNLATHTNLSQLSSTRKSAPNDSTTAPTNASNIANTASVNKQMYFMNMN
MNNNPHALNDPSILETLSPFFQPFGVDVAHLPMTNPPIFQSSLPGCDEPIRRRRISISNGQISQLGEDIETLENL
HNTQPPPMPNFHNYNGLSQTRNVSNKPVFNQAVPVSSIPQYNAKKVINPTKDSALGDQSVIYSKSQQRNFV
NAPSKNTPAESISDLEGMTTFAPTTGGENRGKSALRESHSNPSFTPKSQGSHLNLAANTQGNPIPGTTAWKR
ARLLERNRIAASKCRQRKKVAQLQLQKEFNEIKDENRILLKKLNYYEKLISKFKKFSKIHLREHEKLNKDSDNNVN
GTNSSNKNESMTVDSLKIIEELLMIDSDVTEVDKDTGKIIAIKHEPYSQRFGSDTDDDDIDLKPVEGGKDPDNQ
SLPNSEKIK

>ScbZIP_9.5

MSAKQGWEKKSTNIDIASRKGMNVNNLSEHLQNLISSDSELGSRLLSLLLVSSGNAEELISMINNGQDVSQFK
KLREPRKGKVAATTAVVVKEEEAPVSTSNELDKIKQERRRKNTEASQRFRIRKKQKNFENMNKLQNLNTQINK
LRDRIEQLNKENEFWKAKLNDINEIKSLKLLNDIKRRNMGR

>ScbZIP_9.6

MALPLIKPKESEESHLALLSKIHVSKNWKLPPRLPHRAAQRRKRVHRLHEDYETEENDEELQKKKRQNRDAQR
AYRERKNNKLQVLEETIESLSKVVKNYETKLNRLQNELQAKESENHALKQKLETLTLKQASVPAQDPILQNLIEN
FKPMKAIPIKYNTAIKRHQHSTELPSSVKCGFCNDNTTCVCKELETDHRKSDDGVATEQKDMSMPHAECNNK
DNPNGLCSNCTNIDKSCIDIRSIIH

>ScbZIP_9.7

MQNPPLIRPDMYNQGSSSMATYNASEKNLNEHPSPQIAQPSTSQKLPYRINPTTTNGDTDISVNSNPIQPPLP
NLMHLSGPSDYRSMHQSPIHPSYIIPPHSNERKQSASYNRPQNAHVSIQPSVVFPPKSYSISYAPYQINPPLPN
GLPNQSISLNKEYIAEEQLSTLPSRNTSVTTAPPSFQNSADTAKNSADNNDNNDNVTKPVPDKDTQLISSSGKT
LRNTRRAAQNRTAQKAFRQRKEKYIKNLEQKSKIFDDLLAENNNFKSLNDSLRNDNNILIAQHEAIRNAITMLR
SEYDVLCNENNMLKNENSIIKNEHNMSRNENENLKLENKRFHAEYIRMIEDIENTKRKEQEQRDEIEQLKKKIR
SLEEIVGRHSDSAT

>ScbZIP_9.8

MGNILRKGQQIYLAGDMKKQMLLNKDGTPKRKVGRPGRKRIDSEAKSRRTAQNRAAQRAFRDRKEAKMKS
LQERVELLEQKDAQNKTTTDFLLCSLKSLLSEITKYRAKNSDDERILAFLDDLQEQQKRENEKGTSTAVSKAAKE
LPSPNSDENMTVNTSIEVQPHTQENEKVMWNIGSWNAPSLTNSWDSPPGNRTGAVTIGDESINGSEMPDF
SLDLVSNDRQTGLEALDYDIHNYFPQHSERLTAEKIDTSACQCEIDQKYLPYETEDDTLFPSVLPLAVGSQCNNI
CNRKCIGTKPCSNKEIKCDLITSHLLNQKSLASVLPVAASHTKTIRTQSEAIEHISSAISNGKASCYHILEEISSLPKY
SSLDIDDLCSELIIKAKCTDDCKIVVKARDLQSALVRQLL

>ScbZIP_6.2

MEMTDFELTSNSQSNLAIPTNFKSTLPPRKRAKTKEEKEQRRIERILRNRRAAHQSREKKRLHLQYLERKCSLLE
NLLNSVNLEKLADHEDALTCSHDAFVASLDEYRDFQSTRGASLDTRASSHSSSDTFTPSPLNCTMEPATLSPKS
MRDSASDQETSWELQMFKTENVPESTTLPAVDNNNLFDAVASPLADPLCDDIAGNSLPFDNSIDLDNWRNP
EAQSGLNSFELNDFFITS

>SpbZIP_1.1

MSGQTETLSSTSNIPIAKAEPEQSADFSASHKKRGPVSDRSSRRTSSEEVDLMPNVDDEVDGDVKPKKIGRKN
SDQEPSSKRKAQNRAAQRAFRKRKEDHLKALETQVVTLKELHSSTTLENDQLRQKVRQLEEELRILKDGSFTFE
MSLPHRNPSLSSLPTTGFSSNFAHMKDGISPQSNLHLSPNSIEKPNMHQNVLHNDRSADNLNHRYQVPPTLV
DSNSAQGTLSPETPSSSDSPSNLYLNYPKRKSITHLHHDCSALSNGENGEDVADGKQFCQKLSTACGSIACSML
TKTTPHRASVDILSNLHESTVSPPMADESVQRSSEVSKSIPNVELSLNVNQQFVSPFGGTDSFPLPTDTGLDSLF
EPDSAIENSHLKNVVMEPELFQAWREPAESLDKEFFNDEGEIDDVFHNYFHNSNENGDLITNSLHGLDFLENA
NESFPEQMYPFIKHNKDYISNHPDEVPPDGLPQKGKHDTSSQMPSENEIVPAKERAYLSCPKVWSKIINHPRF
ESFDIDDLCSKLKNKAKCSSSGVLLDERDVEAALNQFN

>SpbZIP_1.2

MTAKKKEVDDEKRRRILERNRIAASKFRQKKKEWIKELEQTANAAFEQSKRLQLLLSQLQQEAFRLKSQLLAHQ
GCQCSVKIRSVLTDFQTAHNALHSQHMAYRPVQPPPGDNMLESVVSVSPTQMHPSLQGLPPNQHPQMPP
SSQQPNSDDVQQHMFSAAGLPRSLGGPI

>SpbZIP_1.3

MTYETNTPTEESIIPKHEDGEEYNSIYLSRFEKDISISQTLDFSQFMQTQILLTAKRKALELGDDRSPINNDPYNIR
RSDFDELSEYTASKSPSIISEASHNSPSRELDDSGDENTSKLTGTKQSMLKARNRQAAQKCRIKKKKYLQTLQD
QVNYYTSENKELLQSANDLREEIIKLRTLVFAHRDCPVSKACSKALFLMGKEKPLTPP

>SpbZIP_1.4

MDFTPNSAINHLNLKFDDVPVSDDFSKDDLAEQLNVFTNPYFLDLEPSSMLSEGYYGFVSQPSGSSNSNKQEK
NVQQQNPEKISTLQQVKEEEVSNTFSAPLNATGNFSSANPASIDLAYLDLQKLLTLPDHSKETQEKTSSQRELF
EQKSSVASASKDNVSSSSILQGSASSKLLPDQSARQHQVLVGQTAIPTSEASSSINNTPLQAPVSSFADQNAFT
NPLSTFASPDLASVSSPSLSSYKGAQSPNANSKRTKATSAIRTAAEEDKRRRNTAASARFRIKKKLKEQQLERTA
KELTEKVAILETRVRELEMENNWLKGLIRPTSNF

>SpbZIP_2.1

MSPSPVNTSTEPASVAAVSNGNATASSTQVPENNQSDSFAPPSNNSQQNQQSSTIAPNGGAGSVANANPA
DQSDGVTPSFVGSLKLDYEPNPFEHSFGSTASVGQGNPSLNRNPSLSNIPSGVPPAFARTLLPPVSSIASPDILS
GAPGIASPLGYPAWSAFTRGTMHNPLSPAIYDATLRPDYLNNPSDASAAARFSSGTGFTPGVNEPFRSLLTPT
GAGFPAPSPGTANLLGFHTFDSQFPDQYRFTPRDGKPPVVNGTNGDQSDYFGANAAVHGLCLLSQVPDQQ
QKLQQPISSENDQAASTTANNLLKQTQQQTFPDSIRPSFTQNTNPQAVTGTMNPQASRTQQQPMYFMGS
QQFNGMPSVYGDTVNPADPSLTLRQTTDFSGQNAENGSTNLPQKTSNSDMPTANSMPVKLENGTDYSTSQ
EPSSNANNQSSPTSSINGKASSESANGTSYSKGSSRRNSKNETDEEKRKSFLERNRQAALKCRQRKKQWLSNL
QAKVEFYGNENEILSAQVSALREEIVSLKTLLIAHKDCPVAKSNSAAVATSVIGSGDLAQRINLGY

>SpbZIP_2.2

MDFANVYLGLDTLGNEGYGASVSNGTHHDFHFNFNTFRDGGNHEVGEKRAESSDIGVYECRGPVQGPKDQ
TSFPSNSHNNYASIVGDASIGHYLKGPERTSEVSLPQTVNLSEISNNNDKGQPTNTPPVRSTIVAPSLYSEGSTL
NHYNNGSHQVLHPQFQNGTNAPYVVQSNLMQNNVNLTGAEQEKGLDLYKNSATANNDEIFYNLESLRREG
YLNSNKKQSQSPNGDYNSSDESCSNKTVASSQRRGTPGSNNVHTASNNETPDMKRRRFLERNRIAASKCRQ
KKKLWTQNLEKTAHIACEQSKALRILVSQLREEVICLKNQLLAHQDCNCEGIRQYLSSEAQGIMSFQKH

>SpbZIP_1.5

MTAKKKEVDDEKRRRILERNRIAASKFRQKKKEWIKELEQTANAAFEQSKRLQLLLSQLQQEAFRLKSQLLAHQ
GCQCSVKIRSVLTDFQTAHNALHSQHMAYRPVQPPPGDNMLESVVSVSPTQMHPSLQGLPPNQHPQMPP
SSQQPNSDDVQQHMFSAAGLPRSLGGPI

>SpbZIP_1.6

MDFTPNSAINHLNLKFDDVPVSDDFSKDDLAEQLNVFTNPYFLDLEPSSMLSEGYYGFVSQPSGSSNSNKQEK
NVQQQNPEKISTLQQVKEEEVSNTFSAPLNATGNFSSANPASIDLAYLDLQKLLTLPDHSKETQEKTSSQRELF
EQKSSVASASKDNVSSSSILQGSASSKLLPDQSARQHQVLVGQTAIPTSEASSSINNTPLQAPVSSFADQNAFT
NPLSTFASPDLASVSSPSLSSYKGAQSPNANSKRTKATSAIRTAAEEDKRRRNTAASARFRIKKKLKEQQLERTA
KELTEKVAILETRVRELEMENNWLKGLIRPTSNF

>SpbZIP_1.7

MSGQTETLSSTSNIPIAKAEPEQSADFSASHKKRGPVSDRSSRRTSSEEVDLMPNVDDEVDGDVKPKKIGRKN
SDQEPSSKRKAQNRAAQRAFRKRKEDHLKALETQVVTLKELHSSTTLENDQLRQKVRQLEEELRILKDGSFTFE
MSLPHRNPSLSSLPTTGFSSNFAHMKDGISPQSNLHLSPNSIEKPNMHQNVLHNDRSADNLNHRYQVPPTLV

DSNSAQGTLSPETPSSSDSPSNLYLNYPKRKSITHLHHDCSALSNGENGEDVADGKQFCQKLSTACGSIACSML
TKTTPHRASVDILSNLHESTVSPPMADESVQRSSEVSKSIPNVELSLNVNQQFVSPFGGTDSFPLPTDTGLDSLF
EPDSAIENSHLKNVVMEPELFQAWREPAESLDKEFFNDEGEIDDVFHNYFHNSNENGDLITNSLHGLDFLENA
NESFPEQMYPFIKHNKDYISNHPDEVPPDGLPQKGKHDTSSQMPSENEIVPAKERAYLSCPKVWSKIINHPRF
ESFDIDDLCSKLKNKAKCSSSGVLLDERDVEAALNQFN

>SpbZIP_2.3

MDFANVYLGLDTLGNEGYGASVSNGTHHDFHFNFNTFRDGGNHEVGEKRAESSDIGVYECRGPVQGPKDQ
TSFPSNSHNNYASIVGDASIGHYLKGPERTSEVSLPQTVNLSEISNNNDKGQPTNTPPVRSTIVAPSLYSEGSTL
NHYNNGSHQVLHPQFQNGTNAPYVVQSNLMQNNVNLTGAEQEKGLDLYKNSATANNDEIFYNLESLRREG
YLNSNKKQSQSPNGDYNSSDESCSNKTVASSQRRGTPGSNNVHTASNNETPDMKRRRFLERNRIAASKCRQ
KKKLWTQNLEKTAHIACEQSKALRILVSQLREEVICLKNQLLAHQDCNCEGIRQYLSSEAQGIMSFQKH

>SpbZIP_2.4

MSPSPVNTSTEPASVAAVSNGNATASSTQVPENNQSDSFAPPSNNSQQNQQSSTIAPNGGAGSVANANPA
DQSDGVTPSFVGSLKLDYEPNPFEHSFGSTASVGQGNPSLNRNPSLSNIPSGVPPAFARTLLPPVSSIASPDILS
GAPGIASPLGYPAWSAFTRGTMHNPLSPAIYDATLRPDYLNNPSDASAAARFSSGTGFTPGVNEPFRSLLTPT
GAGFPAPSPGTANLLGFHTFDSQFPDQYRFTPRDGKPPVVNGTNGDQSDYFGANAAVHGLCLLSQVPDQQ
QKLQQPISSENDQAASTTANNLLKQTQQQTFPDSIRPSFTQNTNPQAVTGTMNPQASRTQQQPMYFMGS
QQFNGMPSVYGDTVNPADPSLTLRQTTDFSGQNAENGSTNLPQKTSNSDMPTANSMPVKLENGTDYSTSQ
EPSSNANNQSSPTSSINGKASSESANGTSYSKGSSRRNSKNETDEEKRKSFLERNRQAALKCRQRKKQWLSNL
QAKVEFYGNENEILSAQVSALREEIVSLKTLLIAHKDCPVAKSNSAAVATSVIGSGDLAQRINLGY

>SsbZIP_U.1

MKAESISSTPQVGCLDPLELIDYTEYETVSYHSPSLSPSSSKSQFVPTSVRSSVVSTPTTLPSSDQPTLSGPSHQY
DLYRQQTGIPQGAIANTLAVNENNGHINRYNFQDTSYFSAMSPSEEFVDFGSAPSRTPFHPSDVEMDQDPAF
FYPEQNFVDPSNIGSQNLPVGNVLPAQSSHVGRLWPGMHQQQAALAKAQAQQKQQQAIIAQQRQNAMA
ANGQQRQPQQPRSRAHTGTDPIVEEKISQLLKSMRQGSVATDGDEGNSQNLTHVHRMRKEEEDMDEDER
LLASEEGKKLSSKERRQLRNKVSARAFRSRRKEYISQLEGEIAVKVNENMDLKSQNRALMEENTRLSDLTRMLL
SSPAFSGFLDTLSSNPAAQQAPPPPQQAPIQHEQPIQVRKDVNPYAAQQQIQQQQHIGMVMIPEQAMDFA
MLDINADGFGYQPQVYSVLSLPELIIDSSILSGKSSDLITPLESDDVKVELPIVERKLVHELAPSAVVEETIDEEFDA
DPAFALFADDTTSSTPSPTTHSNFDFDFSTLNLNKPSHFELIIHDIENEKLISSNAFDRASKLCNNLDSLMERLEA
MFL

>SsbZIP_U.2

MAAFPMHSYYRQSSLSVDTQPHNYFEEDEGSILDDNILDHSALDSGLEMSPPMDNSRRESFAVSSTLFSPKSD
EWQHVEMQPMASNNPFVEHNSNNPFMRIEAAQNGTYGHSNHGWGMGNSSGMSTPMQGHDGLPSEFE
VNVPIFQRPVQTPFTNPGNHQLPLFSANANGSSHPSPQKDWSVSEMDHRIPKRMRPHSPTLRSHPEMSRR
GDGIRKKNARFDIPAERNLTNIDQLISQSTDEQEIKELKQQKRLLRNRQAALDSRQRKKQHTERLEDEKKHYTA
LINDLEEDLAEAKLALDEWARKEQHYQQYIESLQMEKEEMVRTHTLETGDLRKKVSVLTEHMQKMESTAMS
TVPSSTGFSADYSDIDGLNMDGNWDNISFLNDFSMESEIKVENSLVPTKKVDTSLLSEPEKPAAQGLLLMLLLV
GAFVASKGTSPSIPRMSDDVRAASATLLEDVFKDAGIQQSASGVSVSDVTNAIAPLPSGTSWSTTQNPSMGG
NEMVGVTSSTLGDLANSLAQPTEDQNNEQLFSLSAAQYNGLTSQDFLDNSPATRSTSQGRKNLLESLAAMRS
NSKKSAAEVYTRSLLWDQVPSEVVRNFAKLVSECNSRNGPEECDTSSG

>SsbZIP_U.3

MTVTQIYPSIAPLPITESDLLYHPQHFDPQSQTLFQQQQEQQQQQHSYLDPALAQHDLRIFTQGLAHNPSYD
TPLAPYPASDSSDGSMDFCGGAMNWINGRSANMPTDTYLYNQTSPSAPPYALALTSPTQSLSPREPNAKRD
ASTAASARFRQKKKQREKILELNVKQQQERIGMMECRIKELEGENGFLKELVMGRIKWKEEKGKEESKRDGN
EEEKDNGETE

>SsbZIP_U.4

MSTCGDVLEHFQPYDYANQYNSRVPQTTTQQKPPIVQPMYSEMPTDMIDDAMRRGSNSDDEENLTPAQS
RRKAQNRQAQRAFRERKERHVKELEAKLAELEKNTGDLAQENERLKLKLAKAATENEILKATSGHSSRGGSEPL
ANTGPMKYTPTDFYTEVLYAHENKVPSHRIVLGDKGERLLAAGLVDVGIISEMLKGAAKCDGQGPVFDESVIL
DAIEKSVAAGSDELL

>SsbZIP_U.5

MAISAPIWYSPPSPIPLTFDQDTSCDLGTNAFESAFPYTTCTIPQTESIYTIAPYPSPYFDAIDTSMVPELDQSIST
ENNRASFESERNEMNEEMSIETKRRQYRASRDRKPKSLGSRTRETSHDSADSVTEKTPAQKMRRLVQNRVSQ
RNFRKRQANERQRLEDQVKMLSTELHNLSDMYQDLLARYEQAQREKSSSSELIGGWADGVRTLNQFNLGGL
E

>SsbZIP_U.6

MASPTVSTPSSASSPAALAPAPPVLAIKTQNLSIMSAMASASGASTPTIMTTKEWVIPPRPKPGRKPATDTPPT
KRKAQNRAAQRAFRERRAARVGELEEQLEEAKEDQQRRENDMRLKITRLEAEVTRFNGELQSWRLRCETLDR
IAEYEKREKEAAFKELAYLRNGFQSTSTDAVPLPPRRNRQSTQTLPTLPQQQQQQQQSPFVLELEIPSEIGCGG
CKSTNECACVEQVIAMSTQGCGNCGPDTHCQCLEESLKDASTDLKRAHSPTIDATLEKRARLSSRPSTPLEIDF
TAQFSRRQPVIREPSLPPQILTRPVEPCGFCDQATFCPCAERELENRLAPLLNEVTPPPSDTDIENTQVKLPSMQ
PNHMHRPVPTATTSNSCANGPGTCKQCMSDPKAGLFCRSLAAMRASSNSAPPDGCCGGNSSGGGCCKTM
EPASSSEPPPSLSVADTYKTLSTHKNFDQASDELNTWLGRLHATPLQHAGRAPMEVEAASVMGVLKLFDRRF
GRG

>SsbZIP_U.7

MLSSAADSIISDATVKPVLLETSRPLPNPPVPSRNEVSDYIAKRCNKLEEEENPFDSQFGGGPAKLNPAKTPGG
TLLPSIQALQSGSSPGFSNMEGLRSGPLSPNMLAGPKEPADDYFSGDHNMIEGPRGFTPMESALRHERILPSP
GNVLSPGNPYSLNSSSLMGGIASPGIGMYSSGIVATPGTADFQRTASEIRQRQVAASLNEQKRDMNITSQPQ
QLTEGNRNDMSNYPGGGDYTAAQSLHMLANQASASSRDASPHYNIALGQPRQAQASTQKLLLNRNQQTL
NQQMNPMNPMQVNGQMAGRSNDPSVMNGHPMDSRDRNNSVNSNSSSPVSNGNSIGYENDMNGNGS
QSKKRATTKRKASANKTPPKNNKRTKGGSNASKRDDDMKNEEFTNEDLDNMDDQGDFEENEDNNNNGKP
KKPETDDEKRKSFLERNRVAALKCRQRKKQWLNNLQQKVDTYTNENEALQQRIQQMGHEIIQLRTMLLAHK
DTPIGIQQGIAAFVPQIYEEMPQMHQAQNPYGMAGMQPSQNPAMQVQVPDSIQFTIKMCIHGGGGFGSE
TWLGFAHGRVW

>SsbZIP_U.8

MDSSADKEERKREYNRLAQREFRRRRKEHLKNLEQAQKEQSSEQSEEIERLRYQNDELRRENEALRAQIYGST
SQGLLLQPLGPISSDHRQYSLSPSISGASISNASSPPASLSSDMMSMGSMPMTTAMISPSTISMAMLPYDRKK

SRTQIRELFHPLLSDPSILNGSSPDRHLALLHSMVDILPPTLKPNKSQLATAHYYAIDMIASPSLRDRLMTLTQD
VTQSFIRDFGSCIGEAEDIGQIIIWGEDPYNEMAWEISQPLLERWGWLLGRGFVDRSNAWRIQRGALPLPEW

>SsbZIP_U.9

MTSPSPENFDYTNRSDRGDYASEQSEGARSPLSMSLGFLKTLTERKSTRVDGSTPKRRGPKPDSKPALTRRQE
LNRQAQRTHRERKELYIKALEQEVLRLKENFSNVTQSKQSLAEENRQLKQLLAQHGIQWDGSGGFDDFTNTQ
SLGFRSSDSPDSYVPTSSSTFSPPPLSHSSNSNPNSMNYDTESPQIGGANGVYNGNDRSMAQQQVRGVDYD
QAGIDFVLTLERPCMDHMQFLVERSSDVDGEFFSGHALMATCPPESDMDMHPHIPFGHCMPHNHNHETD
GTDGGPPKQKTWDLSKSDLANLLDLSKRLDLNGEITPVMAWGMVLAHPRFLELKEEDFKCMSAELLPKVRCY
GFGAALEEFEVRDALEALFGAELVHMSSI

>SsbZIP_U.10

MDQTKFNDQHPGQLKFDEFLTEDALKMMRYTSNKLSLNFFLTPGTEKGQPHWDSLNNVGNDQAFGADLNI
TDDSFLRIFDDVIEIPDILANEQQQNQLGGSPSVAMKDFRADANTDFPTTPSLNQNNLITNVSENSLPIDNSHL
SSAPQTSNDSKVPSKDTPFTMEKAHSQTMQINPQQSPEQEKANASPHLKEKPSSLSTASTPLRPKKKRRPRAK
KVLTPAETLMVREKYLEKNRRAARKCRLKKKAEMAADQAKYDHYVSELRATKKQLEDSRKELLELIAVCKGMV
GEGCGDGVIRRFVANWERREEACRGMLESGDMGVEAWKLVEKCRSARLGEGVLGDVGLDGGGEAAPFW
QDSNALGLKTQGSALGNEVVMGQGNTDPVNATAAEDSCYLSSGLTDCDETAPYSFCQHSPLTQQTQLPENP
RQHSRQHSRQQLQNINWHHFVSSFQEPSASIPTTGFTNSNHSETSFQSPYSATQATQSMTSPLTSSPTSPFQS
HQEGQIIPTSLTISTSETNSPNSQSSPTSLNKPNSQNLQQRSSSIKIAPRLRHESLDSGYGSICSTTTHQSNSNSNS
NSLSINSASVSSSQNPSSTNSNCWSPSSYLYASEINPTNTLHDSCMNFPPENAEINTINLPIICNVNVNAKTNAN
QNYEETLSNSSTNPSSSSFGALVTDSEFGYNEIADAGMDTHTTATTTAQNNSSNHPPTTNDMDIDKDPSKSIII
VS

>SsbZIP_U.11

MAATSTNRHNPINSDFTLSPRQEELLFAALNSNKSSTKPSNNMDKSLPPSTNALSNTSTSTFNESPIQAPGSGT
LSTFDESPFIDYDYEFEGDDSFNFDFGNDSQGQMIGNLPGSSSDGDADNHEKRSHPDDDDEEEGGGKRREG
DDKSSKKPGRKPLTSEPTSKRKAQNRAAQRAFRERKEKHLKDLETKVDDLQKASESANHENSILRAQIERMTM
ELREYKKRLSLNGGINRSPNGNPPAYFAGKGLSNASATPNDVNFQFEFPRFGRLPGPPITNGSSSMAPSASPTT
PTQNAQSPTDKSQSSSRNHSNASNTFGVGTTQTSAQNGDDMTSFSGLFSPEILDSAFKSSPFDSFGNLTHGS
MSSTGSPHQSTNGQNTSYSSPSASSQSNHGTSSSCGTSPEPTNMQSPYNKATDNTLTTIGEENTCGNSNAAP
GEMSFCEKLNMACGNPNNPIPRALSESGTNPGNVEPLVFDVNGIDWLAQQNNNQFDPQLYGDYREPQNNI
LSNDLYGLDDGFFTDAFDIQDFNSPFNVPTPTTTAKDLVQQIDEKQNEDSEVVPGEDRSTMLSCNTIWDRLQ
NCPKVREGEFDLDSLCKDLQKKAKCSETGAVVNEADFQKIMNSYAPKGCDTMFPTTAKKA

>SsbZIP_U.12

MGLIPVELDIEGIYEDHDRRRRKNGNEKVVSPHVHSRRRAQNRASQRAFRDRKEKHMRELEQRLEELEGRHS
ALSRSYESLQVEYSGVKQELDRLRKVKTNPERSASPSPREYAQSNLKAWDESKVEIMDPLLFDVSAFCFDQDET
GPQHKEQVV

>SsbZIP_U.13

MAYNGRRGPNVSEYIANLNAIPTPQDLQNSNQESFNVDDDLAMFTNAQFFDFDLNQNTTTDLQAPNFDGV
GAQSTADSVDMDLKDLDFGITADFNFSDFNTYPTNFGSHDGMPPVIHPIQTNHQIYQPPSSAGSPTSALVSP

RVGEKRKAESVSDGRSPDFEDASRLAAEEDKRRRNTAASARFRVKKKQREQALEQSAKAMSDKVAALEGRIN QLETENKWLKNLITEKNESKEDIAALWKKYNKDAGDRKGAERKDGVGTEA

>SsbZIP_U.14

MPNFRVIIVGAGPVGLTTAHALSKAGIDFVVLERRQVVVQDVGASLVLAPQSLRVMSQLELLDQLTAISTELV HATSYTMDGKKFKEAWPFEVMKQNQTRQLMRSLTLKTSPEDTVNDEEPFTTEYRTMWCTFPKPEDSEIGLG STSDGSNASVQYLTSKDRAWIFVYEHLEMPTKKRAWYSQEDVEAFAAEHAEMPINDHLKVKDVFAKKYHAG MANLEEGIIEHWSWGRIVLVGDAAHKFTPNHGQGLNNGIQDAVVLVNELHRCIESVGASNQPSKEELSIAFQ RYQSTRTEYVKADYNLSATVTRMSAWPNFCEFCGVISRRRAQNRASQRAFRDRKEKYMRELEQRLRELEGRY NVLSRLYESLQLEVTSVKQELDRMGKDNSRVESSTRNCQVREWEESKIEILDPFLFHVSAFCFVEDHGQGWKE

>SsbZIP_U.15

MSTPNLKFEQSPAESLAESFTSTPGQQYTSLFHPHQSMEPMQALTPQSFDDDSMFGDDMTGEMGGSLAGT PAPEKKPVKKRKSWGQQLPEPKTNLPPRKRAKTEDEKEQRRVERVLRNRRAAQTSRERKRQEVEALEAQKQ QIERRNRDLELQLANMASKYEAVRRKLEQFTGMTGENITSTFTTPSVNSTPNRSETFHAKSPIALTKGLFESEDS SIQPMMTPIRDTHNTMQSAMGTVDLASVSPPPMESTDDGSFRASSFDQTFFNERVGPIADAFSNNFDNLAG DNIGAANDALFEDFIHQDEITQSAPEVHSSDSFIEKTFSLQSQHGASSSGCDVGGNAVIV

>SsbZIP_U.16

MSESGGDDADGRKGGKRELSQSKRAAQNRAAQRAFRQRKEGYIKKLEEQVRDFHALEDNYKAIHAENYSLR EYIIRLQSRLIESQGEFPQPPPNINLSHTSTHPHPQPPQQRQMEPLEHMHRHEPPIAPMAPMGALQPAAART LAAASLKHPGEEQQGQQQQQSYPPHQENKRFKDDDTNDENLIRSQLGGGQLTNDGLPTSMSM

>SsbZIP_U.17

MSRVEKRKLNTMAARRYRQKRVDQMSSLEAALREVERERDALKVRVAKLEGETDILKSLLSKKD

>UmbZIP_8.1

MPLIMRPPAKTSNDMVDPERLRQTARRKEQNRNAQRRLRVRREEHILQLQAQLEELHRRSQSQEEESHFLRE ALALMRAENRTLAEQIAMIHQAFPSTQSSHPLQQRASIRPGADQNASLNFSRSRSQSVTASMIPPFAFGNPA APWSTSTQQPTPAVQQPFGATDAPSATHRTTFSLASPLPGAPGFVGGLAGPKHDNSEDVVMPRSQDASRTS SISPASQRRDSSIISPFDLRSDLSRLGSGQTELTLPSEPSEVERGTPTKFRRTSDLGSLAGIPAAMSQSSPVESTNI PSRTAIPATRPSTSEIKTASTNFAPGSDDVTACCLESIHSLPQANVQKRTFDNGTSWMSQSNDDSAQLALSDL STISKSLALTPVAMGLTNAMTGSGAVGDGWSISPWINMPQTPSDGALANTYTSASRAQADGHTVDTGMAV DGRKSLASRRGFSGSLKFEANAV

>UmbZIP_23.1

MAATQIARNSSFNGFDDIVKMPSSPQLQPRTRTISNAAAPSPSAGSTPAAAFNMRKVGLDESQPPPPPGSSP ETFFRYYLAVELRKAGTEPSEALLNRYVRNHFDSLFKNKSTATAPAPAPAAATPHAPSSSARPAAASALALAPSP AQQPHTSTSQVTVAAVARPPPPQPKSAVTVSAPVVATSSTTAHDTDNPPGLSPPELVPAESPALSCRSFSPVA TPRMLNNITEDDALVDFSSVFGEPSCSLLEVPSHTSSSQPIKFEDNSDALLSFFRDSPFRTTHYESATIDPHVVEN APIGGNSTRFSMPLASISPSDLSNVNAMASQNQSVTLSPNQLSVKQTDSAAPMSEDGMSEDDDDDDEDKQ NRSRHTSPSSKLACRPLSTIPMAVGDMNYLKPDPEVYKKLSSKEKRQLRNKISARNFRTRRKEYIGQLEDQIAD RDTLIEGLRQQISQLSVENKALKDEVKTIKARTISSQDVGKILEALQSMTGTSVAAAPTANIGTSGFGSGRASPT

QEAMPLTPGAVLNFGAADACTLSSSRPATPTSVAPGSPRSQSPRPSLLRANTKKDVAPQSSFWGGVGSGVGT
SNSFMPVC

>UmbZIP_23.2

MIALQSARPATASRPPAKPLSRNSPVRSTAKHHLEPNPFEEITPTTLSSLTNVNPTTAIDPNAPTPAAGSSLSTQ
PQHFAAKPTDAQNAFDLAFSRSFPNDRSDAKLTGSKLKAHVNATDSDRSVSHSISPQMVHKPLDAPNGGDQ
QNAASGLFLLSRAHKEMSKRDAASTSLYDSKPSSKKSTSAKNAPDAKPTSKPTANKRKKSNSTDDDAAATAQA
STSTNAPPAKAAKTAKGGKKAAAAAAAAAAAADASKDASTRANGGGSLKSDNGADDDDQHWDSDDENGE
GRGNMDEKRKNFLERNRQAALKCRQRKKAWLASLQAKVEYLQNDNENLQNTVGALRNENMFLKSQLVQA
TGGAPLPNIPMGLAMGMPMAPPPHGVDAQHHSMAPAPMGIPTSMAPHPAYLHPGVSQAPGGAYPPAR
APPPGFDPRQAGPTGRPRSESDALASNESSMPGLKHEREWSAASATDTTNKHTSAASGAATIKV

>UmbZIP_7.1

MAQTQRLEQIQNYRQLIAQLNATMPASADSTVPMLPFGASFADSAKTVGTGLNAASFGLNPMSADDPSWI
GHGFQAQAAVDFLSSPEWTDPSPALTDSMSFEIDSCGPSPLLPLDGYEDDLGVPGIAGAPLFPPQDGYASSN
VSPLEPAHNDFGSSTVGDFGAPEMGSDFTLFPETRPAKLPIEMAFSKLNKPAAGSGTSNEDPAMTLLRALSSA
ALSASSSTASVAVSNNSSTSVAASSTPVLTASPLAFTSMGAPQALAPSSTEATPPALQRTSLSLSSSSAPVATSES
RGTKRRMESTDLLPLDAPIQKRTYYTVSATSRRDTARAAEVDEFAEEEAAIRREKDPRVAKRLSNTLAARRSRH
RKAEELRLLNEKIEALTSEVESWKRRCEQAEKERNDALALLS

>UmbZIP_5.1

MAQILPNPATSSSSIAAQRNLAQRPLVSSSAAASSLKPAPSKPPSSRLAPAPSSSIAAAPLRSAAPSPPKPKPATL
APTLSAAPVPSTSTASPVAKKDTSGAPLVQPSKEWVLPARAKPGRKPSETEPLTKRKAQNRASQRAFRERKQS
YLAELEAKVAAYEAAEIDRSVEIQKVAQKLRAENDSLRKEVSAWKEKFAQIEKYLLQAKANGGRLPGITPSNRS
GPGCKAPAHERSSAVPKRGVLIAPGAKSERDRERSAGGVRFADAKPDKPALQAGSSTEEQQAASVPLRPKPIG
VSLPVPPPDAVDTIPSASLDDTASDDQGSLPETGKVAPRKASKLPLWSFESTKPPSPDAPSLTISATTSSDDKPT
SLLSPSLMPQGGHLSVGGGCGFCTEASPCVCADDFLDLSTQTPPSHIAVISDVASAVPLPKRRPASGASETSRR
MSIGSLTHANDLALDQRRRTASNGTGKKLWYTVTQPASPPGYSVTAAVPLSLKSTSKGNPSKKLWTVTETPAE
PVCTGDPSTCGACSTDPGLAAFCEAVTTATASPSSSVPSSAAATASSLSPSRPGMSRSGTTGQLLPPYPSRGETI
PSAWRQIRSHPRFSQWQGGLDLLAEVVSKRSGNVHSPLLSSKRPREASVEIEPSRSNTISATSNDRATAKPLLL
HTTSDTTVHTVPQIKTEDEDDGLDAREQKRRRILIDREAVQEALALLDAGTAAQPRAGASLSTNPGTGKEEQP
CPCPWRPGERRTP

>UmbZIP_9.1

MTSTTTSTPPMFAVAQASTPSSPSAFASSSRLSETPVKQETHHIALADACSNSSKKRKLIDERDDHDEDVDDSS
SSPSASPSKSSNTGGRRKASDEERKARLEARQARNRLSAQYSRERKKAYVETLEGSLNALKAENTLLRQQREQ
DQALQQTLDAKLKDAQLRVNTLETILRTLAPSLVPLLGPSSLLSLPSSSNPSVLATSPASSLAPQFDAGLSANLAS
ASVQDNSSASKEVPLSLAIAAPASTVSTPFSFGTLAQQSSNEGVRLPAADALTQFESVAGKQSALEKAGNNVD
VNGSSIVNQGYPLASQISVSAPEQPSSFAASAQVPLPQHVVEAKAAGVAREAEALLSNFIDLDAKLANGDAKT
AAQDQGAPTSATPSEPVSVGEGYAAAAGNALRSASAAAGDLASLSAASSADSGDGSSGSSDGSKTNNENGG
STIFNTISLSHADNKDQDLEKRFQLLTSPLLATERNVWELVTDTMLQDLYSGSDEVGAISGNADPALTPSEVD
MSAGSSPFDLVDLDIEIEEPLQVNIGLETEDAVHGGLLGFGHEKPDAGVPDWSGLMASIVA

>UmbZIP_16.1

MATTTSLGIGIWPSMSSGLTASHADTQSHRPRRNMHSEHHQQLHQQQHYLHRQYPAASAVLVPLDDVAAT
RAPHGGIRTSLAPYLTSEPASRPHRINTGNGSGAHESCTTARSSDVNVADNTHMLDSCLYFSALPPLSSHAKRR
GRSKMNAIDQAQQAAKLEQKSRRREQNRNAQRRLRDRKEDHVFKLEGEVAQLRQQDEQRCSKMRDLEEM
IRRLLGERQDLQTRLELQQGGETSLGLGNTLGLVDKSRMDRGENVGTESSTGSSRSNSNRRRGGTDSNSRSSS
HECCCVRIDIRDSYLPSPLAHSRREAKR

>UmbZIP_15.1

MVSVPTPAAAVAAAQNAPPSHDNAAKRKMSLSDIDDDSDSDSDSPDSSSKKLFSTGRTSASQQLVPVPGQP
DAPLVTKRTLQNRKAQREFRKRREARVRELEERCRRFDQMGLEANTELQRLAHRLKDENESLKSLIARLGFAH
MIPGIIESINNVEQPQHHHAQHPHHQHTAPHHPHAFAPLDIGMDAWASRASGMPITNAAPLPPHSEAPVQ
PSNAANAANAAAATNKKANRSHVPTTSASVPASTPISSLANLPGLTELLPVHAGGVNPSMLSDGNSSNRDSQ
RASGSSIAAVAVPAEPKSTTTTSRPIEDNSTFDNDWFAQLAQSGHDKDDNNVDAASTDNTALSRNVRSAAN
PILSLNFNNNSTDIKNTKSSNHNYHHHFSGSAMLGANARPFGLGLASALSLAQASSSGANMFPMPMPTQQN
GALLNPNPIPFAFNLSENKMPGEQSWWSQVAGNALEGDDSILDEKAQAVAQATGANQNGAPSPFDLSAFL
NGGLTPGGGGFTFGSQQTNDDTVIDAQKSTDNDDASKPSNGKGGQTQQGLTQAEHAQMFLRLLEAKMAK
GNTSTYAQLGFQPPMHHQQYAASQAGPSFSDASSSSSLSSSLSSSPDKEEDSALSPINIYTRLAQHPAFLSTNA
SELEELVDSISSSQQRQQQMARFQPPLSKRKGTTPTKVAPTLYPPPASALTPSIYAPNGAGPLAGTHTNDLEVD
EKAVNKMLGMLDRKSSKNAPAAGSALAF

>UmbZIP_1.1

MPFEESPNSFLFGLNQFTSPPADSQNDSANDSRSGSMLANLDAFAVADDVAGGSSQNDSTAAQAPNFADQ
LALWTNANFSFDGPTGHALLGDEEKEKEEAEHNRRREDENRRNQEEERERLARNAAASSRGSHAFRGKAREL
DSNTSGSAPPYPATSQPHTPTQHPHQQHHSHPSYFPGAPNFNAAVRPSPATNPPGPGVGGPFSLFNGFPNV
QQQQQQQSSQPGFGAPANLDMTSALALQHLLTSNPLALASLSQLAGLTNPQLQSHLAGIVNAGAALGGFN
QAQQATTPQQQHGHQPHSNGSVGPGASPWLSAQSLAASRGGSISGASPQNVNWAPQLGSSGLPAASPG
QVPPSFGSNLVIGPAPQFGSTANHANASNGPSSRVTDEPGSSASGTGQGSGPGSGSGSGSGSGSGSGSG
SGSGSGSGSGSGSGSAPSKKKRTSTSEGKKAQIDIDPDDSDDILSRLEDVERRYDIPPLKLIDTGNPEADAEANR
LAIEEDKRRRNTAASARFRIKKKQREAAMEQASKELQQRLADLETENARLRTENGWLKSLITVRPDQAMPGG
LDQSAVPNPFSNLAGPSGSSQRASGVSGAAAPAETAANGDAQLSRDTPDRQSGLHPRGVGTGGAASDENA
KGKANASKNSNANGSGAQLKRDREE

>UmbZIP_3.1

MPPAHRGPTGSPMDRWTDASPSAISSGFQIAMSLGPQPSLSGLSRSMTAASFANTDDEDEDDDDNDNDP
EGVSSTNSAAKKANTKTTKSKKATVTSAAATKGKDSRAAQLRKEQNRAAQREFRQRKQQYIRALEARVELLSS
DHDTQVNRLRFALRHLLAENNTLRGIVGNLAHFIGKRSIGGCLVESGMTMEMLEATMNSSSEKVMSEAWA
NWPGAGECEALKQIRKESNIPAEGLPESKLVNYFRDTNSKAANNASISDAANNSDKKKRTMDDAASKDAKRK
RNTDDLPPTAAPISTSNQPASNSAQSSPSTVFNVVTTAPTFTASPHNMSSIDDPSWQQQQQQQPSQIPYALP
NQFWQPAQGGFSRQDPGGVDSMFAQTLLGGGNANFSNLASLFPDQAAMNQNLFSQPDAAAASFNGLVS
TNSSNGDGAVTNSNPASNFLFFGAGLLPSTSSFAPTPAAADLATSVAPGGGASGGTLVMPSRATGGIKTIMLP
SSSRLPPEHVRALRRRYARCVAQVNRLWAKRGATSFLNLPNDYQLDEEDIRFIEQQEATNPHVKLAAIRLPNN
WDEGDFTSNGKSGAPQGSSEAESAYDNNDKADKFLQLAYHMNNYRINAHYNLPPSLKPTSLQRSTPHDPAI

DGMPWPSIRDKMIQMPQLEPHSVIIDLIRFLAVFNGDPNSEKTWVLTLPFLIRYPQLADAALLANTNAHRATR
GEPEVTMDDVWKEHHRFREYTQAKLKEMN
