# Supplementary material for: Genome-Wide Identification and Expression Analysis of the Basic Leucine Zipper (bZIP) Transcription Factor Gene Family in Fusarium graminearum
Source: Genes (Basel). 2022 Mar 28;13(4):607. doi: 10.3390/genes13040607 (PMC9028111; doi:10.3390/genes13040607)
Supplement: Supplementary file 1 [file genes-13-00607-s001.zip › Supplementary Files/File S3 Primers.pdf]

Ubc-F= TCCCCTTACTCTGGCGGTGTC
Ubc-R= TTGGGGTGGTAGATGCGTGTAGT

1.1-F	CAGCAACAACCCCTTCATGC
1.1-R	TGCCAGGGTTTGTGAATGGT
1.2-F	TGTACCGACAACAGACAGGC
1.2-R	GTCGAAGAGATCGGCCTCAG
1.3-F	CGAACCTACGACCACCACTC
1.3-R	TCTGGGGTTGTGTTGGGGTA
1.4-F	AGACTTTCGGTTTCGGGCAT
1.4-R	TCATTTTCTCCTCGGGCACC
1.5-F	ATCTCCCAAGCCAAAGAGGC
1.5-R	ATGGCCTGTTCCTGAGAAGC
1.6-F	GTGAGTCGAAGTCCCCCAAG
1.6-R	TTTGGCTTGTTCGGCATTGG
1.7-F	GCTAGAAGACCGGCACAGAG
1.7-R	CTCCCGATGGTGATGCAGTT
2.1-F	CAGGCCGCTAACCATGAGAA
2.1-R	CGAGGCAGAGATTTGGTGGT
2.2-F	CAAGGCTTCAACGCCAATCC
2.2-R	AGACCCGTTTCTCATCGAGC
2.3-F	CCAAGCCGACGACAAAAGTG
2.3-R	CTCCACTCGCTCGCTATCAG
2.4-F	AGCCACCTATGACACATCGC
2.4-R	CGAAGTGCAAGCAAAAGCCA
2.5-F	ATCTGAGTGGAGCAATGGGC
2.5-R	ACACCGTATCTTGGCAGACG
2.6-F	AACACCACTGTCGATCCCAC
2.6-R	GTTGTGTCAGGTCGGGAGAG
3.1-F	GACCGAGAACAAGTGGCTCA
3.1-R	CTTGACGGAGGACTTGGCTT

3.2-F	ACCCTTCCTCCAATCTCCCA
3.2-R	GACCACAACCACTTGAACGC
3.3-F	GGGCTTTACATTCCACCCCA
3.3-R	TACTGCTCGCCGTTTGTAGG
4.1-F	ATGGGTTATGGCGGTGAAGG
4.1-R	TGACGCTCGCAGGAGAATAC
4.2-F	GCACTGGGAATGGACTTGGA
4.2-R	GCACCAAGTGAAGAATCGGC
4.3-F	ATTCAGCCGTCACCCTCAAG
4.3-R	CGTTCTCTGCCTCCTTCCTC
4.4-F	CAACCCACACAACAACCACC
4.4-R	GTTCGGTAGGAGCAGAAGCA
4.5-F	GAGTAGCCCAGTTGCTCCAG
4.5-R	GTGGTAGATGACGAAGGGGC
4.6-F	AGCAGAACCACCAAGACTCG
4.6-R	CTGGACCCGTTTTGACGGTA
