# Supplementary material for: Genome-Wide Identification and Expression Analysis of the Basic Leucine Zipper (bZIP) Transcription Factor Gene Family in Fusarium graminearum
Source: Genes (Basel). 2022 Mar 28;13(4):607. doi: 10.3390/genes13040607 (PMC9028111; doi:10.3390/genes13040607)
Supplement: Supplementary file 1 [file genes-13-00607-s001.zip › Supplementary Files/File S5 Synteny.pdf]

F.graminearum	*Neurospora crassa*
Chr#1	*Chr#4*
XM_011319470	XM_011395985
XM_011319474	XM_957580
XM_011318112	XM_958726
XM_011318098	XM_959251
XM_011318103	XM_958726
XM_011326298	XM_958707
Chr#1	*Chr#1*
XM_011318111	XM_958727
XM_011318107	XM_958728
Chr#1	*Chr#5*
XM_011320893	XM_955692
XM_011320770	XM_956333
XM_011319394	XM_956364
XM_011320147	XM_951102
XM_011319394	XM_956364
XM_011319394	XM_956364
XM_011320872	XM_955827
Chr#1	Chr#6
XM_011321982	XM_011396656
XM_011320872	XM_955419
Chr#1	*Chr#3*
XM_011320129	XM_957827
XM_011320246	XM_952143
XM_011320147	XM_951102
XM_011319394	XM_956364
Chr#1	*Chr#2*
XM_011318934	XM_952075
XM_011318913	XM_951174
Chr#2	Chr#1
XM_01132154	XM_959423
XM_011321542	XM_959422
XM_011321544	XM_011395036
XM_011321546	XM_011395037
XM_011321926	XM_959788
XM_011321931	XM_959781
XM_011321448	XM_960440
XM_011321527	XM_959251
Chr#2	Chr#4
XM_011323344	XM_957472
XM_011323297	XM_952793.2
XM_011321463	XM_959022
XM_011323078	XM_011396020
Chr#2	Chr#3

XM_011323219	XM_951602
Chr#2	Chr#5
XM_011322806	XM_952301
Chr#2	Chr#5
XM_011322806	XM_952301
Chr2	Chr#6
XM_011321542	XM_011321796
XM_011323034	XM_952513
Chr#3	Chr#4
XM_011325174	XM_957485
XM_011325255	XM_011396080
XM_011326508	XM_956877
XM_011325901	XM_956742
Chr#3	Chr#3
XM_011325613	XM_951913
XM_011325662	XM_951078
XM_011325662	XM_951078
XM_011325731	XM_951502
Chr#3	Chr#1
XM_011326005	rna-XM_959841
Chr#3	Chr#7
XM_954411	XM_011326050
XM_011327279	XM_011396831
Chr#3	Chr#2
XM_011325479	XM_958430
XM_011326624	XM_951786
XM_011325662	XM_951078
XM_011325360	XM_950742
XM_011327185	XM_950825
Chr#3	Chr#7
XM_011326050	XM_954411
Chr#4	Chr#4
XM_011320445	XM_952387
Chr#4	Chr#1
XM_011328514	XM_958693
XM_011328507	XM_953390
XM_011328184	XM_958786
XM_011330137	XM_011394664
Chr#4	Chr#5
XM_011328720	XM_956022
XM_011328841	XM_955692
Chr#4	Chr#3
XM_011328673	XM_951078
XM_011328841	XM_011395696
Chr#4	Chr#7
XM_011328237	XM_954334

XM_011328840	XM_952886
XM_011330053	XM_954702
XM_011329499	XM_950825
XM_011329486	XM_954411
Chr#4	Chr#6
XM_011330053	XM_955274
XM_011328673	XM_952456
XM_011329486	XM_955135
