# Supplementary figures and images for: Genome-Wide Identification and Expression Analysis of the Basic Leucine Zipper (bZIP) Transcription Factor Gene Family in Fusarium graminearum
Source: Genes (Basel). 2022 Mar 28;13(4):607. doi: 10.3390/genes13040607 (PMC9028111; doi:10.3390/genes13040607)

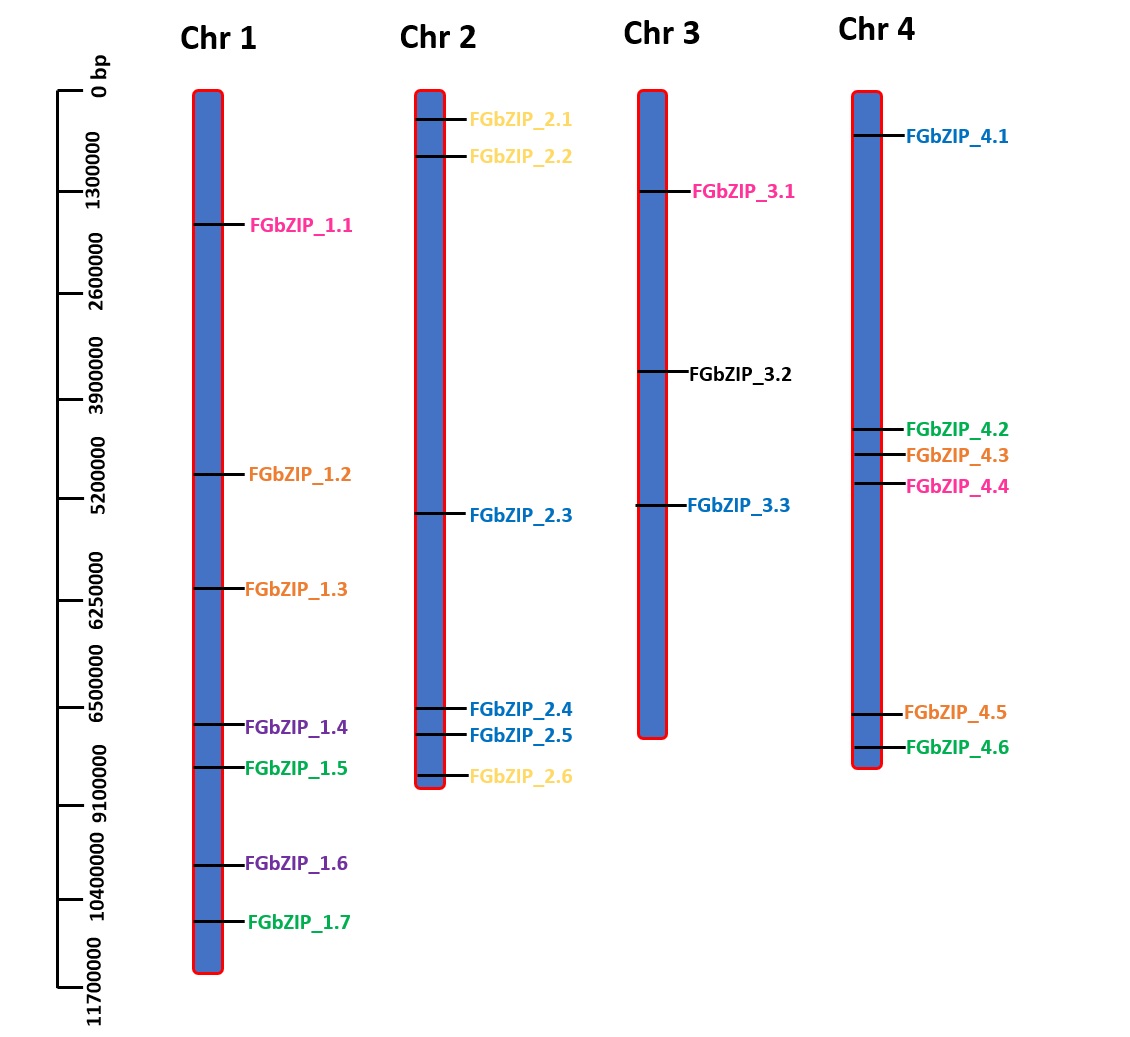

Supplement: Supplementary file 1 [file genes-13-00607-s001.zip › Supplementary Figures/Figure S1..jpg]

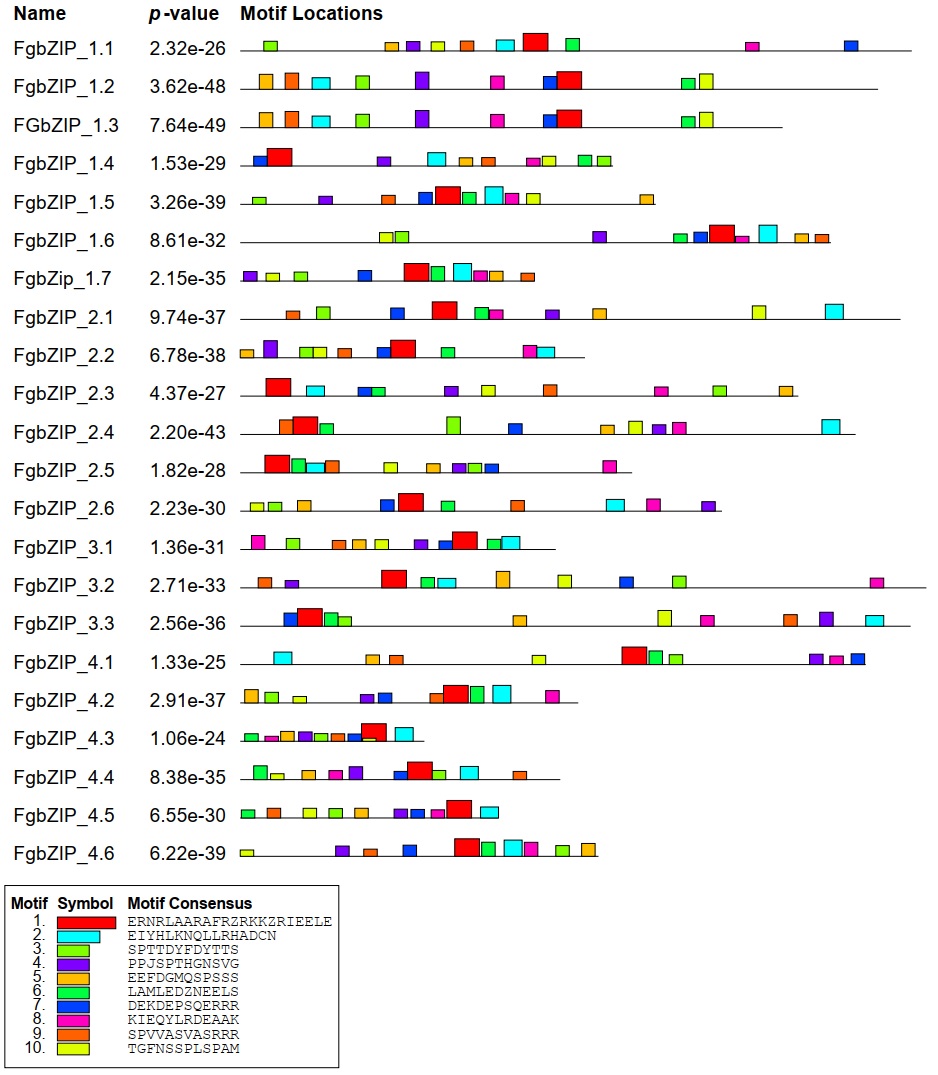

Supplement: Supplementary file 1 [file genes-13-00607-s001.zip › Supplementary Figures/Figure S2..jpg]

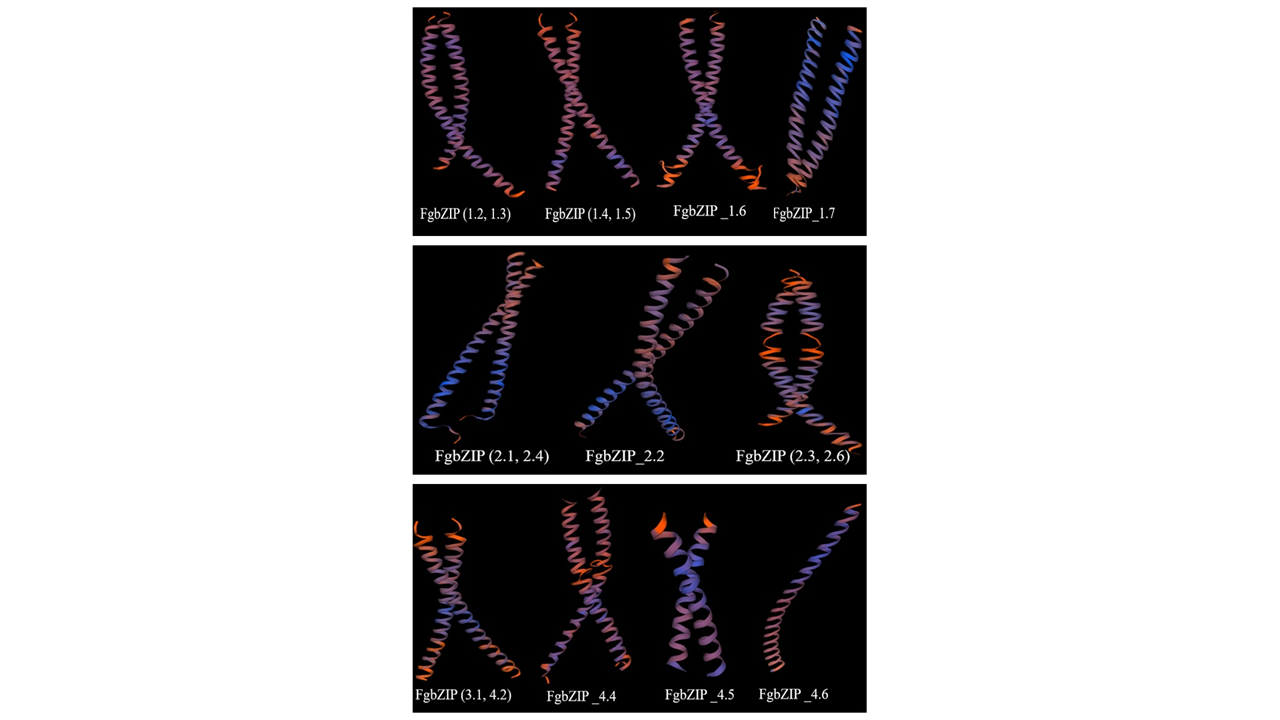

Supplement: Supplementary file 1 [file genes-13-00607-s001.zip › Supplementary Figures/Figure S3..tif]

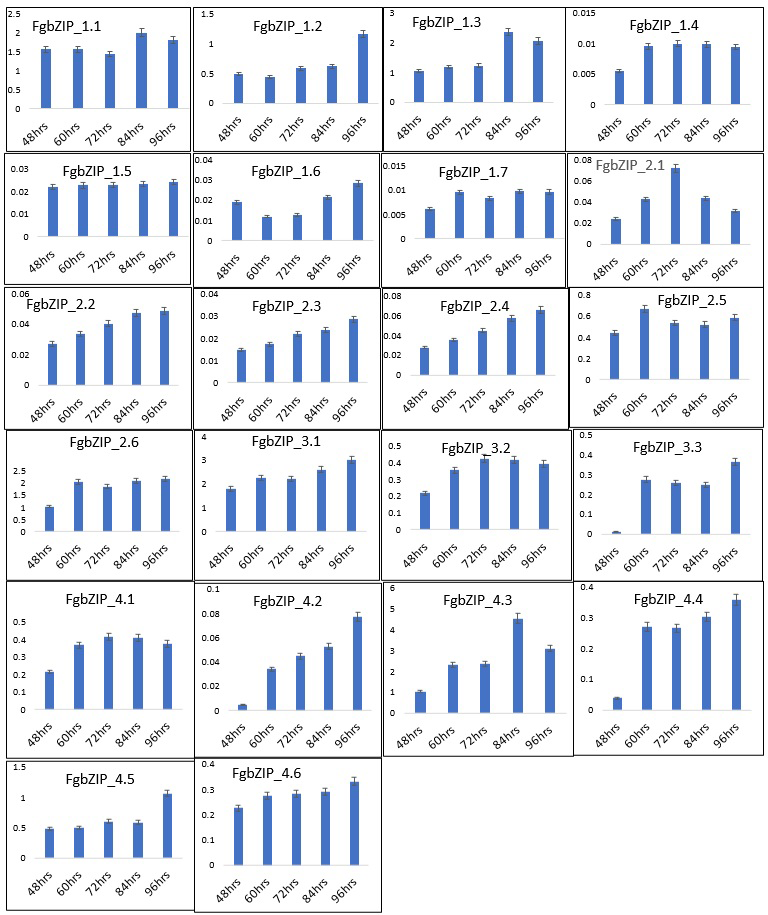

Supplement: Supplementary file 1 [file genes-13-00607-s001.zip › Supplementary Figures/Figure S4..tif]

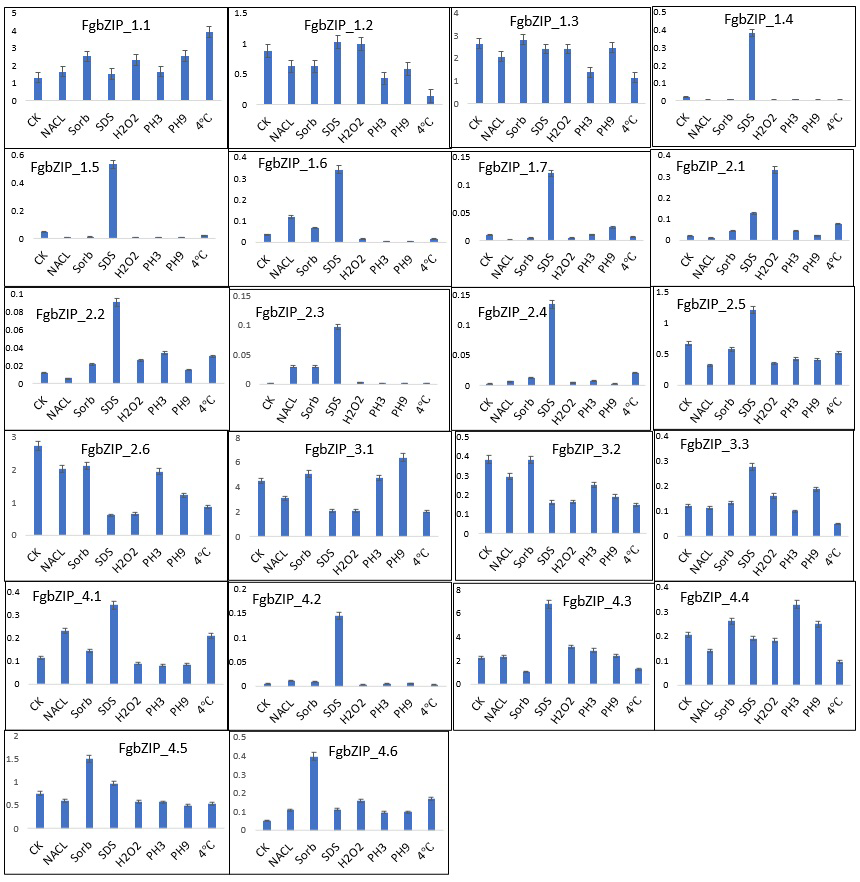

Supplement: Supplementary file 1 [file genes-13-00607-s001.zip › Supplementary Figures/Figure S5..tif]
